# Changes in the Gut Microbiome Associated with Intussusception in Patients with Peutz-Jeghers Syndrome

**DOI:** 10.1128/spectrum.02819-22

**Published:** 2023-01-31

**Authors:** Zhiqing Wang, Liping Liang, Le Liu, Zhi Wang, Ying Wang, Zonglin Yu, Baoping Wu, Ye Chen

**Affiliations:** a Department of Gastroenterology, State Key Laboratory of Organ Failure Research, Guangdong Provincial Key Laboratory of Gastroenterology, Nanfang Hospital, Southern Medical University, Guangzhou, China; b Department of Gastroenterology, Integrative Microecology Center, Shenzhen Hospital, Southern Medical University, Shenzhen, China; University of Georgia

**Keywords:** Peutz-Jeghers syndrome, gut microbiota, intussusception, biomarkers, functional analysis

## Abstract

Peutz-Jeghers syndrome (PJS) is a rare hereditary disorder characterized by intestinal polyposis, and intestinal intussusception is one of the most urgent complications. While it is known that imbalance of the gut microbiota is highly associated with intestinal disorders, the role of the gut microbiome in the pathogenesis of PJS has not been reported. In this study, we performed 16S rRNA sequencing on stools from 168 patients and 68 healthy family members who lived together to determine the gut microbiome composition of PJS patients. Metagenomics sequencing was further performed on the representative samples (61 PJS patients and 27 healthy family members) to analyze the functional changes. We found that the fecal microbiome of patients with PJS showed a greater variation in β-diversity. An enhancement of Escherichia coli and a reduction of Faecalibacterium prausnitzii was identified in PJS patients. Further reduction of Faecalibacterium prausnitzii was the characteristic microbial change observed in patients with intussusception. Functional analysis revealed that the abundance of propanoate metabolism was enriched in PJS patients and further enriched in those with intussusception. Escherichia coli was the major contributor to the enrichment of this metabolism pathway, which was associated with the abnormal expression of methylglyoxal synthase (encoded by *mgsA*) and phosphate acetyltransferase (encoded by *pta*). Our findings showed a distinct gut microbiome signature in PJS patients and identified the connection between the gut microbiome and intussusception. Alterations in the gut microbiome might be involved in the pathogenesis of PJS and may serve as biomarkers for gastrointestinal surveillance.

**IMPORTANCE** Recent research has established a link between the gut microbiome and polyps and neoplasia, and antibiotic use influences the microbiome and the development of colorectal polyps. Familial adenomatous polyposis (FAP), which is characterized by the early development of benign precursor lesions (polyps), is associated with enterotoxigenic Bacteroides fragilis and Escherichia coli biofilms. However, the relationship between the gut microbiome and the pathophysiology of PJS has not yet been established. In this study, we found that PJS patients had a distinct microbiome composition, with a greater variation in β-diversity, an increase in Escherichia coli, and a decrease in Faecalibacterium prausnitzii. A further reduction of Faecalibacterium prausnitzii was observed in patients with intussusception. Moreover, PJS involved increased propanoate metabolism as well as abnormal *mgsA* and *pta* expression. These findings may contribute to a better understanding of the etiology of PJS and improve disease control strategies.

## INTRODUCTION

Peutz-Jeghers syndrome (PJS) is a rare hereditary intestinal polyposis condition with an estimated incidence of approximately 1 in 50,000 to 1 in 100,000 (1). Germ line mutations in the serine/threonine kinase 11 (STK11) tumor suppressor gene was found to be associated with PJS (2). Mucocutaneous melanin pigmentation and gastrointestinal hamartomatous polyps are two major essential clinical features of PJS. In particular, hamartomatous polyps associated with PJS can be found throughout the gastrointestinal tract, predominantly in the small intestine and the colon (3). Even at a young age, the presence of pathological hamartomatous polyps in PJS patients may lead to diverse gastrointestinal complications, including hemorrhage, anemia, abdominal pain, intussusception, obstruction, and infarction (4). Among them, small intestinal intussusception is the most urgent and even lethal gastrointestinal complication and produces a substantial impact on the clinical prognosis of PJS patients (5). Over 90% patients require surgical treatment, mainly laparotomy, when presenting with intussusception (6). Gastrointestinal surveillance by regular gastrointestinal endoscopy is an imperative management strategy to limit the risk of emergency laparotomy in patients affected by PJS (7–9). However, gastrointestinal endoscopy is an invasive procedure which carries a risk for complications. Therefore, it is necessary to find a noninvasive, safe, and reliable approach to monitor disease progression in PJS patients.

STK11, also known as live kinase B1 (LKB1), is detected to be mutated in PJS and a wide variety of tumors (10, 11). LKB1 is primarily dependent on the phosphorylation and activation of the AMP-activated protein kinase (AMPK) for its actions. As a vital metabolic regulator, AMPK modulates cellular metabolism by regulating metabolic enzyme activities and activating adaptive transcriptional responses (12, 13), which might be involved in the pathogenesis of PJS.

Emerging evidence suggests that the gut microbiome is highly linked with the development of a wide spectrum of gastrointestinal disorders, especially polyposis and neoplasia (14–16). A case-control study conducted in Sweden recently reported that usage of broad-spectrum antibiotics, such as tetracyclines, β-lactam antibacterials, and sulfonamides, could affect the composition of the intestinal microbiome and was related to an increased risk of colorectal polyps (17). The pathogenesis of familial adenomatous polyposis (FAP), a disorder characterized by mutation in the adenomatous polyposis coli (APC) gene, was found to be associated with bacterial biofilms constituted primarily of Escherichia coli and enterotoxigenic Bacteroides fragilis (18). Patients with adenomas have greater abundances of proinflammatory genera, which may produce genotoxic or inflammatory metabolites such as H_2_S and secondary bile acids, thus promoting adenoma development and tumorigenesis (19). Compared to serrated polyp, conventional adenoma displayed more severe gut microbiota dysbiosis characterized by a reduction of commensal bacteria and an enhancement of potentially harmful bacteria (20). In addition, significant alteration in microbiome and metabolome was also observed in multiple polypoid adenomas and intramucosal carcinomas, which were considered the early stages of colorectal cancer (CRC) (21). Therefore, it is probable that the gut microbiome influences the development of PJS and provides information for prognostic evaluation. So far, however, the influence of the intestinal microbiome in the pathophysiology of PJS has received little attention.

To expand upon the understanding of the modification of gut microbiome composition and the link between the gut microbiome and intussusception in PJS, 16S rRNA gene sequencing was performed on a Chinese family cohort containing 168 PJS patients and 68 healthy family members. Metagenomic sequencing of the gut microbiota was further performed for 61 PJS patients and 27 healthy family members to investigate the functional changes associated with intussusception. Aware that AMPK signaling pathway was associated with the pathogenesis of PJS, we analyzed specific species contributions to this pathway.

## RESULTS

### Study population.

A total of 168 PJS patients (average age, 26.11 ± 13.03; ratio of males to females, 91:77) and 68 healthy family members (average age, 32.54 ± 18.26; ratio of males to females, 38:30) were included for analysis. Demographics and clinical characteristics are shown in Table 1. Of the 168 PJS patients, gastrointestinal polyps were detected in 147 (88%); polyps were found throughout the gastrointestinal tract in 93 patients (63%). A total of 106 patients (63%) had experienced at least one intussusception, and the mean age of the first intussusception was 15.68 years (15.68 ± 8.47). Furthermore, 97 of these 106 cases (92%) had a personal history of surgery as the therapy for intussusception.

**TABLE 1:** Characteristics of study participants

Characteristic^*a*^	PJS (*n* = 168)^*b*^	Healthy family members (*n* = 68)^*b*^	*P* value^*c*^
Age (yrs)	26.11 ± 13.03	32.54 ± 18.26	0.0028
Gender (male/female)	91/77	38/30	
BMI (kg/m^2^)	20.79 ± 4.38	22.92 ± 4.99	0.0014
Region (south/north)	98/70	41/27	
Mucocutaneous pigmentation	158 (94%)		
Gastrointestinal polyps	147 (88%)		
Intussusception/obstruction	106 (63%)		
History of surgery	97 (58%)		
Family history of PJS	97 (58%)		
Cancer	11 (6%)		

a PJS, Peutz-Jeghers syndrome; BMI, body mass index.

b Data are presented as means ± SD or number (percent).

c *P* values were determined by unpaired *t* test.

### Alterations of gut microbiota profiles in PJS patients compared with healthy family members based on the 16S rRNA data.

**FIG 1:**
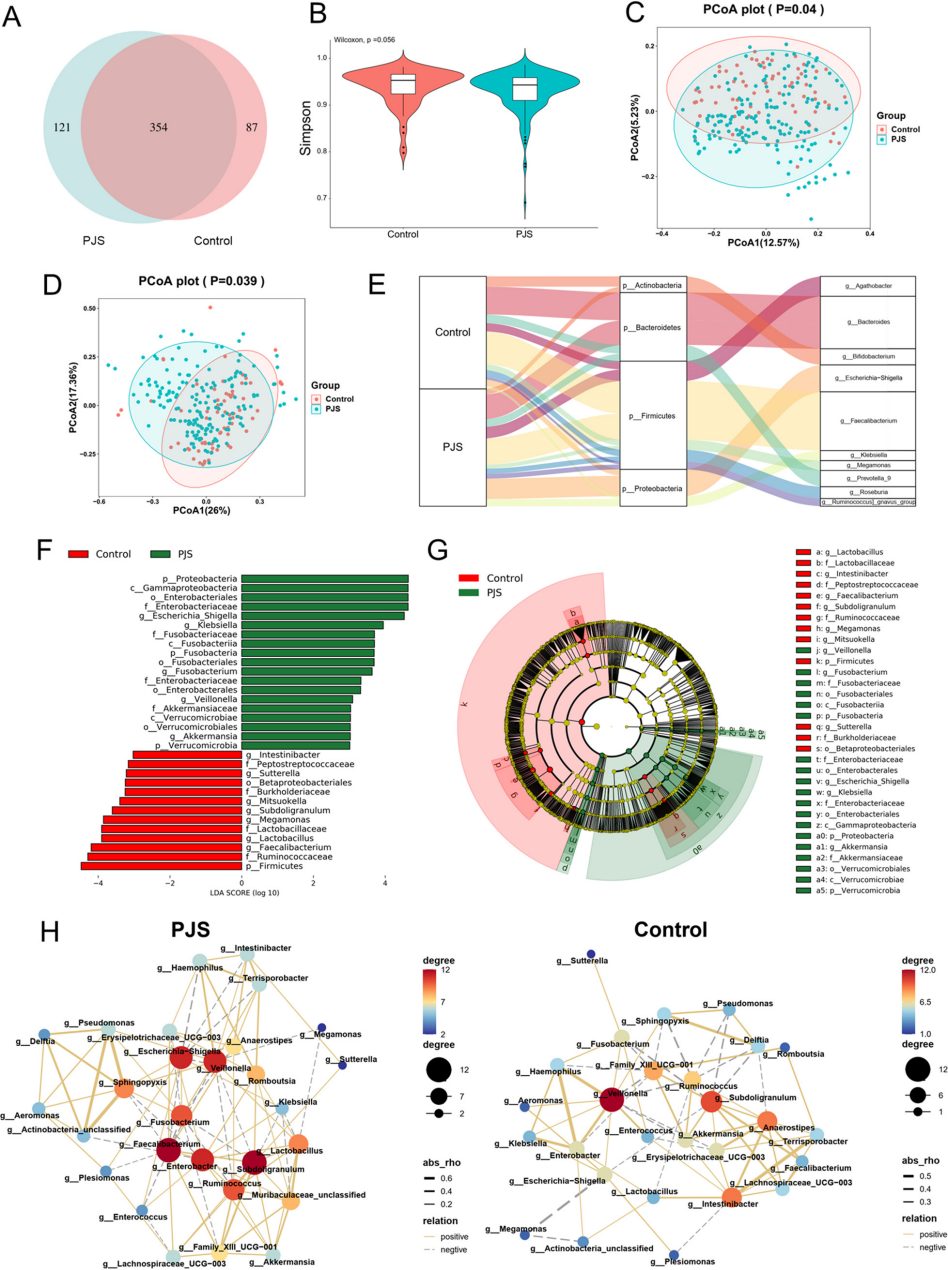
Alteration of gut microbiota in PJS patients and healthy family members according to the 16S rRNA sequencing. (A) Venn diagram constructed to identify the unique and shared genera. (B) Comparison of α-diversities based on Simpson index between PJS patients and healthy controls. (C and D) PCoA based on the unweighted (C) and weighted (D) UniFrac distance metrics for PJS patients and healthy controls. (E) Sankey diagram of dominant taxa (phyla and genera) between PJS patients and healthy controls. (F) LEfSe analysis identified the differentially abundant taxa between PJS patients and healthy controls (LDA > 3.0). (G) Cladograms produced from LEfSe demonstrating bacterial taxonomic differences between PJS patients and healthy controls. (H) Co-occurrence network inferred from the relative abundances of the top 30 genera significantly different between PJS patients and healthy controls based on the Spearman correlation algorithms. Each node displays a distinct bacterial genus. Size and hue of the nodes show the relative abundance of each genus. Yellow full lines represent positive interactions between nodes, whereas gray dashed lines represent negative interactions. Line thickness represents increasing Spearman’s rho (correlation coefficient).

To obtain an overview of the microbiome profiles in PJS patients, we applied 16S rRNA gene sequencing, and an average of 64,040 optimized reads per individual (range, 45,242 to 129,374) were obtained. An adequate sequencing depth was assessed according to rarefaction curves generated from the observed operational taxonomic units (OTUs) (see Fig. S1A in the supplemental material). A Venn diagram shows 121 unique genera in PJS patients and 87 unique genera in healthy family controls, while 354 genera were shared by the two groups (Fig. 1A). At the community level, the Simpson index revealed no significant differences in α-diversity between the two groups (*P = *0.056) (Fig. 1B). To evaluate the similarity of microbial community (β-diversity) between the two groups, principal-coordinate analysis (PCoA) was performed based on unweighted (Fig. 1C) and weighted (Fig. 1D) UniFrac distances to visualize the differences in bacterial communities. The analysis demonstrated that the two groups clustered separately in terms of fecal microbiota composition and that there was greater person-to-person microbiome variability among PJS patients.

The dominant taxa at the phylum level included *Firmicutes*, *Bacteroidetes*, *Proteobacteria*, and *Actinobacteria* (Fig. S1B). The abundances of *Proteobacteria*, *Fusobacteria*, *Verrucomicrobia*, *Cyanobacteria*, and Chlamydia were higher in the PJS group (*P < *0.001), whereas the abundance of *Firmicutes* was higher in the control group (*P = *0.01). Furthermore, the dominant taxa at the genus level included *Faecalibacterium*, *Bacteroides*, Escherichia-*Shigella*, *Agathobacter*, and *Prevotella_9* (Fig. S1C). Genus-level comparisons revealed that 29 genera were significantly enriched in the PJS group, while 63 genera were significantly enriched in the control group (Table S1). A Sankey diagram shows the major proportions of dominant taxa (phyla and genera) in PJS patients and healthy family controls (Fig. 1E).

To identify the profiles of the gut microbiota associated with PJS, linear discriminant analysis (LDA) effect size (LEfSe) analysis was performed. LEfSe analysis showed a total of 31 discriminative features (LDA > 3; *P < *0.05 [Fig. 1F]) at the phylum (*n* = 4), class (*n* = 3), order (*n* = 5), family (*n* = 7), and genus (*n* = 12) levels. The relative abundance of phyla *Proteobacteria*, *Fusobacteria*, and *Verrucomicrobia* were higher in PJS patients, whereas the phylum *Firmicutes* was significantly enriched in healthy controls (Fig. 1F). At the family level, PJS enriched organisms included *Enterobacteriaceae*, *Fusobacteriaceae*, and *Akkermansiaceae*, while *Ruminococcaceae*, *Burkholderiaceae*, *Lactobacillaceae*, and *Peptostreptococcaceae* were prevalent in the control group. The genera Escherichia, *Shigella*, Klebsiella, *Fusobacterium*, *Veillonella*, and *Akkermansia* were more abundant in PJS patients, while *Faecalibacterium*, *Lactobacillus*, *Megamonas*, *Subdoligranulum*, *Mitsuokella*, *Sutterella*, and *Intestinibacter* were significantly more abundant in healthy individuals (Fig. 1G).

To characterize the interaction patterns of differentiated microbial taxa, we next constructed co-occurrence networks of top 30 genera from genus-level comparisons between the two groups (Table S1) based on significant Spearman correlations. Notably, microbial community in the PJS group displayed a co-occurrence network with a more complicated correlation among genera. *Faecalibacterium*, *Subdoligranulum*, Escherichia-*Shigella*, *Veillonella*, and Enterobacter played a crucial role in the microbial community of PJS patients (Fig. 1H). Overall, these results demonstrated that microbial composition and correlation network in the PJS group displayed alteration compared with healthy controls, further indicating the presence of gut microbiota dysbiosis in PJS patients.

### Association of microbiome composition with intussusception.

**FIG 2:**
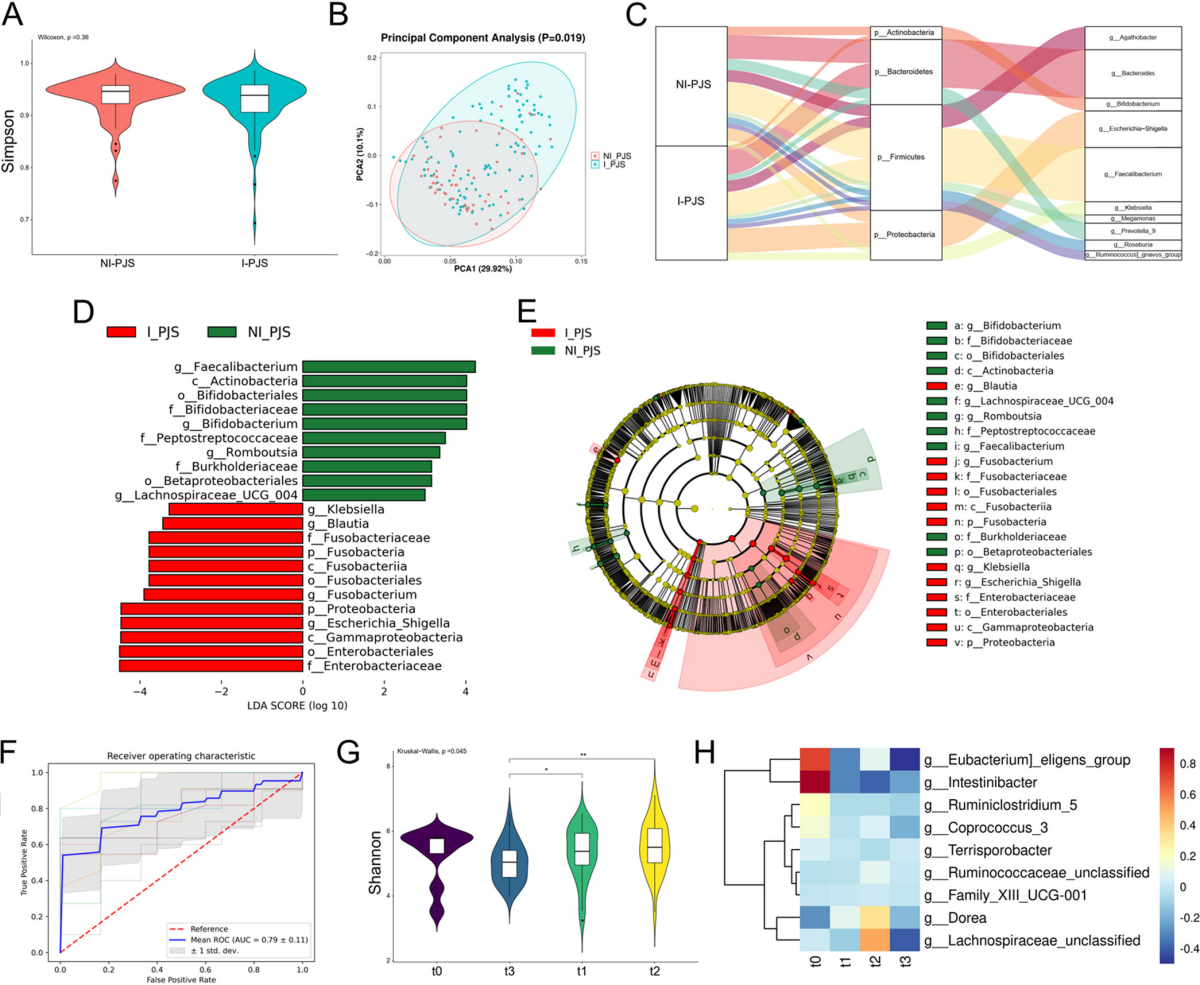
Relationship between gut microbiome composition and intussusception. (A) Comparison of α-diversities based on Simpson index between I-PJS and NI-PJS patients. (B) PCoA based on weighted UniFrac distance metrics between I-PJS and NI-PJS patients. (C) Sankey diagram of dominant taxa (phyla and genera) between I-PJS and NI-PJS patients. (D) LEfSe analysis identified the differences in abundance between I-PJS and NI-PJS patients (LDA > 3.0). (E) Cladograms obtained from LEfSe demonstrating phylogenetic distribution of taxonomic differences between I-PJS and NI-PJS patients. (F) Twenty genera were selected from the random forest model; the corresponding ROC curve is shown, with an AUC value of 0.79 ± 0.11. (G) Difference in α-diversity based on Shannon index among PJS patients separated by surgery times. (H) Heat map of the relative abundances of the 9 genera that differentiated among PJS patients separated by surgery times. Data were evaluated by the Wilcoxon rank sum test. *, *P < *0.05; **, *P < *0.01.

Intussusception is the severe clinical outcome of PJS patients which is characterized by polyps throughout the gastrointestinal tract. To identify the effect of intussusception on the gut microbiome, we further compared the gut microbiome composition of 106 PJS patients who had undergone at least one intussusception (I-PJS) with that of 57 PJS patients who did not experience intussusception (NI-PJS). The remaining 5 PJS patients did not provide information related to the experience of intussusception and were therefore not included in this subgroup analysis. The microbial communities did not show significant differences for the α-diversity indices among PJS without and with intussusception (Fig. 2A). Principal-component analysis (PCA) revealed that the microbiome profile of the I-PJS group was distinct from that of the NI-PJS group (*P = *0.019) (Fig. 2B).

The composition of the microbiome at the phylum level further showed differences between the NI-PJS group and the I-PJS group (Fig. S2A). The proportions of *Proteobacteria*, *Fusobacteria*, and *Synergistetes* were increased in I-PJS patients compared to the NI-PJS group (Fig. S2B). Compositional change of gut microbiota in I-PJS patients is also presented at the genus level (Fig. S2C). Genus-level comparisons revealed that 11 genera were substantially elevated in the I-PJS group, while 17 genera were substantially elevated in the NI-PJS group, exhibiting a similar pattern of alteration between PJS patients and healthy controls (Fig. S2D). For instance, the increased Escherichia-*Shigella* and the decreased *Faecalibacterium* in I-PJS compared to NI-PJS also displayed the same trend in the PJS patients compared to healthy controls. In Fig. 2C, the proportional abundances of dominant taxa in I-PJS and NI-PJS patients at the phylum and genus levels are depicted in a Sankey diagram. LEfSe analysis and a cladogram (shown in Fig. 2D and E) further uncovered additional hallmark microbial profiles and main bacterial biomarkers of I-PJS patients as indicated by LDA (LDA score > 3). At the phylum level, the abundance of *Proteobacteria* and *Fusobacteria* was enriched in I-PJS patient samples compared to that in the NI-PJS group. At the family level, I-PJS enriched taxa consisted of *Enterobacteriaceae* and *Fusobacteriaceae*, whereas *Bifidobacteriaceae*, *Peptostreptococcaceae*, and *Burkholderiaceae* were prevalent in the NI-PJS group. In addition, genera such as Escherichia-*Shigella*, *Fusobacterium*, *Blautia*, and Klebsiella were substantially more abundant in the I-PJS group than in the NI-PJS group. In contrast, *Faecalibacterium*, *Bifidobacterium*, and *Romboutsia* were significantly less abundant in I-PJS patient samples than in NI-PJS samples (Fig. 2D and E). To investigate the prediction potential of the intestinal microbiome in distinguishing PJS with and without intussusception, the random forest model was constructed on the genus abundance data set (Fig. S2E). The combination of these 20 genera achieved good diagnostic performance, with an area under the curve (AUC) of 0.79 (Fig. 2F).

Surgery is the mainstay of therapy in PJS patients with intussusception. Among the 106 PJS patients who experienced at least one intussusception, 9 (8%) did not undergo surgery, 33 (31%) had a single surgery, 31 (29%) had two surgeries, and 33 (31%) had more than two surgeries. When classified by the number of surgeries (0 [t0] versus 1 versus 2 versus ≥3), the Shannon index of the t3 subgroup was decreased compared to those of the t1 and t2 subgroups (Fig. 2G). Moreover, different microbial profiles in these subgroups were identified at the genus level. The Eubacterium eligens group and *Intestinibacter* were markedly decreased in patients that had undergone surgery, while *Dorea* and *Lachnospiraceae_unclassified* were increased in patients that had undergone surgery (Fig. 2H).

Based on the above-described analysis, the compositions of gut microbiotas across PJS patients with and without intussusception showed statistical differences, which may serve as significant predictors of intussusception, a critical complication in PJS patients.

### Metagenomic data exhibited significant differences between PJS patients and healthy controls.

**FIG 3:**
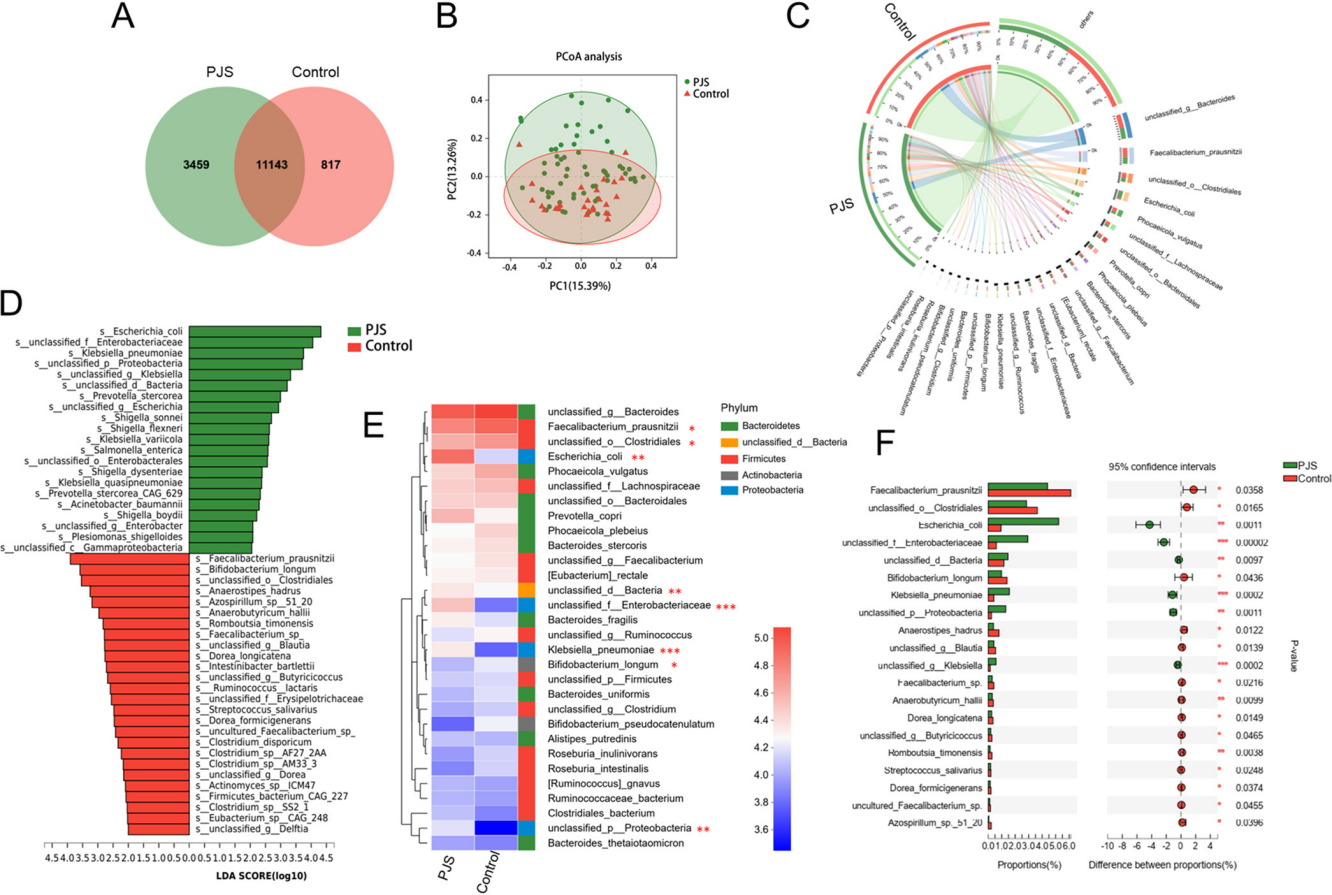
Metagenomic characteristics of PJS patients relative to healthy family members. (A) Venn diagram showing the number of the unique and shared species between PJS patients and healthy controls. (B) PCoA based on Bray-Curtis distance at the species level revealed structural clustering. Permutational multivariate analysis of variance (PERMANOVA), *P = *0.002. (C) Circos diagram of dominant species between PJS patients and healthy controls. (D) LEfSe analysis identified the differential species between PJS patients and healthy controls (LDA > 2.0). (E) Heat map of the relative abundances of the top 30 species across two groups. (F) Extended error bar plot demonstrating the top 20 significantly different species between PJS patients and healthy controls. Data were evaluated by the Wilcoxon rank sum test. *, *P < *0.05; **, *P < *0.01; ***, *P < *0.001.

To further determine the relationship between gut microbial alterations at the species level and functions of the gut microbiome in PJS patients, shotgun metagenomic sequencing was performed on fecal samples from 61 PJS patients (35 with intussusception and 26 without intussusception) and 27 healthy family members. A total of 22,311,093 genes were evaluated, and 2,514,259 genes were grouped into a nonredundant gene catalogue for further study (Fig. S3A). A Venn diagram shows 3,459 unique species in the PJS group and 817 unique species in the control group, while 11,143 species were shared by the two groups (Fig. 3A). PCoA, based upon Bray-Curtis distance at the species level, showed significant dissimilarity in bacterial communities between the PJS and control groups (*P = *0.002) (Fig. 3B). The composition of the microbial community also revealed striking variations at the species level (Fig. 3C). LEfSe analysis was further conducted to explore relative taxon abundances between two groups, which were characterized by significant difference (LDA score > 2). Twenty-one species were enriched in the PJS group, among which Escherichia coli was the most abundant bacterial species, followed by *unclassified_f _Enterobacteriaceae* and Klebsiella pneumoniae. Moreover, 26 species were enriched in the control group, among which Faecalibacterium prausnitzii was the most abundant bacterial species, followed by Bifidobacterium longum and *unclassified_o_Clostridiales* (Fig. 3D). Consistent with the 16S rRNA analysis, the majority of the differentially identified species in the PJS group belonged to the genera Klebsiella and Escherichia-*Shigella* as well as the family *Enterobacteriaceae*. A community heatmap of the top 30 species is shown in Fig. 3E. Differential abundance analysis further revealed significant differences of 995 species between the two groups (Wilcoxon rank sum test [Table S2]). The top 20 differential species are shown in Fig. 3F.

**FIG 4:**
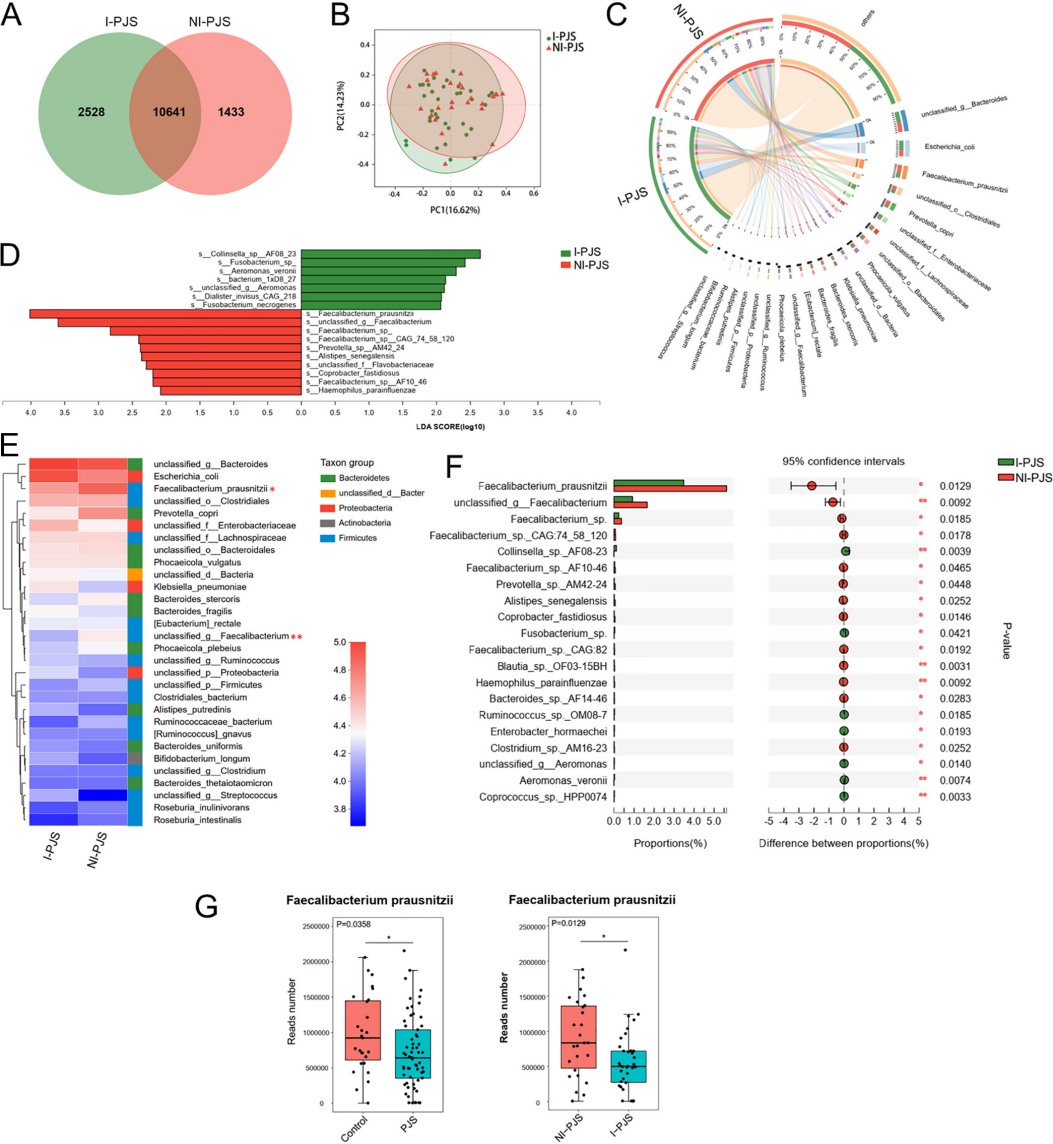
Distinct taxonomic signatures with intussusception based on metagenomic data. (A) Venn diagram displayed the degree of overlap of species between I-PJS and NI-PJS patients. (B) PCoA based on Bray-Curtis distance at the species level. PERMANOVA, *P > *0.05. (C) Circos diagram of dominant species between I-PJS and NI-PJS patients. (D) LEfSe analysis showed the differential species between I-PJS and NI-PJS patients (LDA > 2.0). (E) Heat map depicting the relative abundances of the top 30 species across two groups. (F) Extended error bar plot revealing the top 20 significantly different species between I-PJS and NI-PJS patients. (G) Box plot showing the difference in relative gene abundances of Faecalibacterium prausnitzii. Data were evaluated by the Wilcoxon rank sum test. *, *P < *0.05; **, *P < *0.01; ***, *P < *0.001.

Organisms were classified according to the absence or presence of intussusception in the PJS patients. A Venn diagram shows that there were 1,433 unique species in the NI-PJS group and 2,528 unique species in the I-PJS group, while 10,641 species were shared by the two groups (Fig. 4A). PCoA did not reveal a significantly separate clustering in microbiota structures between groups (Fig. 4B). Species-level comparison of the gut microbiomes showed significant differences in taxonomy (Fig. 4C). LEfSe analysis was used to screen the differential microbes among species (LDA score > 2). Seven species were enriched in the I-PJS group, among which *Collinsella* sp. strain AF08 23, *Fusobacterium*, and Aeromonas veronii were the dominant species. Moreover, 10 species were enriched in the NI-PJS group, among which Faecalibacterium prausnitzii, *unclassified_g_Faecalibacterium*, and *Faecalibacterium* were the dominant species (Fig. 4D). To better understand the differences between two groups, a heat map was constructed of the top 30 species (Fig. 4E). Furthermore, differential abundance analysis showed differences in a total of 459 species between the two groups (Wilcoxon rank sum test [Table S3]). The top 20 differential species are shown in Fig. 4F. In particular, the read number of Faecalibacterium prausnitzii was significantly reduced in PJS patients (*P = *0.0358) and further reduced in patients with intussusception (*P = *0.0129 [Fig. 4G]). Taken together, these analyses indicate that these differential species may play a role in the pathophysiology of PJS.

### Functional profiling of metagenomic data identified disrupted bacterial functions in PJS patients.

**FIG 5:**
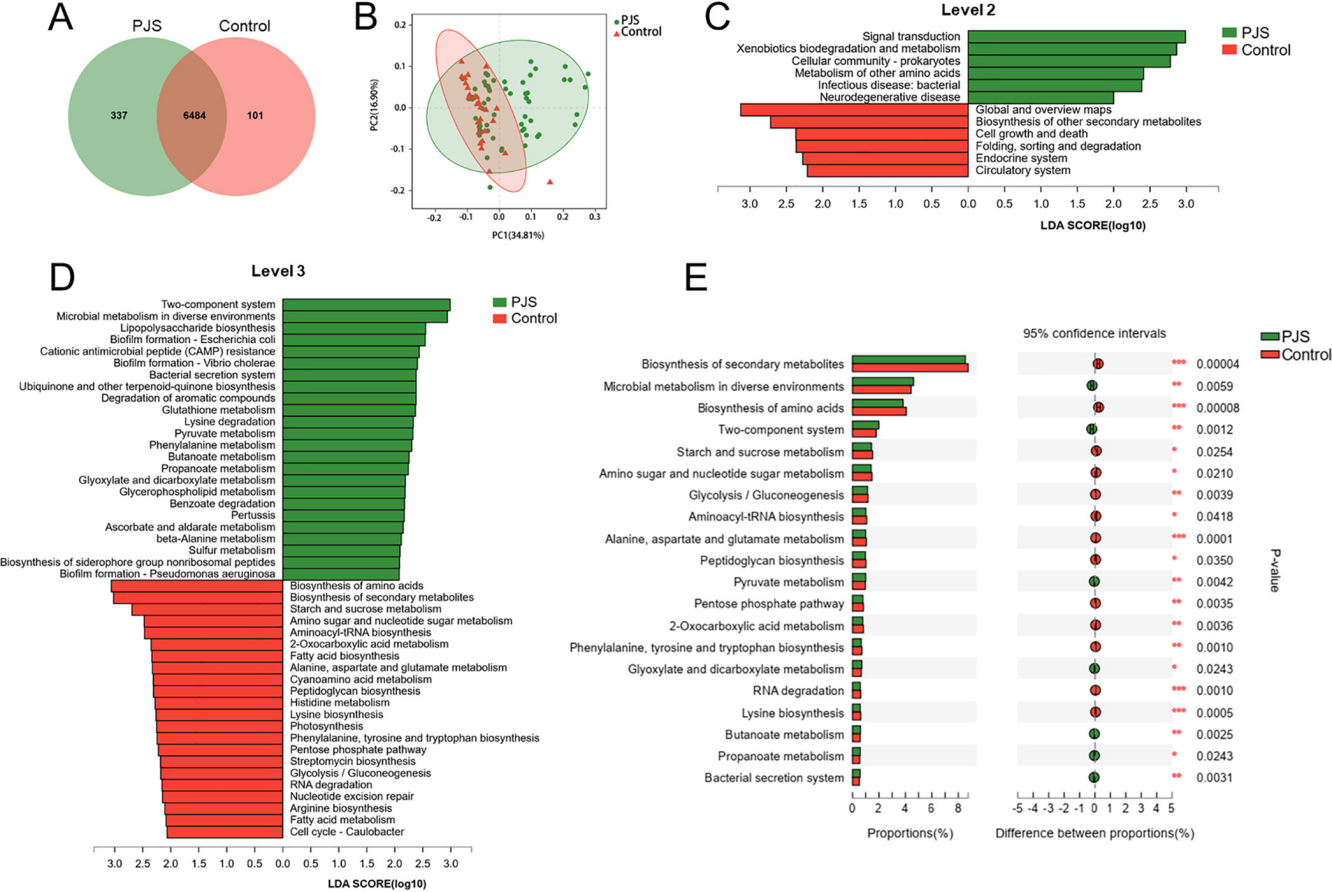
PJS-associated alteration in microbial genes annotated in the KEGG pathway. (A) Venn diagram revealing the shared or unique KO. (B) PCoA based on Bray-Curtis distance of KO between PJS patients and healthy controls. PERMANOVA, *P = *0.001. (C) LEfSe analysis identified the differential enrichment of KEGG pathways (level 2) between PJS patients and healthy controls (LDA > 2.0). (D) LEfSe analysis identified the differential enrichment of KEGG pathways (level 3) between PJS patients and healthy controls (LDA > 2.0). (E) Extended error bar plot showing the top 20 significantly different KEGG pathways (level 3) between PJS patients and healthy controls. Data were evaluated by the Wilcoxon rank sum test. *, *P < *0.05; **, *P < *0.01; ***, *P < *0.001.

In order to gain more insights into the gut microbiome dysbiosis of patients with PJS, we next performed functional analysis of metagenomic sequencing to determine differential genes and pathways between the two groups in our study cohort. A Venn diagram displayed 101 unique KEGG orthologous (KO) in the control group and 337 unique KO in the PJS group, while 6,484 KO were shared by the two groups (Fig. 5A). PCoA, based on KO, demonstrated clustering differences in bacterial functions between two groups (Fig. 5B). Meanwhile, KEGG pathways based on level 2 were disrupted in PJS patients compared to the healthy controls. For instance, the pathways involved in signal transduction, xenobiotics biodegradation and cellular community (prokaryotes) were enriched in the PJS group, while the pathways involved in global and overview maps, biosynthesis of other secondary metabolites, and cell growth and death were enriched in the control group (Fig. 5C). LEfSe analysis further revealed that the three most abundant KEGG pathways (level 3) in the PJS group were two-component system, microbial metabolism in diverse environments, and lipopolysaccharide biosynthesis, while the three most abundant KEGG pathways (level 3) in the control group were biosynthesis of amino acids, biosynthesis of secondary metabolites, and starch and sucrose metabolism (LDA score > 2 [Fig. 5D]). Furthermore, differential abundance analysis showed differences in a total of 134 pathways (level 3) between the two groups (Wilcoxon rank sum test [Table S4]). The top 20 differential pathways (level 3) are shown in Fig. 5E.

**FIG 6:**
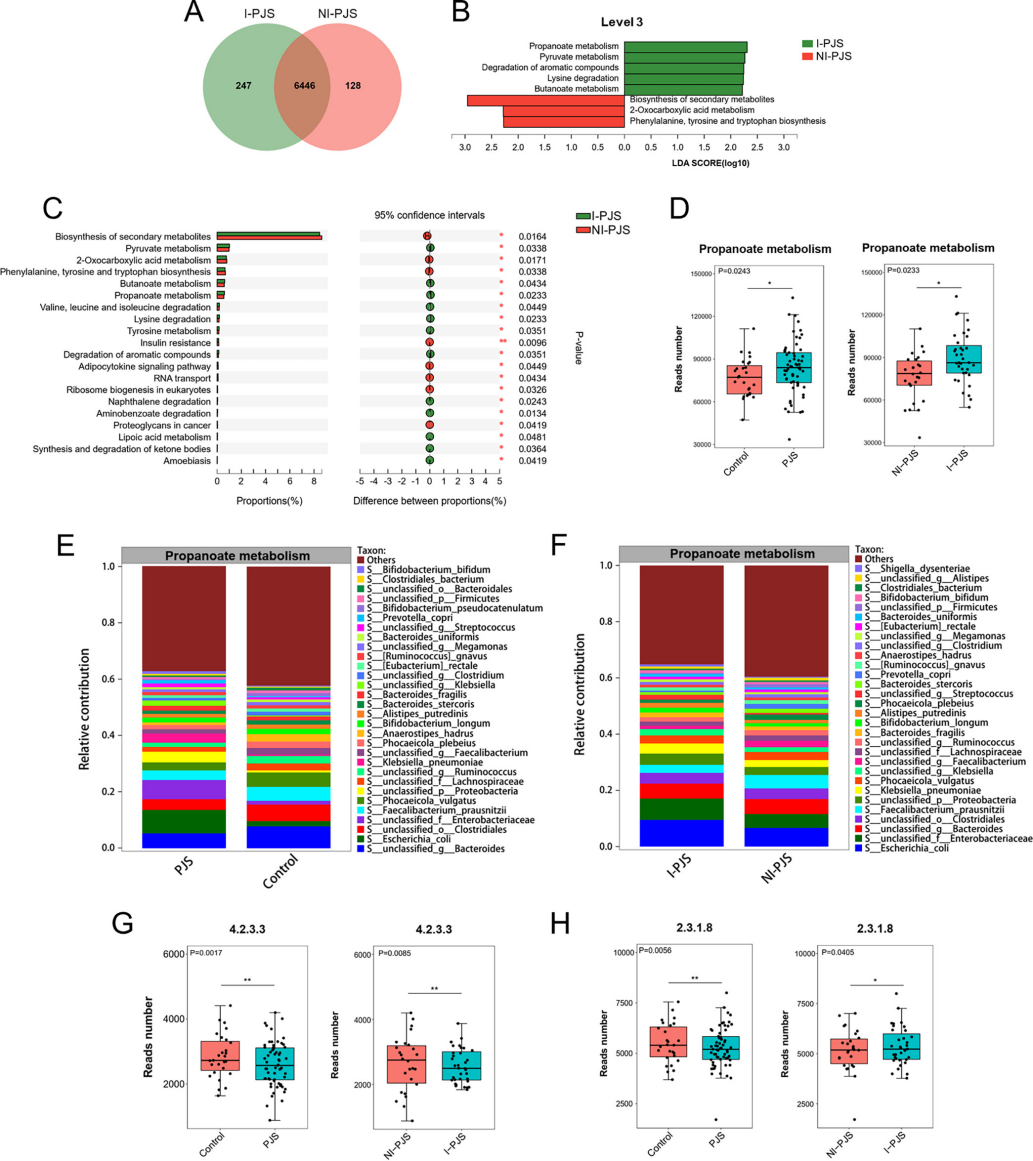
Disrupted functional pattern in PJS patients with intussusception. (A) Venn diagram displaying the distribution of KO between I-PJS and NI-PJS patients. (B) LEfSe analysis showed the differential enrichment of KEGG pathways (level 3) between I-PJS and NI-PJS patients (LDA > 2.0). (C) Extended error bar plot revealing the top 20 significantly different KEGG pathways (level 3) between I-PJS and NI-PJS patients. (D) The differences in relative gene abundances of the propanoate metabolism pathway are shown by box plot. (E and F) Relative contribution of specific bacterial species to the propanoate metabolism pathway. (G and H) The differences in relative gene abundances of EC 4.2.3.3 (*mgsA*) and EC 2.3.1.8 (*pta*) are shown by box plot. Data were evaluated by the Wilcoxon rank sum test. *, *P < *0.05; **, *P < *0.01; ***, *P < *0.001.

The association of microbiome gene pathways with intussusception was further analyzed. A Venn diagram shows that there were 247 unique KO in the I-PJS group and 128 unique KO in the NI-PJS group, while 6,446 KO were shared by the two groups (Fig. 6A). LEfSe analysis was used to screen the differential KEGG pathways (level 3; LDA score > 2). Five pathways (level 3) were enriched in I-PJS group, among which propanoate metabolism was the most dominant pathway. Moreover, 3 pathways (level 3) were enriched in the NI-PJS group, among which biosynthesis of secondary metabolites was the most dominant pathway (Fig. 6B). Furthermore, differential abundance analysis showed differences in a total of 25 pathways (level 3) between the two groups (Wilcoxon rank sum test [Table S5]). The top 20 differential pathways (level 3) are shown in Fig. 6C. In particular, read number for propanoate metabolism was significantly enriched in PJS patients (*P = *0.0243) and further enriched in patients with intussusception (*P = *0.0233 [Fig. 6D and Fig. S4]). Furthermore, we investigated whether specific bacterial species contribute to the propanoate metabolism pathway in our cohort. Escherichia coli was major contributor to the enrichment of the KEGG pathway involved in propanoate metabolism (PJS versus control and I-PJS versus NI-PJS [Fig. 6E and F]). We further measured the abundance of enzymes involved in the propanoate metabolism pathway (Fig. S5 and S6). In particular, read numbers for methylglyoxal synthase (EC 4.2.3.3; encoded by *mgsA*) and phosphate acetyltransferase (EC 2.3.1.8; encoded by *pta*) were significantly decreased in PJS patients (*P = *0.0056 and *P = *0.0017, respectively) and further decreased in patients with intussusception (*P = *0.0405 and *P = *0.0085, respectively [Fig. 6G and H]).

Given the important role of AMPK signaling pathway in the pathogenesis of PJS (22), we further determined the differential abundance of AMPK signaling pathways between the PJS and control groups. We found that AMPK signaling pathway increased in the PJS group relative to the control group (Wilcoxon rank sum test [Fig. S7A]), and Shigella flexneri contributed predominantly to the AMPK signaling pathway in PJS patients (Fig. S7B). Overall, these findings indicated that PJS patients displayed dysfunction in the KEGG pathway, which may play a part in the development of PJS and the occurrence of intussusception.

## DISCUSSION

In the present study, we first discovered that the microbial composition and functional profiling were significantly different between PJS patients and healthy family controls and revealed that alterations in gut microbiome are closely linked with intussusception in PJS patients. Recent research indicates that the gut microbiome contributes to the maintenance of intestinal health and to the etiology of gastrointestinal polyposis and neoplasia. Consequently, there is a need to better identify specific modifications associated with common observations in clinical practice.

PJS is a rare autosomal dominant condition distinguished by mucocutaneous melanin pigmentation and gastrointestinal hamartomatous polyps. We first studied whether the intestinal microbiome was involved in the pathogenesis of PJS. Although the decrease in α-diversity was not statistically significant, the community-level change associated with PJS showed significant differences from the case for healthy family members. Greater person-to-person variability in microbial composition (β-diversity) was also observed in PJS patients. This indicates that even though PJS patients share the same lifestyle, the same dietary habits, and a similar genetic code with their healthy family members, gut microbiome dysbiosis exists in patients with PJS.

PJS patients were enriched in potentially harmful bacteria in their gut, such as pathogenic bacteria and mucin-degrading bacteria (Escherichia-*Shigella*, Klebsiella, *Fusobacterium*, *Veillonella*, and *Akkermansia*), but depleted of commensal bacteria, such as butyrate-producing bacteria and lactate-producing bacteria (*Faecalibacterium*, *Subdoligranulum*, and *Lactobacillus*), at the genus level. These findings were rather in line with those recently reported for CRC patients, indicating that pathogenic bacteria (Escherichia-*Shigella*, Klebsiella, and *Fusobacterium*) and mucin-degrading bacteria (*Akkermansia*) were more abundant in the gut microbiota of CRC patients and that butyrate-producing bacteria (*Lachnospiraceae*) were less abundant (23–25). At the species level, LEfSe analysis showed that a total of 21 species were increased in PJS patients, most of them belonging to the family *Enterobacteriaceae* and the genera Escherichia-*Shigella* and Klebsiella. In particular, Escherichia coli, belonging to the family *Enterobacteriaceae*, was the most abundant bacterial species in PJS patients. An overgrowth of Escherichia coli, considered a tumorigenic bacterium, has been reported to be related to the pathogenesis of CRC (26–28). Additionally, we found that Faecalibacterium prausnitzii showed the most obvious decline in PJS patients, which is consistent with previous findings for adenomatous polyps and CRC (29–31). These results indicate that the structures of the gut microbiome were considerably different between PJS patients and healthy family controls.

It is worth noting that the microbiome and intestinal disorders have intricate interactions. On the one hand, the progression of intestinal disorders modifies microbial profiles; on the other hand, microbiome shifts in certain regions might directly or indirectly induce intestinal disorders. In CRC, gene mutations, especially loss-of-function mutations, disrupt the composition of the gut microbiome (32). Gene mutations can affect the entire microbial network, not just individuals. Additionally, intestinal barrier dysfunction, a prevalent hallmark in gastrointestinal diseases, has been shown to alter the balance between host and microbe in a *Drosophila* tumor model (33). Moreover, microbiotas can thrive and promote immunological and molecular alterations in the microenvironment of a specific disease. For instance, the increased abundance of Fusobacterium nucleatum in CRC influences colorectal carcinogenesis through the production of adhesins and lipopolysaccharide (34). Overall, the microbial community may be impacted by genetic abnormalities and intestinal barrier dysfunction in PJS. And the altered microbiota may contribute to the development of PJS through multiple mechanisms.

Intussusception is the most urgent gastrointestinal complication of PJS patients, and regular gastrointestinal surveillance is of great importance. We further analyzed the gut microbiota composition of PJS patients with or without intussusception. Although the differences in α-diversity and β-diversity were not statistically significant, the dominant taxa between two groups showed the same trend in the PJS patients relative to healthy family members. We found that Escherichia-*Shigella*, *Fusobacterium*, and Klebsiella were also enriched in PJS patients with intussusception (I-PJS), while *Faecalibacterium* was less abundant. At the species level, LEfSe analysis showed that 10 species were increased in PJS patients without intussusception (NI-PJS), most of which belonged to the genus *Faecalibacterium*, and Faecalibacterium prausnitzii was the most dominant species. Characteristic reduction of Faecalibacterium prausnitzii abundance was observed in either PJS patients relative to healthy family controls or I-PJS patients relative to NI-PJS patients.

Faecalibacterium prausnitzii, one of the most prevalent bacteria in the human intestine, promotes the health of the gastrointestinal tract by strengthening the intestinal epithelial barrier function, exerting anti-inflammatory activity, and regulating the mucus pathway (35). Faecalibacterium prausnitzii is extremely responsive to fluctuations in the intestinal microenvironment, and variations in the population richness and quantity of this species have been found in a range of digestive system illnesses (36, 37). Low fecal counts of Faecalibacterium prausnitzii were connected with the progression of hepatitis B virus-related acute-on-chronic liver failure (HBV-ACLF), while the recovery of this species was associated with the remission of HBV-ACLF (38). In addition, a phase III randomized controlled trial recently reported that the abundance Faecalibacterium prausnitzii markedly rebounded after treatment with ursodeoxycholic acid, a therapeutic bile acid, in patients with colorectal adenoma (39). Therefore, Faecalibacterium prausnitzii may serve as a biomarker to assist in the diagnosis and prognosis of intestinal diseases. Overall, these findings imply that the decline in the abundance of Faecalibacterium prausnitzii is connected with the pathogenesis and progression of PJS. Monitoring the dynamic changes of the gut microbiome is expected to become a noninvasive and straightforward method for gastrointestinal surveillance in PJS patients, providing a reference for the prediction of intestinal intussusception.

In addition to taxonomic differences, we found a carbohydrate metabolism pathway involving genes encoding propanoate metabolism that was more abundant in PJS patients than in healthy family controls, and further enhanced in I-PJS patients relative to NI-PJS patients. Propanoate metabolism is downstream of lipid metabolism. Propanoate transforms into succinyl coenzyme A (succinyl-CoA), which then enters the tricarboxylic acid (TCA) cycle in order to generate energy or promote lipogenesis (40). The enhancement of propanoate metabolism may help to maintain the complex communication network among the microbial community in PJS patients via providing more energy. Abnormal propanoate metabolism was reported to be associated with wide range of disorders, such as intrahepatic cholestasis of pregnancy (41), lower-grade glioma (42), colorectal cancer (43), and pancreatic carcinoma (44). Propanoate metabolism can be utilized as a carbon source for fatty acid production and can stimulate tumor growth (44). Moreover, phosphatidylinositol 3-kinase (PI3K) selective molecule repressed tumor angiogenesis and CRC growth via the downregulation of propanoate metabolism and lipogenesis (45). Therefore, the further enhancement of propanoate metabolism in I-PJS patients relative to NI-PJS patients may be associated with a poor prognosis.

Multiple microorganisms were discovered to make a significant contribution to carbohydrate metabolism pathways. In particular, the species Escherichia coli contributed considerably more to the propanoate metabolism in PJS patients and in those with intussusception. Escherichia coli harboring the genomic island polyketide synthase (*pks*^+^) was an essential component of bacterial biofilms found in the intestinal mucosae of patients with familial adenomatous polyposis (FAP) (18). The genotoxin colibactin, produced by *pks*^+^ Escherichia coli, may enhance colorectal tumorigenesis by inducing possibly mutagenic DNA damage (46). Otherwise, the most prevalent virulence genes of Escherichia coli isolated from colonic adenomatous polyps were *fimH* and *fyuA* (47). In order to adapt to the local environmental changes in colonic adenomatous polyps, Escherichia coli expressed specific phenotypic traits, resulting in the improved biofilm formation and poor proteolytic activity (48). The identification of genotypic and phenotypic traits of Escherichia coli may improve the understanding of the molecular mechanisms underlying PJS pathogenesis.

We also found that genes encoding two key enzymes in propanoate metabolism pathway were decreased in PJS patients and further decreased in patients with intussusception. Methylglyoxal synthase (EC 4.2.3.3; encoded by *mgsA*) is a crucial glycolysis bypass protein which produces methylglyoxal from the glycolytic intermediate dihydroxyacetone phosphate (49). A high concentration of methylglyoxal is toxic to cell growth (50). Phosphate acetyltransferase (EC 2.3.1.8; encoded by *pta*) catalyzes the reversible transition of the acetyl group from acetyl-P to CoA, producing acetyl-CoA and inorganic phosphate and contributing to the acetate assimilation or dissimilation process (51). The abnormal expression of *mgsA* and *pta* might be a crucial link in the pathogenesis and progression of PJS. However, the underlying mechanism requires further exploration.

Several limitations in our study should be noted. First, the difference in the ages of the PJS patients and their family members may have an impact on the results of the microbiotas. Second, geographical differences and dietary differences among the various families may affect the results of the microbiotas. Third, the present study was a cross-sectional study which allowed for association but could not address causality. Therefore, a well-designed prospective study and translational studies in animal models should be conducted to confirm our findings.

In summary, the present study demonstrated that PJS patients revealed a unique microbiome composition, characterized by a greater variation in β-diversity, an enhancement of the species Escherichia coli, and a reduction of the species Faecalibacterium prausnitzii. Further reduction of Faecalibacterium prausnitzii was observed in I-PJS patients relative to NI-PJS patients. Functional analysis of the intestinal microbiome identified a greater abundance of propanoate metabolism and abnormal expression of *mgsA* and *pta* in PJS. These findings may provide insight into our understanding of the molecular mechanisms underlying PJS pathogenesis.

## MATERIALS AND METHODS

### Study cohorts.

This was an observational and cross-sectional clinical study of a Chinese family cohort containing 168 PJS patients and 68 healthy family members. Case participants were patients with PJS diagnosed according to the diagnostic criteria as defined by the World Health Organization (2) and/or they were identified as being STK11 gene mutation carriers. Control participants were healthy family members of the selected patients who had lived with the patients for at least 1 year. Demographic and clinical information was obtained from each participant via interviews and questionnaires. The following data were obtained: age, gender, body mass index (BMI), region, gastrointestinal symptoms, mucocutaneous pigmented lesions, gastrointestinal polyps, intussusception or obstruction, history of surgery, family history of PJS, diagnosis, and type of tumor. Individuals who took antibiotics within 3 months prior to recruitment were excluded. Adult participants provided informed consent; minors provided assent and informed consent was obtained from their parents or guardians prior to fecal sample collection. Ethics approval was obtained from the Ethics Committee of Southern Medical University Nanfang Hospital.

### Fecal sample collection and DNA extraction.

Fecal samples were self-collected at home by participants according to the manufacturer’s instructions, delivered immediately to the laboratory at low temperatures, and then stored at –80°C until DNA extraction. DNA extractions from fecal samples were carried out using the E.Z.N.A. stool DNA kit (Omega Bio-tek, Norcross, GA, USA). A NanoDrop 2000 instrument (Thermo Scientific, Wilmington, NC, USA) was used to evaluate the concentration and purity of extracted DNA.

### 16S rRNA gene amplicon sequencing.

PCR amplification was performed using primers (forward, CCTACGGGNGGCWGCAG; reverse, GACTACHVGGGTATCTAATCC) (52) that targeted the V3-V4 region of the bacterial 16S rRNA gene. PCR mixtures (25 μL) contained 12.5 μL of PCR Premix, 5 μL of primer mix (2.5 μL of each primer), 25 ng of template DNA, and RNase-free double-distilled water (ddH_2_O) to adjust the volume. The following cycling was used: initial denaturation at 98°C for 30 s and then 32 cycles of denaturation at 98°C for 10 s, annealing at 54°C for 30 s, and extension at 72°C for 45 s, followed by final extension at 72°C for 10 min. The libraries were sequenced on the Illumina NovaSeq platform according to the manufacturer’s recommendations. Raw reads were demultiplexed, quality filtered by Vsearch (v2.3.4), and merged by FLASH (v1.2.7). α- and β-diversities were calculated with QIIME2, and graphs were drawn with R package. Other charts were generated using R package (v3.5.2).

### Metagenomic sequencing.

Sixty-one PJS patients and 27 healthy family members were picked out by microPITA (53) (https://huttenhower.sph.harvard.edu/micropita) as “representative sampling” to perform metagenomics analysis. Extracted DNA from the selected samples was fragmented to about 400 bp using Covaris M220 (Gene Company Limited, China), and a paired-end library was generated using NEXTflex rapid DNA-Seq (Bioo Scientific, Austin, TX, USA). Metagenomic sequencing was carried out on the Illumina NovaSeq (Illumina, San Diego, CA) platform. The clean reads were generated by removing low-quality reads using fastp v0.20.0 (54) (https://github.com/OpenGene/fastp) and discarding the human host-originated reads (according to the human hg38 reference genome) using BWA v0.7.9a (55) (http://bio-bwa.sourceforge.net). The high-quality reads were then assembled to contigs using MEGAHIT v1.1.2 (56) (https://github.com/voutcn/megahit); lengths of 300 bp or more were selected.

### Gene prediction, taxonomy, and functional annotation.

MetaGene was used to identify the open reading frames (ORFs) in the assembled contigs (57). The predicted ORFs with lengths equal to or more than 100 bp were translated into the corresponding amino acid sequences. CD-HIT software (v4.6.1) was used to remove the redundant sequences (95% identity; 90% overlap) and construct a nonredundant gene catalogue (58) (http://www.bioinformatics.org/cd-hit/). Taxonomic annotations of nonredundant gene catalogue against the entire NCBI NR database were performed using Diamond v0.8.35 (http://github.com/bbuchfink/diamond) (59). The KEGG annotation was conducted using Diamond v0.8.35 against the Kyoto Encyclopedia of Genes and Genomes database (v94.2).

### Bioinformatics and statistical analysis.

Co-occurrence network analysis and random forest model was conducted using the OmicStudio tools (https://www.omicstudio.cn/tool). Receiver operating characteristic (ROC) analysis was performed using the OECloud tools available at https://cloud.oebiotech.cn. Linear discriminant analysis (LDA) effect size (LEfSe) was used to discover differentially abundant taxa or KEGG pathways represented between any two groups. Wilcoxon rank sum test was performed to determine whether there was a significant difference in the abundance of taxa and KEGG pathways between any two groups. Differences were considered statistically significant when the false-discovery rate (FDR)-corrected *P* value was <0.05.

### Data availability.

The raw sequence data have been deposited in the SRA database under accession number PRJNA905444.

## References

[1] Hemminki A. 1999. The molecular basis and clinical aspects of Peutz-Jeghers syndrome. Cell Mol Life Sci 55:735–750. doi:10.1007/s000180050329. 10379360 PMC11146769

[2] Beggs AD, Latchford AR, Vasen HF, Moslein G, Alonso A, Aretz S, Bertario L, Blanco I, Bulow S, Burn J, Capella G, Colas C, Friedl W, Moller P, Hes FJ, Jarvinen H, Mecklin JP, Nagengast FM, Parc Y, Phillips RK, Hyer W, Ponz de Leon M, Renkonen-Sinisalo L, Sampson JR, Stormorken A, Tejpar S, Thomas HJ, Wijnen JT, Clark SK, Hodgson SV. 2010. Peutz-Jeghers syndrome: a systematic review and recommendations for management. Gut 59:975–986. doi:10.1136/gut.2009.198499. 20581245

[3] Tacheci I, Kopacova M, Bures J. 2021. Peutz-Jeghers syndrome. Curr Opin Gastroenterol 37:245–254. doi:10.1097/MOG.0000000000000718. 33591027

[4] Wagner A, Aretz S, Auranen A, Bruno MJ, Cavestro GM, Crosbie EJ, Goverde A, Jelsig AM, Latchford A, Leerdam MEV, Lepisto A, Puzzono M, Winship I, Zuber V, Moslein G. 2021. The management of Peutz-Jeghers syndrome: European Hereditary Tumour Group (EHTG) guideline. J Clin Med 10:473. doi:10.3390/jcm10030473. 33513864 PMC7865862

[5] Wang H, Luo T, Liu WQ, Huang Y, Wu XT, Wang XJ. 2011. Clinical presentations and surgical approach of acute intussusception caused by Peutz-Jeghers syndrome in adults. J Gastrointest Surg 15:2218–2225. doi:10.1007/s11605-011-1724-2. 22005897

[6] van Lier MG, Mathus-Vliegen EM, Wagner A, van Leerdam ME, Kuipers EJ. 2011. High cumulative risk of intussusception in patients with Peutz-Jeghers syndrome: time to update surveillance guidelines? Am J Gastroenterol 106:940–945. doi:10.1038/ajg.2010.473. 21157440

[7] Latchford A, Cohen S, Auth M, Scaillon M, Viala J, Daniels R, Talbotec C, Attard T, Durno C, Hyer W. 2019. Management of Peutz-Jeghers syndrome in children and adolescents: a position paper from the ESPGHAN Polyposis Working Group. J Pediatr Gastroenterol Nutr 68:442–452. doi:10.1097/MPG.0000000000002248. 30585892

[8] Latchford AR, Neale K, Phillips RK, Clark SK. 2011. Peutz-Jeghers syndrome: intriguing suggestion of gastrointestinal cancer prevention from surveillance. Dis Colon Rectum 54:1547–1551. doi:10.1097/DCR.0b013e318233a11f. 22067184

[9] van Lier MG, Wagner A, Mathus-Vliegen EM, Kuipers EJ, Steyerberg EW, van Leerdam ME. 2010. High cancer risk in Peutz-Jeghers syndrome: a systematic review and surveillance recommendations. Am J Gastroenterol 105:1258–1264. doi:10.1038/ajg.2009.725. 20051941

[10] Ollila S, Domenech-Moreno E, Laajanen K, Wong IP, Tripathi S, Pentinmikko N, Gao Y, Yan Y, Niemela EH, Wang TC, Viollet B, Leone G, Katajisto P, Vaahtomeri K, Makela TP. 2018. Stromal Lkb1 deficiency leads to gastrointestinal tumorigenesis involving the IL-11-JAK/STAT3 pathway. J Clin Invest 128:402–414. doi:10.1172/jci93597. 29202476 PMC5749537

[11] Kottakis F, Nicolay BN, Roumane A, Karnik R, Gu H, Nagle JM, Boukhali M, Hayward MC, Li YY, Chen T, Liesa M, Hammerman PS, Wong KK, Hayes DN, Shirihai OS, Dyson NJ, Haas W, Meissner A, Bardeesy N. 2016. LKB1 loss links serine metabolism to DNA methylation and tumorigenesis. Nature 539:390–395. doi:10.1038/nature20132. 27799657 PMC5988435

[12] Canto C, Auwerx J. 2010. AMP-activated protein kinase and its downstream transcriptional pathways. Cell Mol Life Sci 67:3407–3423. doi:10.1007/s00018-010-0454-z. 20640476 PMC3622821

[13] Li L, Yao Y, Zhao J, Cao J, Ma H. 2020. Dehydroepiandrosterone protects against hepatic glycolipid metabolic disorder and insulin resistance induced by high fat via activation of AMPK-PGC-1alpha-NRF-1 and IRS1-AKT-GLUT2 signaling pathways. Int J Obes (Lond) 44:1075–1086. doi:10.1038/s41366-019-0508-8. 31911660

[14] Kang X, Zhang R, Kwong TN, Lui RN, Wu WK, Sung JJ, Yu J, Wong SH. 2021. Serrated neoplasia in the colorectum: gut microbiota and molecular pathways. Gut Microbes 13:1863135. doi:10.1080/19490976.2020.1863135. 33382354 PMC7781617

[15] Mori G, Pasca MR. 2021. Gut microbial signatures in sporadic and hereditary colorectal cancer. Int J Mol Sci 22:1312. doi:10.3390/ijms22031312. 33525662 PMC7865401

[16] Pop OL, Vodnar DC, Diaconeasa Z, Istrati M, Bintintan A, Bintintan VV, Suharoschi R, Gabbianelli R. 2020. An overview of gut microbiota and colon diseases with a focus on adenomatous colon polyps. Int J Mol Sci 21:7359. doi:10.3390/ijms21197359. 33028024 PMC7582333

[17] Song M, Nguyen LH, Emilsson L, Chan AT, Ludvigsson JF. 2021. Antibiotic use associated with risk of colorectal polyps in a nationwide study. Clin Gastroenterol Hepatol 19:1426–1435.e6. doi:10.1016/j.cgh.2020.05.036. 32454258 PMC9727504

[18] Dejea CM, Fathi P, Craig JM, Boleij A, Taddese R, Geis AL, Wu X, DeStefano Shields CE, Hechenbleikner EM, Huso DL, Anders RA, Giardiello FM, Wick EC, Wang H, Wu S, Pardoll DM, Housseau F, Sears CL. 2018. Patients with familial adenomatous polyposis harbor colonic biofilms containing tumorigenic bacteria. Science 359:592–597. doi:10.1126/science.aah3648. 29420293 PMC5881113

[19] Hale VL, Chen J, Johnson S, Harrington SC, Yab TC, Smyrk TC, Nelson H, Boardman LA, Druliner BR, Levin TR, Rex DK, Ahnen DJ, Lance P, Ahlquist DA, Chia N. 2017. Shifts in the fecal microbiota associated with adenomatous polyps. Cancer Epidemiol Biomarkers Prev 26:85–94. doi:10.1158/1055-9965.EPI-16-0337. 27672054 PMC5225053

[20] Peters BA, Dominianni C, Shapiro JA, Church TR, Wu J, Miller G, Yuen E, Freiman H, Lustbader I, Salik J, Friedlander C, Hayes RB, Ahn J. 2016. The gut microbiota in conventional and serrated precursors of colorectal cancer. Microbiome 4:69. doi:10.1186/s40168-016-0218-6. 28038683 PMC5203720

[21] Yachida S, Mizutani S, Shiroma H, Shiba S, Nakajima T, Sakamoto T, Watanabe H, Masuda K, Nishimoto Y, Kubo M, Hosoda F, Rokutan H, Matsumoto M, Takamaru H, Yamada M, Matsuda T, Iwasaki M, Yamaji T, Yachida T, Soga T, Kurokawa K, Toyoda A, Ogura Y, Hayashi T, Hatakeyama M, Nakagama H, Saito Y, Fukuda S, Shibata T, Yamada T. 2019. Metagenomic and metabolomic analyses reveal distinct stage-specific phenotypes of the gut microbiota in colorectal cancer. Nat Med 25:968–976. doi:10.1038/s41591-019-0458-7. 31171880

[22] Zhang Y, Meng Q, Sun Q, Xu ZX, Zhou H, Wang Y. 2021. LKB1 deficiency-induced metabolic reprogramming in tumorigenesis and non-neoplastic diseases. Mol Metab 44:101131. doi:10.1016/j.molmet.2020.101131. 33278637 PMC7753952

[23] Wang T, Cai G, Qiu Y, Fei N, Zhang M, Pang X, Jia W, Cai S, Zhao L. 2012. Structural segregation of gut microbiota between colorectal cancer patients and healthy volunteers. ISME J 6:320–329. doi:10.1038/ismej.2011.109. 21850056 PMC3260502

[24] Han S, Pan Y, Yang X, Da M, Wei Q, Gao Y, Qi Q, Ru L. 2019. Intestinal microorganisms involved in colorectal cancer complicated with dyslipidosis. Cancer Biol Ther 20:81–89. doi:10.1080/15384047.2018.1507255. 30239257 PMC6343685

[25] Weir TL, Manter DK, Sheflin AM, Barnett BA, Heuberger AL, Ryan EP. 2013. Stool microbiome and metabolome differences between colorectal cancer patients and healthy adults. PLoS One 8:e70803. doi:10.1371/journal.pone.0070803. 23940645 PMC3735522

[26] Gagniere J, Raisch J, Veziant J, Barnich N, Bonnet R, Buc E, Bringer MA, Pezet D, Bonnet M. 2016. Gut microbiota imbalance and colorectal cancer. World J Gastroenterol 22:501–518. doi:10.3748/wjg.v22.i2.501. 26811603 PMC4716055

[27] Khan AA, Khan Z, Malik A, Kalam MA, Cash P, Ashraf MT, Alshamsan A. 2017. Colorectal cancer-inflammatory bowel disease nexus and felony of Escherichia coli. Life Sci 180:60–67. doi:10.1016/j.lfs.2017.05.016. 28506682

[28] Cougnoux A, Dalmasso G, Martinez R, Buc E, Delmas J, Gibold L, Sauvanet P, Darcha C, Dechelotte P, Bonnet M, Pezet D, Wodrich H, Darfeuille-Michaud A, Bonnet R. 2014. Bacterial genotoxin colibactin promotes colon tumour growth by inducing a senescence-associated secretory phenotype. Gut 63:1932–1942. doi:10.1136/gutjnl-2013-305257. 24658599

[29] Liang S, Mao Y, Liao M, Xu Y, Chen Y, Huang X, Wei C, Wu C, Wang Q, Pan X, Tang W. 2020. Gut microbiome associated with APC gene mutation in patients with intestinal adenomatous polyps. Int J Biol Sci 16:135–146. doi:10.7150/ijbs.37399. 31892851 PMC6930378

[30] He T, Cheng X, Xing C. 2021. The gut microbial diversity of colon cancer patients and the clinical significance. Bioengineered 12:7046–7060. doi:10.1080/21655979.2021.1972077. 34551683 PMC8806656

[31] Mangifesta M, Mancabelli L, Milani C, Gaiani F, de’Angelis N, de’Angelis GL, van Sinderen D, Ventura M, Turroni F. 2018. Mucosal microbiota of intestinal polyps reveals putative biomarkers of colorectal cancer. Sci Rep 8:13974. doi:10.1038/s41598-018-32413-2. 30228361 PMC6143603

[32] Burns MB, Montassier E, Abrahante J, Priya S, Niccum DE, Khoruts A, Starr TK, Knights D, Blekhman R. 2018. Colorectal cancer mutational profiles correlate with defined microbial communities in the tumor microenvironment. PLoS Genet 14:e1007376. doi:10.1371/journal.pgen.1007376. 29924794 PMC6028121

[33] Zhou J, Boutros M. 2020. JNK-dependent intestinal barrier failure disrupts host-microbe homeostasis during tumorigenesis. Proc Natl Acad Sci USA 117:9401–9412. doi:10.1073/pnas.1913976117. 32277031 PMC7196803

[34] Garrett WS. 2019. The gut microbiota and colon cancer. Science 364:1133–1135. doi:10.1126/science.aaw2367. 31221845

[35] Miquel S, Martin R, Rossi O, Bermudez-Humaran LG, Chatel JM, Sokol H, Thomas M, Wells JM, Langella P. 2013. Faecalibacterium prausnitzii and human intestinal health. Curr Opin Microbiol 16:255–261. doi:10.1016/j.mib.2013.06.003. 23831042

[36] Lopez-Siles M, Martinez-Medina M, Abella C, Busquets D, Sabat-Mir M, Duncan SH, Aldeguer X, Flint HJ, Garcia-Gil LJ. 2015. Mucosa-associated Faecalibacterium prausnitzii phylotype richness is reduced in patients with inflammatory bowel disease. Appl Environ Microbiol 81:7582–7592. doi:10.1128/AEM.02006-15. 26296733 PMC4592880

[37] De Palma G, Nadal I, Medina M, Donat E, Ribes-Koninckx C, Calabuig M, Sanz Y. 2010. Intestinal dysbiosis and reduced immunoglobulin-coated bacteria associated with coeliac disease in children. BMC Microbiol 10:63. doi:10.1186/1471-2180-10-63. 20181275 PMC2843610

[38] Wang K, Zhang Z, Mo ZS, Yang XH, Lin BL, Peng L, Xu Y, Lei CY, Zhuang XD, Lu L, Yang RF, Chen T, Gao ZL. 2021. Gut microbiota as prognosis markers for patients with HBV-related acute-on-chronic liver failure. Gut Microbes 13:1921925. doi:10.1080/19490976.2021.1921925. 34006193 PMC8143260

[39] Pearson T, Caporaso JG, Yellowhair M, Bokulich NA, Padi M, Roe DJ, Wertheim BC, Linhart M, Martinez JA, Bilagody C, Hornstra H, Alberts DS, Lance P, Thompson PA. 2019. Effects of ursodeoxycholic acid on the gut microbiome and colorectal adenoma development. Cancer Med 8:617–628. doi:10.1002/cam4.1965. 30652422 PMC6382922

[40] MacFabe DF, Cain DP, Rodriguez-Capote K, Franklin AE, Hoffman JE, Boon F, Taylor AR, Kavaliers M, Ossenkopp KP. 2007. Neurobiological effects of intraventricular propionic acid in rats: possible role of short chain fatty acids on the pathogenesis and characteristics of autism spectrum disorders. Behav Brain Res 176:149–169. doi:10.1016/j.bbr.2006.07.025. 16950524

[41] Zhan Q, Qi X, Weng R, Xi F, Chen Y, Wang Y, Hu W, Zhao B, Luo Q. 2021. Alterations of the human gut microbiota in intrahepatic cholestasis of pregnancy. Front Cell Infect Microbiol 11:635680. doi:10.3389/fcimb.2021.635680. 33996622 PMC8120235

[42] Huang R, Li Z, Zhu X, Yan P, Song D, Yin H, Hu P, Lin R, Wu S, Meng T, Zhang J, Huang Z. 2020. Collagen type III alpha 1 chain regulated by GATA-binding protein 6 affects type II IFN response and propanoate metabolism in the recurrence of lower grade glioma. J Cell Mol Med 24:10803–10815. doi:10.1111/jcmm.15705. 32757451 PMC7521258

[43] Zhang HL, Zhang AH, Zhou XH, Sun H, Wang XQ, Liang L, Wang XJ. 2018. High-throughput lipidomics reveal mirabilite regulating lipid metabolism as anticancer therapeutics. RSC Adv 8:35600–35610. doi:10.1039/c8ra06190d. 35547938 PMC9087915

[44] Lee JH, Cho YR, Kim JH, Kim J, Nam HY, Kim SW, Son J. 2019. Branched-chain amino acids sustain pancreatic cancer growth by regulating lipid metabolism. Exp Mol Med 51:1–11. doi:10.1038/s12276-019-0350-z. PMC6884453 31784505

[45] Hussain A, Qazi AK, Mupparapu N, Guru SK, Kumar A, Sharma PR, Singh SK, Singh P, Dar MJ, Bharate SB, Zargar MA, Ahmed QN, Bhushan S, Vishwakarma RA, Hamid A. 2016. Modulation of glycolysis and lipogenesis by novel PI3K selective molecule represses tumor angiogenesis and decreases colorectal cancer growth. Cancer Lett 374:250–260. doi:10.1016/j.canlet.2016.02.030. 26921131

[46] Wilson MR, Jiang Y, Villalta PW, Stornetta A, Boudreau PD, Carra A, Brennan CA, Chun E, Ngo L, Samson LD, Engelward BP, Garrett WS, Balbo S, Balskus EP. 2019. The human gut bacterial genotoxin colibactin alkylates DNA. Science 363:eaar7785. doi:10.1126/science.aar7785. 30765538 PMC6407708

[47] Sarshar M, Scribano D, Marazzato M, Ambrosi C, Aprea MR, Aleandri M, Pronio A, Longhi C, Nicoletti M, Zagaglia C, Palamara AT, Conte MP. 2017. Genetic diversity, phylogroup distribution and virulence gene profile of pks positive Escherichia coli colonizing human intestinal polyps. Microb Pathog 112:274–278. doi:10.1016/j.micpath.2017.10.009. 28987619

[48] Ambrosi C, Sarshar M, Aprea MR, Pompilio A, Di Bonaventura G, Strati F, Pronio A, Nicoletti M, Zagaglia C, Palamara AT, Scribano D. 2019. Colonic adenoma-associated Escherichia coli express specific phenotypes. Microbes Infect 21:305–312. doi:10.1016/j.micinf.2019.02.001. 30763764

[49] Dickmanns A, Zschiedrich CP, Arens J, Parfentev I, Gundlach J, Hofele R, Neumann P, Urlaub H, Gorke B, Ficner R, Stulke J. 2018. Structural basis for the regulatory interaction of the methylglyoxal synthase MgsA with the carbon flux regulator Crh in Bacillus subtilis. J Biol Chem 293:5781–5792. doi:10.1074/jbc.RA117.001289. 29514981 PMC5912461

[50] Totemeyer S, Booth NA, Nichols WW, Dunbar B, Booth IR. 1998. From famine to feast: the role of methylglyoxal production in Escherichia coli. Mol Microbiol 27:553–562. doi:10.1046/j.1365-2958.1998.00700.x. 9489667

[51] Dimou M, Venieraki A, Liakopoulos G, Katinakis P. 2011. Cloning, characterization and transcriptional analysis of two phosphate acetyltransferase isoforms from Azotobacter vinelandii. Mol Biol Rep 38:3653–3663. doi:10.1007/s11033-010-0478-3. 21104132

[52] Logue JB, Stedmon CA, Kellerman AM, Nielsen NJ, Andersson AF, Laudon H, Lindstrom ES, Kritzberg ES. 2016. Experimental insights into the importance of aquatic bacterial community composition to the degradation of dissolved organic matter. ISME J 10:533–545. doi:10.1038/ismej.2015.131. 26296065 PMC4817675

[53] Tickle TL, Segata N, Waldron L, Weingart U, Huttenhower C. 2013. Two-stage microbial community experimental design. ISME J 7:2330–2339. doi:10.1038/ismej.2013.139. 23949665 PMC3834858

[54] Chen S, Zhou Y, Chen Y, Gu J. 2018. fastp: an ultra-fast all-in-one FASTQ preprocessor. Bioinformatics 34:i884–i890. doi:10.1093/bioinformatics/bty560. 30423086 PMC6129281

[55] Li H, Durbin R. 2009. Fast and accurate short read alignment with Burrows-Wheeler transform. Bioinformatics 25:1754–1760. doi:10.1093/bioinformatics/btp324. 19451168 PMC2705234

[56] Li D, Liu CM, Luo R, Sadakane K, Lam TW. 2015. MEGAHIT: an ultra-fast single-node solution for large and complex metagenomics assembly via succinct de Bruijn graph. Bioinformatics 31:1674–1676. doi:10.1093/bioinformatics/btv033. 25609793

[57] Noguchi H, Park J, Takagi T. 2006. MetaGene: prokaryotic gene finding from environmental genome shotgun sequences. Nucleic Acids Res 34:5623–5630. doi:10.1093/nar/gkl723. 17028096 PMC1636498

[58] Fu L, Niu B, Zhu Z, Wu S, Li W. 2012. CD-HIT: accelerated for clustering the next-generation sequencing data. Bioinformatics 28:3150–3152. doi:10.1093/bioinformatics/bts565. 23060610 PMC3516142

[59] Buchfink B, Xie C, Huson DH. 2015. Fast and sensitive protein alignment using DIAMOND. Nat Methods 12:59–60. doi:10.1038/nmeth.3176. 25402007

