# Supplementary material for: Changes in the Gut Microbiome Associated with Intussusception in Patients with Peutz-Jeghers Syndrome
Source: Microbiol Spectr. 2023 Jan 31;11(2):e02819-22. doi: 10.1128/spectrum.02819-22 (PMC10101062; doi:10.1128/spectrum.02819-22)
Supplement: Supplemental file 1 — Supplemental material. Download spectrum.02819-22-s0001.pdf, PDF file, 1.3 MB [file spectrum.02819-22-s0001.pdf]

Figure S1. Taxonomic changes between PJS patients and healthy family controls.
(A) Rarefaction curves generated from the observed Operational Taxonomic Units (OTUs). (B) Histogram of dominant taxa abundance at the phylum level. (C) Histogram of dominant taxa abundance at the genus level.

Figure S2. Taxonomic changes in PJS patients with intussusception.
(A) Histogram of dominant taxa abundance at the phylum level. (B) The differential phyla are shown between I-PJS and NI-PJS. (C) Histogram of dominant taxa abundance at the genus level. (D) The differential genera are shown between I-PJS and NI-PJS. (E) Random forest analysis showed the 20 most discriminant genera distinguishing I-PJS and NI-PJS.

Figure S3. The information of non-redundant gene catalog according to metagenomic data.
(A) Sequence length distribution of non-redundant gene catalog.

Figure S4. Schematic illustrating the Propanoate metabolism pathway that was disturbed in PJS patients with intussusception.

Figure S5. Relative abundance of enzymes involved in the Propanoate metabolism pathway between PJS and healthy controls.

Figure S6. Relative abundance of enzymes involved in the Propanoate metabolism pathway between I-PJS and NI-PJS.

Figure S7. Difference in AMPK signaling pathway between PJS patients and healthy family members.
(A) Extended error bar plot showed the difference in AMPK signaling pathway between PJS patients and healthy controls. (B) Relative contribution of specific bacterial species to AMPK signaling pathway. Data were evaluated by the Wilcoxon rank-sum test. **, $P < 0.01$.

Figure S1

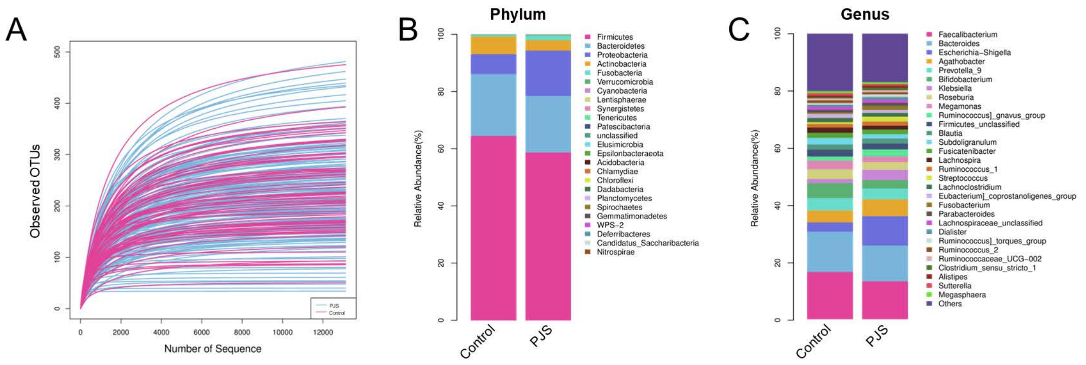
A
B
C
Observed OTUs
Number of Sequence
PJS
Control
Phylum
Relative Abundance(%)
Firmicutes
Bacteroidetes
Proteobacteria
Actinobacteria
Fusobacteria
Verrucomicrobia
Cyanobacteria
Lentisphaerae
Synergistetes
Tenericutes
Patescibacteria
unclassified
Elusimicrobia
Epsilonbacteraeota
Acidobacteria
Chlamydiae
Chloroflexi
Dadabacteria
Planctomycetes
Spirochaetes
Gemmatimonadetes
WPS-2
Deferribacteres
Candidatus_Saccharibacteria
Nitrospirae
Control
PJS
Genus
Relative Abundance(%)
Faecalibacterium
Bacteroides
Escherichia-Shigella
Agathobacter
Prevotella_9
Bifidobacterium
Klebsiella
Roseburia
Megamonas
Ruminococcus]_gnavus_group
Firmicutes_unclassified
Blautia
Subdoligranulum
Fusicatenibacter
Lachnospira
Ruminococcus_1
Streptococcus
Lachnoclostridium
Eubacterium]_coprostanoligenes_group
Fusobacterium
Parabacteroides
Lachnospiraceae_unclassified
Dialister
Ruminococcus]_torques_group
Ruminococcus_2
Ruminococcaceae_UCG-002
Clostridium_sensu_stricto_1
Alistipes
Sutterella
Megasphaera
Others
Control
PJS

Figure S2

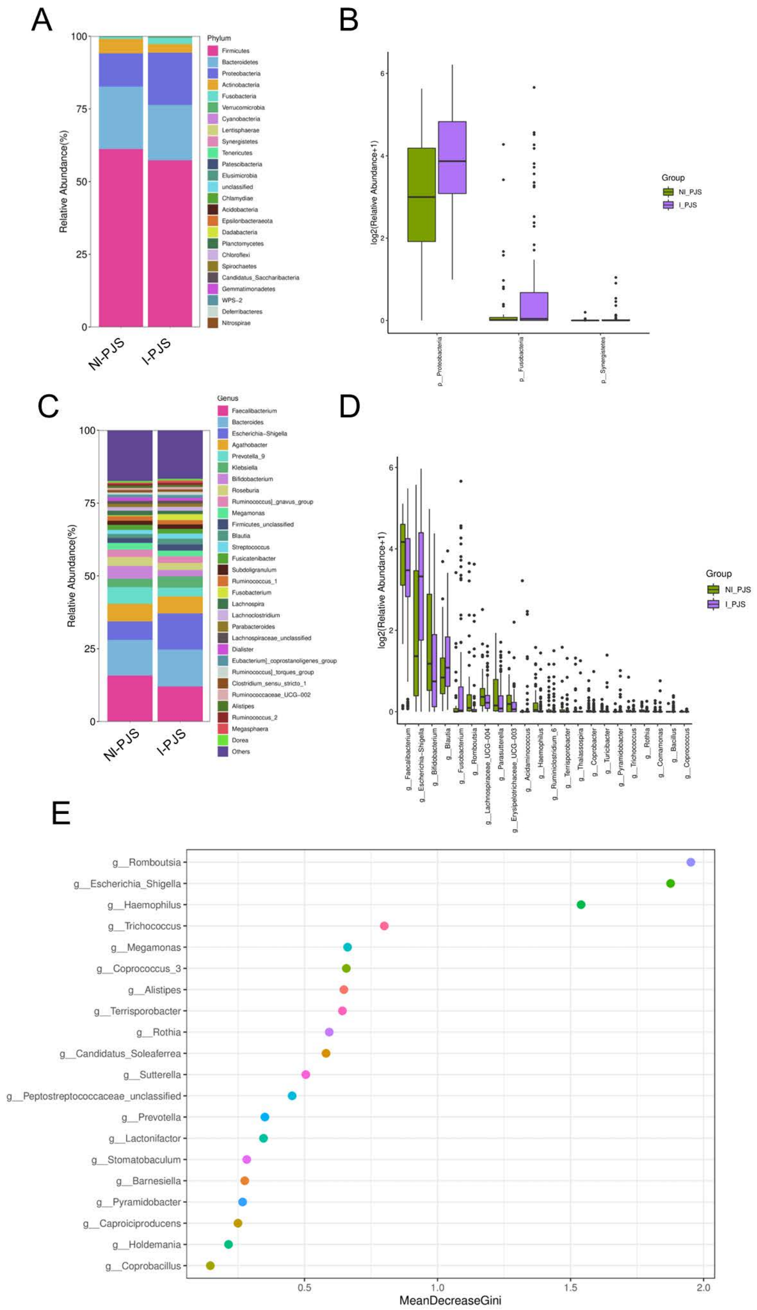

Figure S3

A

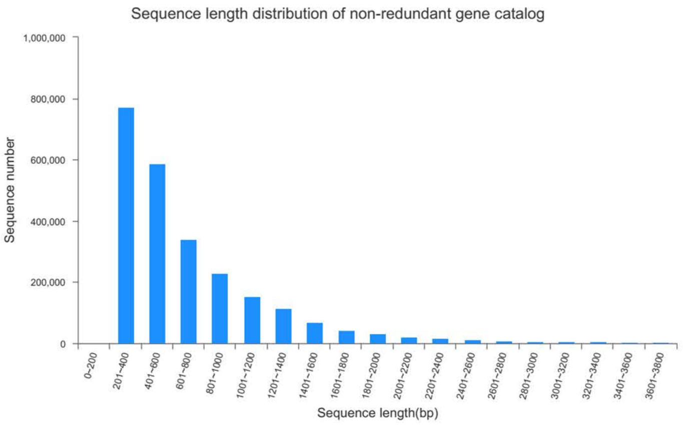
Sequence length distribution of non-redundant gene catalog
1,000,000
800,000
600,000
400,000
200,000
0
Sequence number
0~200
201~400
401~600
601~800
801~1000
1001~1200
1201~1400
1401~1600
1601~1800
1801~2000
2001~2200
2201~2400
2401~2600
2601~2800
2801~3000
3001~3200
3201~3400
3401~3600
3601~3800
Sequence length(bp)

Figure S4

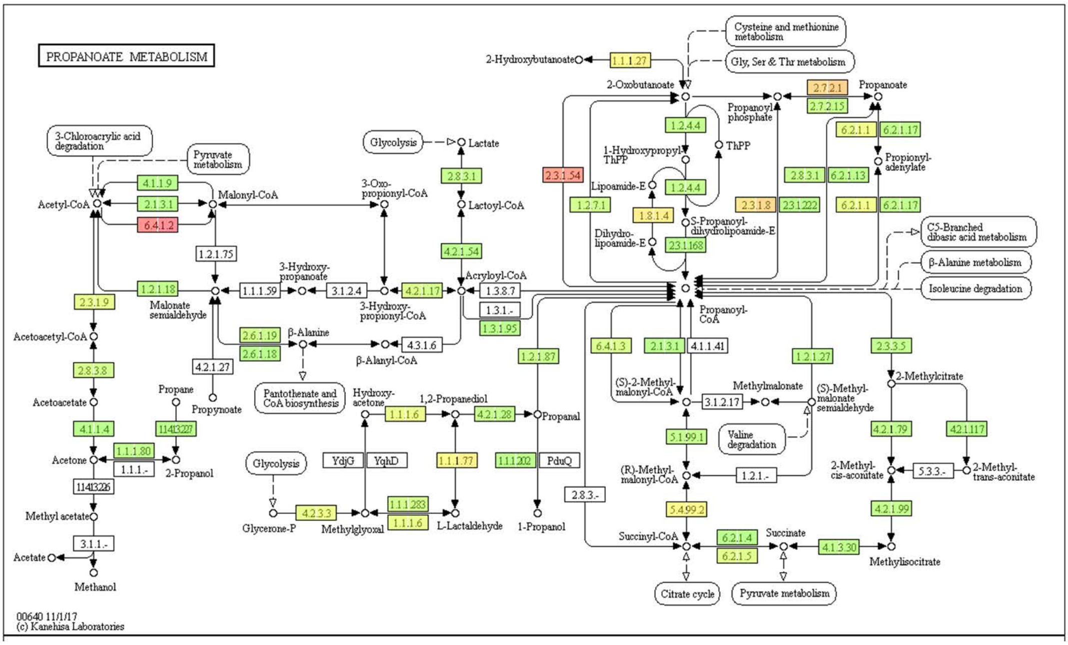
PROPANOATE METABOLISM
2-Hydroxybutanoate
1.1.1.27
Cysteine and methionine metabolism
Gly, Ser & Thr metabolism
2-Oxobutanoate
Propanoyl phosphate
2.7.2.1
2.7.2.15
Propanoate
1.2.4.4
ThPP
1-Hydroxypropyl-ThPP
6.2.1.1
6.2.1.17
Propionyl-adenylate
2.3.1.54
Lipoamide-E
1.2.4.4
2.8.3.1
6.2.1.13
1.2.7.1
2.3.1.8
2.3.1.222
6.2.1.1
6.2.1.17
1.8.1.4
S-Propanoyl-dihydrolipoamide-E
Dihydro-lipoamide-E
2.3.1.168
C5-Branched dibasic acid metabolism
β-Alanine metabolism
Isoleucine degradation
Propanoyl-CoA
3-Chloroacrylic acid degradation
Pyruvate metabolism
Glycolysis
Lactate
2.8.3.1
Lactoyl-CoA
4.1.1.9
2.1.3.1
6.4.1.2
Acetyl-CoA
Malonyl-CoA
3-Oxo-propionyl-CoA
4.2.1.54
1.2.1.75
3-Hydroxy-propanoate
Acryloyl-CoA
1.2.1.18
1.1.1.59
3.1.2.4
4.2.1.17
1.3.8.7
1.3.1.-
1.3.1.95
2.3.1.9
Malonate semialdehyde
3-Hydroxy-propionyl-CoA
Acetoacetyl-CoA
2.6.1.19
2.6.1.18
β-Alanine
4.3.1.6
β-Alanyl-CoA
1.2.1.87
6.4.1.3
2.1.3.1
4.1.1.41
1.2.1.27
2.3.3.5
2.8.3.8
4.2.1.27
Pantothenate and CoA biosynthesis
Hydroxy-acetone
1,2-Propanediol
(S)-2-Methyl-malonyl-CoA
Methylmalonate
(S)-Methyl-malonate semialdehyde
2-Methylcitrate
Acetoacetate
Propane
Propynoate
1.1.1.6
4.2.1.28
Propanal
3.1.2.17
4.1.1.4
1.14.13.227
Valine degradation
5.1.99.1
4.2.1.79
4.2.1.117
1.1.1.80
Acetone
1.1.1.-
2-Propanol
Glycolysis
YdjG
YqhD
1.1.1.77
1.1.1.202
PduQ
(R)-Methyl-malonyl-CoA
1.2.1.-
2-Methyl-cis-aconitate
5.3.3.-
2-Methyl-trans-aconitate
1.14.13.226
2.8.3.-
Methyl acetate
1.1.1.283
1.1.1.6
5.4.99.2
4.2.1.99
Glycerone-P
4.2.3.3
Methylglyoxal
L-Lactaldehyde
1-Propanol
Succinyl-CoA
6.2.1.4
6.2.1.5
Succinate
4.1.3.30
Methylisocitrate
3.1.1.-
Acetate
Methanol
Citrate cycle
Pyruvate metabolism
00640 11/1/17
(c) Kanehisa Laboratories

Figure S5

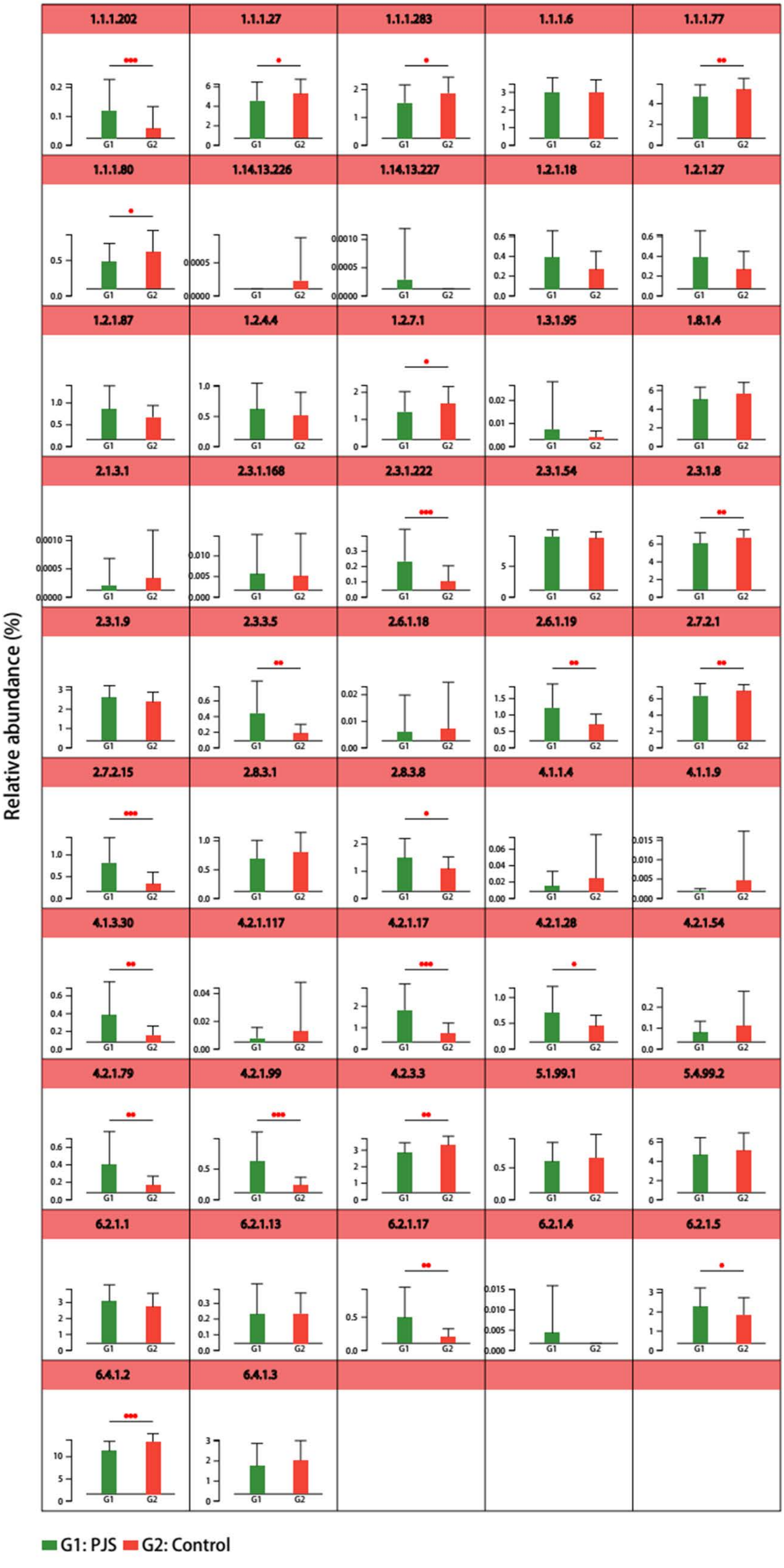
1.1.1.202
1.1.1.27
1.1.1.283
1.1.1.6
1.1.1.77
1.1.1.80
1.14.13.226
1.14.13.227
1.2.1.18
1.2.1.27
1.2.1.87
1.2.4.4
1.2.7.1
1.3.1.95
1.8.1.4
2.1.3.1
2.3.1.168
2.3.1.222
2.3.1.54
2.3.1.8
2.3.1.9
2.3.3.5
2.6.1.18
2.6.1.19
2.7.2.1
2.7.2.15
2.8.3.1
2.8.3.8
4.1.1.4
4.1.1.9
4.1.3.30
4.2.1.117
4.2.1.17
4.2.1.28
4.2.1.54
4.2.1.79
4.2.1.99
4.2.3.3
5.1.99.1
5.4.99.2
6.2.1.1
6.2.1.13
6.2.1.17
6.2.1.4
6.2.1.5
6.4.1.2
6.4.1.3
Relative abundance (%)
G1: PJS
G2: Control

Figure S6

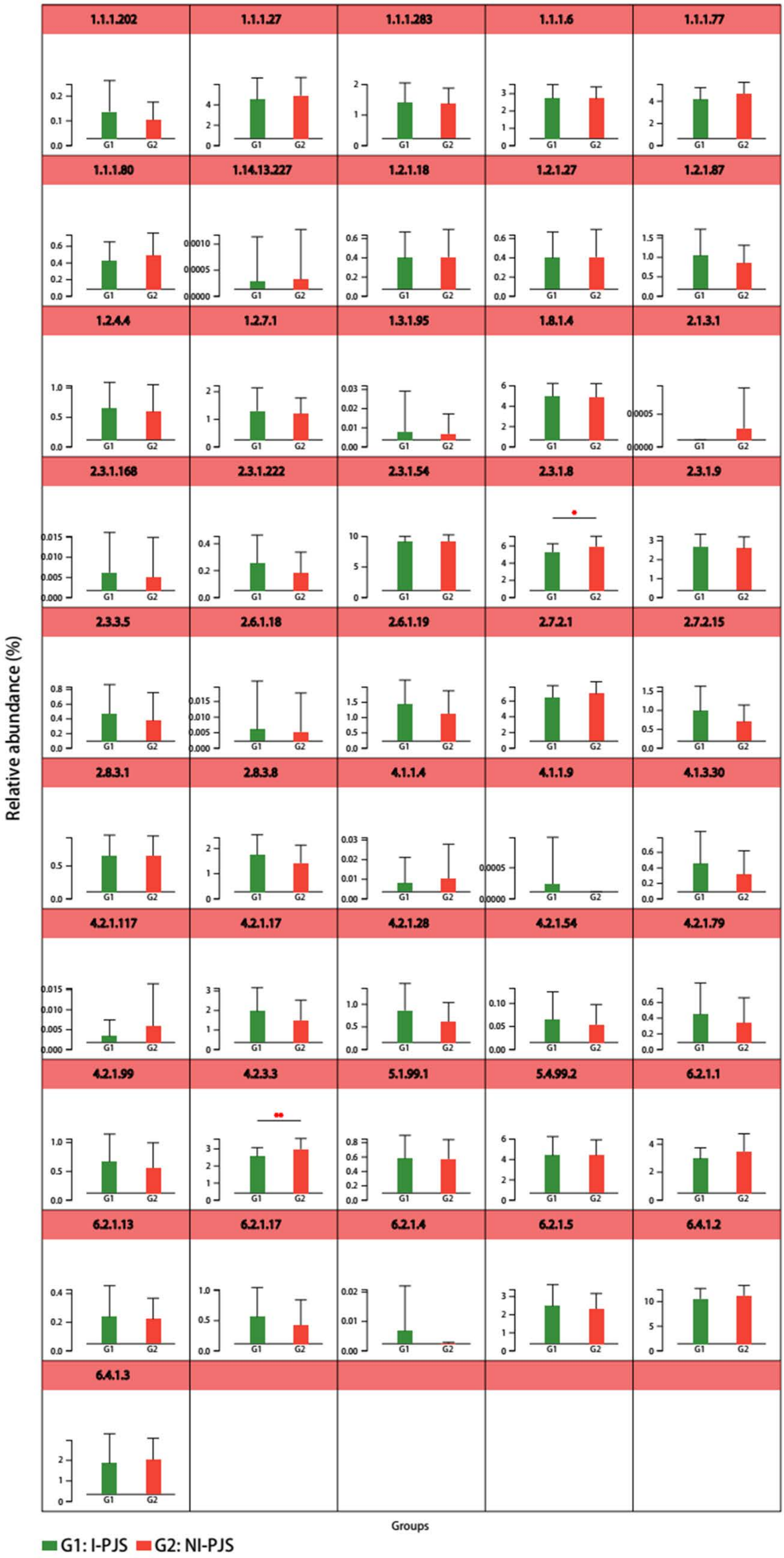

1.1.1.202
1.1.1.27
1.1.1.283
1.1.1.6
1.1.1.77
1.1.1.80
1.14.13.227
1.2.1.18
1.2.1.27
1.2.1.87
1.2.4.4
1.2.7.1
1.3.1.95
1.8.1.4
2.1.3.1
2.3.1.168
2.3.1.222
2.3.1.54
2.3.1.8
2.3.1.9
2.3.3.5
2.6.1.18
2.6.1.19
2.7.2.1
2.7.2.15
2.8.3.1
2.8.3.8
4.1.1.4
4.1.1.9
4.1.3.30
4.2.1.117
4.2.1.17
4.2.1.28
4.2.1.54
4.2.1.79
4.2.1.99
4.2.3.3
5.1.99.1
5.4.99.2
6.2.1.1
6.2.1.13
6.2.1.17
6.2.1.4
6.2.1.5
6.4.1.2
6.4.1.3
Relative abundance (%)
Groups
G1: I-PJS
G2: NI-PJS

Figure S7

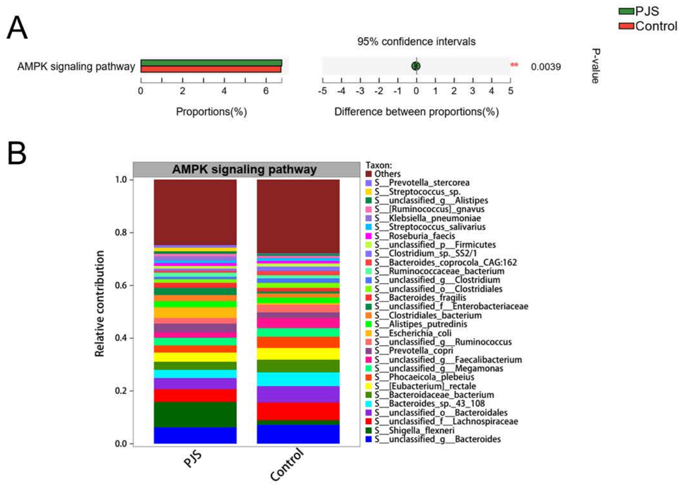
A
PJS
Control
95% confidence intervals
AMPK signaling pathway
0.0039
P-value
0
2
4
6
Proportions(%)
-5 -4 -3 -2 -1 0 1 2 3 4 5
Difference between proportions(%)
B
AMPK signaling pathway
Relative contribution
1.0
0.8
0.6
0.4
0.2
0.0
PJS
Control
Taxon:
Others
S__Prevotella_stercorea
S__Streptococcus_sp.
S__unclassified_g__Alistipes
S__[Ruminococcus]_gnavus
S__Klebsiella_pneumoniae
S__Streptococcus_salivarius
S__Roseburia_faecis
S__unclassified_p__Firmicutes
S__Clostridium_sp._SS2/1
S__Bacteroides_coprocola_CAG:162
S__Ruminococcaceae_bacterium
S__unclassified_g__Clostridium
S__unclassified_o__Clostridiales
S__Bacteroides_fragilis
S__unclassified_f__Enterobacteriaceae
S__Clostridiales_bacterium
S__Alistipes_putredinis
S__Escherichia_coli
S__unclassified_g__Ruminococcus
S__Prevotella_copri
S__unclassified_g__Faecalibacterium
S__unclassified_g__Megamonas
S__Phocaeicola_plebeius
S__[Eubacterium]_rectale
S__Bacteroidaceae_bacterium
S__Bacteroides_sp._43_108
S__unclassified_o__Bacteroidales
S__unclassified_f__Lachnospiraceae
S__Shigella_flexneri
S__unclassified_g__Bacteroides

Table S1. Different genera identified between PJS patients and healthy family controls

Genus	PJS-mean	control-mean	P value
g__Faecalibacterium	13.2624	16.5290	0.0409
g__Escherichia-Shigella	10.3316	3.2906	0.0000
g__Klebsiella	3.6317	1.4302	0.0000
g__Megamonas	2.0016	3.0393	0.0119
g__Subdoligranulum	1.5193	2.2965	0.0221
g__Fusobacterium	1.4126	0.4845	0.0002
g__Sutterella	0.5758	0.8790	0.0169
g__Lactobacillus	0.1606	1.4040	0.0034
g__Anaerostipes	0.4603	0.5763	0.0123
g__Veillonella	0.5038	0.4356	0.0052
g__Romboutsia	0.4386	0.3754	0.0007
g__Intestinibacter	0.3571	0.4527	0.0010
g__Akkermansia	0.3219	0.1958	0.0007
g__Enterobacter	0.2078	0.2790	0.0138
g__Erysipelotrichaceae_UCG-003	0.1887	0.2756	0.0051
g__Mitsuokella	0.0429	0.4337	0.0393
g__Muribaculaceae_unclassified	0.1710	0.0905	0.0001
g__Ruminococcus	0.1308	0.1855	0.0296
g__Haemophilus	0.1128	0.1048	0.0252
g__Actinobacteria_unclassified	0.1131	0.0018	0.0000
g__Lachnospiraceae_UCG-003	0.0523	0.0861	0.0226
g__Terrisporobacter	0.0492	0.0770	0.0045
g__Enterococcus	0.0520	0.0137	0.0259
g__Pseudomonas	0.0364	0.0021	0.0000
g__Plesiomonas	0.0365	0.0020	0.0309
g__Aeromonas	0.0284	0.0119	0.0101
g__Sphingopyxis	0.0224	0.0076	0.0005
g__Family_XIII_UCG-001	0.0120	0.0188	0.0103
g__Delftia	0.0055	0.0328	0.0000
g__Asteroleplasma	0.0000	0.0392	0.0259
g__Caulobacter	0.0147	0.0002	0.0000
g__Ruminococcaceae_UCG-009	0.0077	0.0103	0.0080
g__Oxyphotobacteria_unclassified	0.0088	0.0067	0.0002
g__Adlercreutzia	0.0053	0.0129	0.0385
g__Proteus	0.0096	0.0002	0.0281
g__Coprobacillus	0.0079	0.0034	0.0498
g__Lachnospiraceae_NK4A136_group	0.0067	0.0006	0.0000
g__Burkholderia-Caballeronia-Paraburkholderia	0.0000	0.0159	0.0000
g__Clostridiales_Family_XIV._Incertae_Sedis_unclas	0.0058	0.0017	0.0123
g__Phocea	0.0044	0.0045	0.0445
g__Peptostreptococcaceae_unclassified	0.0035	0.0041	0.0227
g__Gammaproteobacteria_unclassified	0.0031	0.0047	0.0001
g__Sphingomonas	0.0043	0.0011	0.0004
g__Rhizobacter	0.0035	0.0000	0.0000
g__Methylibium	0.0026	0.0000	0.0001
g__TRA3-20_unclassified	0.0024	0.0004	0.0237
g__Ruminococcaceae_UCG-008	0.0003	0.0052	0.0032
g__Providencia	0.0007	0.0032	0.0000
g__Coriobacteriaceae_UCG-002	0.0017	0.0001	0.0042
g__Anaerofilum	0.0008	0.0025	0.0069
g__Bradyrhizobium	0.0017	0.0003	0.0094
g__Abiotrophia	0.0017	0.0002	0.0239
g__Photobacterium	0.0000	0.0029	0.0004
g__Sphaerotilus	0.0000	0.0024	0.0000

g__Enorma	0.0000	0.0023	0.0259
g__Corynebacterium_1	0.0004	0.0013	0.0466
g__Clostridium_sensu_stricto_5	0.0000	0.0020	0.0004
g__Rhizobiales_Incertae_Sedis_unclassified	0.0009	0.0000	0.0039
g__cvE6_unclassified	0.0007	0.0002	0.0330
g__Oblitimonas	0.0000	0.0017	0.0000
g__hgcI_clade	0.0000	0.0013	0.0004
g__Sporichthyaceae_unclassified	0.0001	0.0012	0.0031
g__Selenomonas	0.0001	0.0012	0.0357
g__Lysinibacillus	0.0000	0.0010	0.0004
g__Pluralibacter	0.0000	0.0011	0.0063
g__Subgroup_2_unclassified	0.0000	0.0010	0.0063
g__Sphingosinicella	0.0001	0.0007	0.0189
g__Proteobacteria_unclassified	0.0000	0.0010	0.0397
g__Elizabethkingia	0.0000	0.0006	0.0004
g__Chitinimonas	0.0000	0.0008	0.0016
g__Limnohabitans	0.0000	0.0006	0.0063
g__Papillibacter	0.0000	0.0006	0.0106
g__Lachnospiraceae_NK4B4_group	0.0000	0.0007	0.0259
g__Myroides	0.0000	0.0006	0.0259
g__Serratia	0.0001	0.0005	0.0406
g__Elsterales_unclassified	0.0000	0.0004	0.0016
g__Tissierella	0.0000	0.0002	0.0063
g__Proteocatella	0.0000	0.0002	0.0063
g__Eggerthellaceae_unclassified	0.0000	0.0004	0.0259
g__Rhodoferax	0.0000	0.0003	0.0259
g__Anaerovibrio	0.0000	0.0003	0.0259
g__Candidatus_Solibacter	0.0000	0.0003	0.0259
g__Lysobacter	0.0000	0.0003	0.0259
g__Rhodanobacter	0.0000	0.0003	0.0259
g__Proteiniphilum	0.0000	0.0002	0.0259
g__Acetobacter	0.0000	0.0002	0.0259
g__KF-JG30-C25_unclassified	0.0000	0.0002	0.0259
g__Synergistes	0.0001	0.0003	0.0406
g__Savagea	0.0000	0.0002	0.0259
g__Brachybacterium	0.0000	0.0001	0.0259
g__Phreatobacter	0.0000	0.0001	0.0259
g__W5053	0.0000	0.0001	0.0259

Table S2. Different species identified between PJS patients and healthy family controls

species	Control-Mean	PJS-Mean	P value
s Faecalibacterium prausnitzii	6.661	4.785	0.02855
s unclassified o Clostridiales	4.078	3.191	0.009411
s unclassified d Bacteria	1.439	1.781	0.01691
s Bifidobacterium longum	1.279	0.9056	0.03912
s Escherichia coli	1.114	5.949	0.0008418
s Anaerostipes hadrus	0.7635	0.372	0.0122
s unclassified f Enterobacteriaceae	0.5491	2.547	2.39E-05
s unclassified g Blautia	0.5151	0.3822	0.0122
s Klebsiella pneumoniae	0.4773	1.725	0.0001446
s Faecalibacterium sp.	0.4378	0.3053	0.01532
s Anaerobutyricum hallii	0.3797	0.2997	0.008693
s Dorea longicatena	0.3673	0.2629	0.009662
s Romboutsia timonensis	0.271	0.1348	0.004248
s unclassified p Proteobacteria	0.2244	1.176	0.0007631
s Azospirillum sp. 51 20	0.2211	0.04013	0.03236
s Dorea formicigenerans	0.2175	0.1594	0.0279
s Streptococcus salivarius	0.2103	0.1943	0.02207
s uncultured Faecalibacterium sp.	0.1699	0.1153	0.03999
s unclassified f Erysipelotrichaceae	0.1678	0.08651	0.006824
s Intestinibacter bartlettii	0.151	0.05049	5.67E-05
s [Ruminococcus] lactaris	0.1367	0.09478	0.04458
s unclassified g Klebsiella	0.1332	0.5105	0.0002568
s unclassified g Dorea	0.1018	0.07105	0.03999
s Clostridium sp. AF27-2AA	0.07007	0.03215	0.003473
s Clostridium sp. SS2/1	0.05779	0.03341	0.02155
s Faecalibacterium sp. OM04-11BH	0.05742	0.0358	0.01911
s Clostridium sp. AM22-11AC	0.05594	0.02996	0.04177
s Clostridium sp. AM33-3	0.05267	0.02044	0.002096
s Blautia sp. KLE 1732	0.04712	0.03212	0.02855
s Clostridium disporicum	0.04543	0.01284	0.004371
s Actinomyces sp. ICM47	0.04419	0.02213	0.0496
s Turicibacter sanguinis	0.04284	0.02512	0.04653
s Streptococcus parasanguinis	0.04194	0.02505	0.007402
s Firmicutes bacterium CAG:227	0.03859	0.01557	0.0113
s Shigella sonnei	0.03736	0.2017	0.0008148
s Lachnospiraceae bacterium 5 1 63FAA	0.03711	0.01955	0.0157
s Salmonella enterica	0.03627	0.1588	0.0008983
s uncultured Eubacterium sp.	0.03594	0.02376	0.003
s Rothia mucilaginosa	0.03478	0.01251	0.0005295
s unclassified g Escherichia	0.03396	0.1957	0.00136
s Klebsiella variicola	0.03087	0.1396	0.0001077
s Anaerostipes caccae	0.03035	0.03805	0.03581
s Eubacterium sp. CAG:248	0.03009	0.01004	0.00525
s unclassified g Delftia	0.03	0.0004856	0.009104
s Lachnospiraceae bacterium AM26-1LB	0.02953	0.01564	0.007811
s Actinomyces graevenitzii	0.02951	0.00795	0.04856
s Firmicutes bacterium AF12-30	0.029	0.006687	0.01421
s unclassified o Enterobacterales	0.0286	0.1162	4.32E-05
s Clostridium sp. CAG:221	0.02805	0.007503	0.01071
s Blautia hydrogenotrophica	0.02705	0.003983	0.02543
s Shigella flexneri	0.02653	0.1445	0.0008983
s Frisingicoccus caecimuris	0.02617	0.00421	0.03502
s Streptococcus sanguinis	0.02338	0.006261	0.007812
s Acinetobacter baumannii	0.02321	0.07213	0.008927
s Romboutsia ilealis	0.02293	0.01155	0.002665
s Firmicutes bacterium AM10-47	0.02185	0.009399	0.006461
s Klebsiella quasipneumoniae	0.02155	0.08205	0.0001446

s	Actinomyces sp. ICM58	0.02127	0.006235	0.01421
s	Clostridium sp. TF06-15AC	0.02126	0.01641	0.003575
s	[Bacteroides] pectinophilus	0.02105	0.0166	0.04269
s	Clostridiales bacterium 36 14	0.0204	0.01453	0.04269
s	Streptococcus mitis	0.01924	0.02062	0.002913
s	Schaalia odontolytica	0.01876	0.007956	0.001448
s	Actinomyces sp. oral taxon 172	0.01805	0.005237	0.01457
s	Butyricicoccus sp. AF24-19AC	0.01799	0.01363	0.006461
s	Firmicutes bacterium CAG:270	0.01798	0.003281	0.01609
s	unclassified g Turicibacter	0.01781	0.01041	0.001063
s	Enterobacter hormaechei	0.0178	0.03019	0.008465
s	unclassified g Rothia f Micrococcaceae	0.01752	0.006755	0.0001556
s	Streptococcus anginosus	0.01736	0.009004	0.04087
s	Enterobacter cloacae	0.01708	0.02164	0.005474
s	Clostridiales bacterium Nov 37 41	0.01642	0.01015	0.02664
s	Clostridium saudiense	0.01582	0.003938	0.0032
s	Clostridium sp. AM42-4	0.01536	0.008975	0.01532
s	unclassified p Actinobacteria	0.01524	0.007042	0.001972
s	Faecalibacterium sp. BIOML-A1	0.01506	0.007914	0.03825
s	Butyricicoccus sp. OF10-2	0.01496	0.01104	0.01421
s	Clostridium sp. 29 15	0.01493	0.002017	0.01953
s	Clostridium bartlettii CAG:1329	0.0147	0.004943	0.0009278
s	Peptostreptococcaceae bacterium	0.01454	0.008341	0.00116
s	Lachnospiraceae bacterium AM21-21	0.01425	0.007321	0.01475
s	Clostridium sp. OM04-12AA	0.01396	0.009312	0.02922
s	Streptococcus pneumoniae	0.0139	0.01448	0.03581
s	Enterobacter kobei	0.01356	0.01377	0.02922
s	Butyricicoccus sp. OM06-6AC	0.01335	0.009181	0.008242
s	Shigella dysenteriae	0.01284	0.06949	0.0006689
s	Streptococcus vestibularis	0.0125	0.01213	0.01494
s	Lachnospiraceae bacterium CAG:25	0.01243	0.006564	0.01909
s	Streptococcus sp. 263 SSPC	0.01219	0.007232	0.03661
s	Terrisporobacter othiniensis	0.01207	0.006688	0.0002955
s	unclassified f Sutterellaceae	0.01207	0.007061	0.03166
s	Eisenbergiella massiliensis	0.01205	0.009225	0.03827
s	Lachnospiraceae bacterium AM10-38	0.0119	0.005543	0.007012
s	Clostridium sp. AF29-8BH	0.01181	0.007716	0.03581
s	Aureimonas altamirensis	0.01177	0.00826	0.01332
s	Amedibacillus dolichus	0.01149	0.00612	0.03058
s	Terrisporobacter glycolicus	0.01142	0.006708	0.0008696
s	Clostridium sp. 1 1 41A1FAA	0.01025	0.003592	0.0001671
s	Ruminococcus sp. AF14-10	0.01022	0.007142	0.04087
s	Streptococcus rubneri	0.01009	0.007349	0.003788
s	Clostridium sp. OF09-36	0.01009	0.005969	0.004758
s	Clostridium sp. AF27-5AA	0.009996	0.006605	0.0213
s	Paeniclostridium sordellii	0.009934	0.006253	0.02922
s	Firmicutes bacterium AM43-11BH	0.009608	0.005995	0.01776
s	uncultured bacterium 405006-B04	0.009468	0.007793	0.04177
s	Clostridium sp. AF32-12BH	0.009422	0.00812	0.04087
s	Anaeromassilibacillus sp. An250	0.009194	0.007808	0.04087
s	Prevotella stercorea	0.009152	0.2025	0.03955
s	Shigella boydii	0.009081	0.04808	0.001318
s	Ruminococcus sp. OF03-6AA	0.009034	0.00431	0.009166
s	Streptococcus mutans	0.008645	0.002444	0.04362
s	Blautia sp. AM22-22LB	0.008465	0.005019	0.02603
s	Clostridium sp. AM48-13	0.008464	0.006745	0.02922
s	Clostridium sp. CAG:265	0.008331	0.001185	0.004741
s	Enterocloster citroniae	0.008273	0.005851	0.032
s	Clostridium sp. AF22-10	0.008019	0.004299	0.02543

s	Butyricicoccus sp. AF15-40	0.007823	0.005445	0.03423
s	Streptococcus oralis	0.007775	0.01055	0.006461
s	Megamonas sp. Calf98-2	0.007594	0.009505	0.04793
s	Enterobacter roggenkampii	0.007548	0.009043	0.03057
s	Eubacterium hallii CAG:12	0.007545	0.005728	0.01607
s	Clostridium sp. OF13-4	0.007449	0.005389	0.008465
s	Sanguibacter keddieii	0.007436	0.002797	0.0006259
s	Macrococcus caseolyticus	0.007374	0.0005662	0.01558
s	Dorea sp. BIOML-A1	0.007296	0.005368	0.03386
s	Clostridium celatum	0.006928	0.01032	0.004497
s	Clostridium cuniculi	0.006725	0.002183	0.001905
s	Propionibacterium acidifaciens	0.006702	0.001518	0.02726
s	Lachnospiraceae bacterium TF10-8AT	0.00669	0.00423	0.02315
s	Clostridium sp. OM02-18AC	0.006659	0.004857	0.02104
s	unclassified c Gammaproteobacteria	0.006538	0.03422	5.45E-05
s	Ruminococcus sp. OM08-9BH	0.006419	0.003396	0.01776
s	Enterococcus durans	0.006206	0.004118	0.01317
s	Fusobacterium sp. CM21	0.006153	0.004273	0.01144
s	Anaeromassilibacillus senegalensis	0.006071	0.002888	0.007707
s	Actinomyces sp. HPA0247	0.00607	0.002408	0.01733
s	Faecalibacterium sp. BIOML-A3	0.006003	0.003669	0.03273
s	Klebsiella aerogenes	0.005955	0.02302	0.0001077
s	Dorea sp. OM02-2LB	0.005927	0.003525	0.03581
s	Actinomyces sp. HMSC035G02	0.005874	0.002266	0.001055
s	unclassified o Lactobacillales	0.005833	0.009578	0.04362
s	uncultured Dorea sp.	0.005809	0.004397	0.03348
s	Isoptericola variabilis	0.005668	0.002257	0.01351
s	Mycobacteroides abscessus	0.00552	0.002162	0.0004471
s	Ruminococcus gauvreauii	0.005483	0.001681	0.02955
s	Mediterranea massiliensis	0.005347	0.001819	0.04753
s	Citrobacter freundii	0.005307	0.009591	0.005629
s	Ruminococcus sp. AM22-14LB	0.005231	0.003282	0.01457
s	Ruminococcus sp. CAG:90	0.005204	0.003396	0.02989
s	Dorea sp. Marseille-P4042	0.005173	0.003725	0.04177
s	Faecalibacillus faecis	0.005162	0.002961	0.03912
s	Absiella sp. AM27-20	0.005151	0.001735	0.004894
s	Prevotella sp. CAG:732	0.005081	0.0133	0.04243
s	Coprobacillus sp. TM10-10	0.005025	0.001874	0.02829
s	Absiella argi	0.004939	0.001908	0.002913
s	unclassified g Citrobacter	0.004869	0.004475	0.03743
s	Klebsiella sp. MS 92-3	0.004854	0.01889	9.63E-05
s	Ruminococcus sp. AM26-12LB	0.004769	0.003319	0.032
s	Enterobacter ludwigii	0.004513	0.009972	0.001161
s	Enterobacter asburiae	0.004491	0.01374	0.0003517
s	Escherichia fergusonii	0.004468	0.0235	0.0009897
s	Escherichia albertii	0.004451	0.02505	0.001404
s	Clostridium sp. KLE 1755	0.004404	0.002853	0.03581
s	Erysipelotrichaceae bacterium AM17-60	0.004175	0.001537	0.002064
s	Faecalibacterium sp. BIOML-A2	0.004161	0.002377	0.02362
s	Streptococcus sp. ACS2	0.004141	0.003778	0.0226
s	Clostridiales bacterium CHKCI006	0.004112	0.003145	0.03129
s	Lachnospiraceae bacterium Choco86	0.0041	0.002653	0.04223
s	Streptococcus australis	0.004033	0.002766	0.0007886
s	Romboutsia sp. CE17	0.003896	0.001827	0.01828
s	Streptococcus sp. SR4	0.003853	0.003569	0.01457
s	Dorea sp. 42 8	0.003738	0.00266	0.01797
s	Klebsiella oxytoca	0.003653	0.01095	0.004128
s	Desulfovibrio sp.	0.003549	0.01079	0.04199
s	Cronobacter sakazakii	0.003496	0.02362	0.0002568

s	Eubacterium sp. Marseille-P5640	0.003431	0.001369	0.01144
s	Streptococcus gordonii	0.003377	0.00177	0.002365
s	Streptococcus cristatus	0.003298	0.003201	0.0001038
s	[Eubacterium] sulci	0.003248	0.001874	0.02315
s	Hungateiclostridium thermocellum	0.003184	0.002733	0.0237
s	Firmicutes bacterium CAG:212	0.003146	0.00175	0.001494
s	Clostridiales bacterium VE202-06	0.003096	0.003277	0.03827
s	Kluyvera ascorbata	0.003075	0.001688	0.001682
s	Streptococcus pseudopneumoniae	0.003075	0.003179	0.00368
s	unclassified g Actinomyces	0.003061	0.001588	0.0165
s	Flavonifractor sp. An92	0.003043	0.002891	0.03274
s	Chryseobacterium cucumeris	0.002979	0.002074	0.01047
s	Actinomyces sp. ICM54	0.002918	0.001161	0.003575
s	Bifidobacterium sp. WCA-178-WT-4B	0.002916	0.001995	0.03842
s	Pseudomonas stutzeri	0.002828	0.01076	0.004685
s	Streptococcus sp. I-P16	0.002827	0.001384	0.001692
s	Klebsiella michiganensis	0.00279	0.01323	0.0001932
s	Romboutsia sp. Marseille-P6047	0.002753	0.001526	0.0009868
s	Bifidobacterium myosotis	0.002644	0.001888	0.01405
s	Dorea formicigenerans CAG:28	0.002639	0.001767	0.005176
s	Romboutsia sp.	0.002628	0.001263	0.007304
s	Carboxydocella sp. ULO1	0.002615	0.001519	0.01363
s	Ruminococcus sp. 1xD21-23	0.002543	0.001544	0.04315
s	Serratia quinivorans	0.00254	0.0115	4.15E-05
s	unclassified g Granulicatella	0.002507	0.001441	0.01911
s	Coprobacillus sp. 8 1 38FAA	0.002458	0.001553	0.0165
s	Coprobacillus sp. AF16-47	0.002434	0.001148	0.03643
s	Coprobacillus sp. AM09-26	0.002427	0.001406	0.03246
s	Turicibacter sp. H121	0.002411	0.001938	0.0007751
s	bacterium D16-51	0.002397	0.001804	0.0226
s	Mediterraneibacter massiliensis	0.002392	0.002047	0.03743
s	Clostridiales bacterium VE202-27	0.002322	0.001492	0.03999
s	Coprobacillus sp. AM37-9BH	0.002318	0.0009273	0.03686
s	Actinomyces sp. ICM39	0.002293	0.0009358	0.0001932
s	Streptococcus infantis	0.002259	0.001556	0.001448
s	Proteobacteria bacterium	0.002214	0.005586	6.86E-05
s	Paraclostridium bifermentans	0.002214	0.001426	0.04718
s	Streptococcus sp. F0442	0.00221	0.001284	0.002549
s	Turicibacter sp. HGF1	0.00207	0.0003444	0.01324
s	Romboutsia lituseburensis	0.002038	0.001324	0.009955
s	Shigella sp.	0.002007	0.01146	0.001055
s	Rothia aeria	0.001997	0.001078	0.03999
s	Xylanimonas cellulosilytica	0.001983	0.0008584	0.01385
s	Streptococcus sp. 449 SSPC	0.001956	0.001571	0.01284
s	Bacteroides sp. AF32-8BH	0.001927	0.003153	0.04753
s	Bacteroides sp. CAG:702	0.001899	0.001156	0.04312
s	Clostridium sp. AF19-22AC	0.001888	0.001504	0.002295
s	unclassified f Actinomycetaceae	0.001884	0.0005603	0.002227
s	Streptococcus sp. HMSC064H09	0.001876	0.00136	0.008925
s	Coprococcus sp. CAG:131	0.001817	0.004922	0.02193
s	Streptococcus salivarius CAG:79	0.001806	0.001547	0.0213
s	Streptococcus sp. I-G2	0.001798	0.001051	0.001199
s	Coprobacillus sp. AF35-8	0.001793	0.0009606	0.04804
s	Streptococcus sp. HMSC064D12	0.001739	0.001745	0.01958
s	Olsenella sp. AM39-30AC	0.001684	0.0002386	0.00796
s	Clostridium ventriculi	0.001679	0.000118	0.006964
s	Clostridiales bacterium VE202-28	0.001652	0.001058	0.04362
s	Granulicatella adiacens	0.001646	0.0009967	0.02664
s	Streptococcus sp. FDAARGOS 192	0.001633	0.001307	0.0122

s	Enterobacter sp. BIDMC92	0.001614	0.001202	0.001021
s	Lachnospiraceae bacterium AM23-7LB	0.001607	0.0007659	0.0344
s	Streptococcus sp. CCH8-H5	0.001591	0.001718	0.02664
s	Bacillus subtilis	0.001555	0.001224	0.001237
s	Pseudoflavonifractor sp. BSD2780061688st1	0.001542	0.001078	0.0122
s	Streptococcus sp. AM28-20	0.001512	0.0008165	0.007599
s	Intestinibaculum porci	0.001508	0.001176	0.0362
s	Clostridium sporogenes	0.001485	0.0007439	0.003522
s	Raoultella ornithinolytica	0.00148	0.004742	0.001687
s	Streptococcus koreensis	0.001456	0.0007015	0.001639
s	Streptococcus sp. HMSC068F04	0.001441	0.001328	0.01351
s	Streptococcus sp. HSISS2	0.001433	0.001313	0.02342
s	unclassified g Shigella	0.001393	0.007993	0.001448
s	Citrobacter koseri	0.001375	0.007089	0.0001864
s	Enterobacter bugandensis	0.001359	0.00109	0.02362
s	Peptostreptococcaceae bacterium VA2	0.00135	0.0009999	0.04632
s	Streptococcus sp. AM43-2AT	0.001342	0.0007429	0.00941
s	unclassified g Cronobacter	0.00133	0.01014	0.0002853
s	Clostridia bacterium UC5.1-1D1	0.00133	0.001033	0.04456
s	Photorhabdus luminescens	0.001327	0.005471	0.0001249
s	Rothia sp. HMSC061D12	0.00129	0.0005007	0.0009269
s	Streptococcus sp. C150	0.001283	0.001076	0.01776
s	Clostridium puniceum	0.00126	0.0004299	0.004609
s	Kluyvera georgiana	0.001254	0.002093	0.01729
s	Streptococcus viridans	0.001246	0.0007325	0.001125
s	Streptococcus sp. HSISM1	0.00124	0.0007205	0.009408
s	Rothia sp. HMSC061E04	0.001228	0.0004126	0.001105
s	Morganella morganii	0.001205	0.01185	6.86E-05
s	Prevotella stercorea CAG:629	0.001197	0.03803	0.03415
s	Novimethylophilus kurashikiensis	0.001177	0.0007669	0.003
s	Enterobacter sp. FY-07	0.00117	0.004823	0.0002752
s	Romboutsia weinsteinii	0.00117	0.0006035	0.006158
s	Rothia sp. HMSC071B01	0.001164	0.0003521	0.001313
s	Streptococcus sp. HSISS1	0.001159	0.0009672	0.004893
s	Shigella sp. SF-2015	0.001134	0.006551	0.001124
s	Corynebacterium striatum	0.001129	0.0005814	0.01384
s	Clostridium sp. C105KSO14	0.001099	0.0004926	0.01981
s	Streptococcaceae bacterium	0.001091	0.0007262	0.001277
s	Prevotella ruminicola	0.001083	0.003543	0.003455
s	TM7 phylum sp. oral taxon 352	0.001069	0.000852	0.005103
s	Streptococcus sp. oral taxon 058	0.00106	0.001141	0.01058
s	Rothia sp. HMSC069C10	0.001043	0.0006105	0.001213
s	Clostridium sp. AF12-19	0.001037	0.0005947	0.004555
s	Serratia marcescens	0.001033	0.003615	0.0005475
s	Dialister sp. CAG:486	0.001019	0.0009936	0.02499
s	Rothia sp. HMSC065C03	0.001013	0.0003931	0.000328
s	Olsenella sp. AM30-3LB	0.001006	0.0001413	0.0275
s	Streptococcus periodonticum	0.001001	0.0003249	0.01569
s	Streptococcus intermedius	0.0009905	0.0003774	0.007003
s	Coprococcus eutactus CAG:665	0.000944	0.005584	0.002028
s	Schaalia turicensis	0.0009414	0.0003108	0.004228
s	Serratia liquefaciens	0.0009376	0.003878	0.001099
s	Streptococcus sp. HMSC061E03	0.0009364	0.0006491	0.003087
s	Rothia sp. HMSC065B04	0.000929	0.0003312	0.001967
s	Enterobacter cancerogenus	0.0009268	0.004087	0.0003397
s	Streptococcus sp. HMSC065E03	0.0009087	0.0004851	0.004011
s	Streptococcus sp. HMSC072D03	0.0008863	0.0006745	0.04554
s	Rothia sp. HMSC071C12	0.0008828	0.0003362	0.0003274
s	Streptococcus sp. LPB0220	0.0008745	0.0004141	0.00107

s	Clostridium roseum	0.0008711	0.0001558	0.009999
s	Rothia sp. HMSC069C03	0.0008697	0.0003577	0.0006451
s	Rothia sp. HMSC076D04	0.0008656	0.0003168	0.0007617
s	Ruminococcaceae bacterium HV4-5-B5C	0.0008492	0.0005134	0.0191
s	Actinomyces sp. oral taxon 897	0.0008406	0.0003455	0.02656
s	Rothia sp. HMSC068F09	0.0008385	0.0002394	0.00356
s	Enterobacter sp. MGH 25	0.0008382	0.0009324	0.001745
s	Rothia sp. HMSC061C12	0.0008301	0.000302	0.0003491
s	Streptococcus sp. HMSC072G04	0.0008285	0.0004391	0.003787
s	Streptococcus sp. HMSC074F05	0.0008278	0.000499	0.007007
s	Raoultella terrigena	0.0008258	0.003691	1.46E-05
s	Streptococcus sp. HMSC072C09	0.0008244	0.0004409	0.0007753
s	Rothia sp. HMSC072B04	0.0008124	0.0003501	0.001016
s	Trichococcus palustris	0.0008121	0.000796	0.01698
s	Rothia sp. HMSC068E02	0.000808	0.0002788	0.003301
s	Gordonibacter sp. An230	0.0008024	0.0003366	0.02977
s	Streptococcus sp. A12	0.0008005	0.0005039	0.001125
s	Rothia sp. HMSC066G07	0.0008002	0.0003463	0.001234
s	Actinomyces sp.	0.0007921	0.0003657	0.03785
s	[Clostridium] dakarense	0.000789	0.0004169	0.009881
s	Yokenella regensburgei	0.0007845	0.004222	5.89E-05
s	Emergencia sp. 1XD21-10	0.0007824	0.001503	0.04129
s	Streptococcus sp. HMSC073D05	0.0007751	0.0004577	0.009655
s	Streptococcus constellatus	0.0007684	0.0005868	0.0009566
s	Sulfuriferula sp. AH1	0.0007607	0.0031	0.0001394
s	Rothia sp. HMSC064F07	0.0007542	0.0003407	0.003166
s	Mobiluncus mulieris	0.0007423	8.63E-05	0.04123
s	Streptococcus sp. HMSC078D09	0.0007392	0.0003746	0.008236
s	Rothia sp. HMSC069C04	0.000733	0.0002736	0.0001659
s	Firmicutes bacterium M10-2	0.0007321	0.0002083	0.02453
s	Rothia sp. HMSC073B08	0.0007197	0.0003219	0.003864
s	Staphylococcus epidermidis	0.000706	0.003209	0.005032
s	Streptococcus sp. CCH5-D3	0.0006907	0.000561	0.02101
s	Coprobacillus sp. AF34-1BH	0.0006902	0.0003512	0.02776
s	Rothia sp. HMSC066G02	0.0006803	0.0002388	0.0001444
s	Achromobacter sp. ATCC35328	0.0006747	0.003333	0.002226
s	Klebsiella quasivariicola	0.0006733	0.002471	0.0005408
s	Coprobacillus sp. AF21-8LB	0.0006714	0.0003889	0.04176
s	Rothia sp. HMSC075F09	0.0006675	0.0002481	0.0005927
s	Clostridium sp. CAG:356	0.0006627	0.002146	0.04254
s	Streptococcus timonensis	0.0006622	0.001334	0.001852
s	Kluyvera cryocrescens	0.0006613	0.002193	0.00123
s	unclassified p Candidatus Saccharibacteria	0.0006561	0.0005494	0.009393
s	Rothia sp. HMSC078H08	0.0006522	0.0002568	0.0003098
s	Negativicoccus succinicivorans	0.0006458	0.0003977	0.004148
s	Enterococcus thailandicus	0.0006347	0.0001992	0.003197
s	Arabia massiliensis	0.0006297	0.0002931	0.03729
s	Raoultella planticola	0.0006223	0.001721	0.001274
s	Rothia sp. HMSC062H08	0.0006128	0.0002305	0.0003614
s	Streptococcus sp. 343 SSPC	0.0006103	0.0004392	0.003082
s	Dubosiella newyorkensis	0.0006011	0.0004058	0.02888
s	Rothia sp. HMSC062F03	0.0005926	0.0001656	0.002331
s	Streptococcus sp. M334	0.0005872	0.0006599	0.03954
s	Rothia sp. HMSC065C12	0.000581	0.0002303	0.0015
s	Citrobacter youngae	0.0005809	0.001208	0.02154
s	Rothia sp. HMSC072E10	0.0005805	0.0001234	0.02652
s	Turicibacter sp. TS3	0.0005798	0.0005754	0.006589
s	Gemella sanguinis	0.0005724	0.0003711	0.008668
s	Kytococcus sedentarius	0.0005723	0.0002664	0.03743

s	Streptococcus sp. HSISS3	0.0005695	0.0005774	0.007698
s	Propionispora vibrioides	0.0005648	4.48E-05	0.02202
s	Streptococcus sp. HMSC057G03	0.0005601	0.0002992	0.004493
s	Ignavibacteriae bacterium HGW-Ignavibacte	0.0005597	0	0.03398
s	Rothia sp. HMSC072B03	0.000553	0.0001546	0.006633
s	Escherichia sp. KTE172	0.0005385	0.001654	0.04822
s	unclassified g Aeromonas	0.0005378	0.01631	0.0003899
s	Actinomyces sp. oral taxon 180	0.0005328	0.0001976	0.04457
s	Streptococcus sp. HMSC065H07	0.0005246	0.0004217	0.00503
s	Romboutsia hominis	0.0005236	0.0003259	0.002038
s	Candidatus Stoquefichus sp. SB1	0.0005199	0.0001848	0.02584
s	Enterobacter sp. KINAN-G	0.0005085	0.0008968	0.001021
s	Streptococcus sp. HMSC076C09	0.0005028	0.0002951	0.003572
s	Paraclostridium benzoelyticum	0.0005017	0.0003394	0.03773
s	Escherichia sp. R8	0.0004939	0.002646	0.003864
s	Escherichia sp. 4 1 40B	0.0004867	0.002551	0.002906
s	Rothia sp. HMSC069D01	0.0004866	0.0001557	0.0001628
s	Eggerthella timonensis	0.000482	0.0003884	0.03492
s	Acetobacterium bakii	0.000477	0.0001453	0.001379
s	Clostridium chromiireducens	0.0004738	0.0004019	0.04511
s	Streptococcus sp. HMSC065C01	0.0004675	0.0002526	0.001316
s	Massilimicrobiota sp. SW1139	0.0004453	0.0001286	0.01892
s	Streptococcus sp. 1171 SSPC	0.0004385	0.000281	0.003662
s	Mogibacterium diversum	0.0004289	0.0001744	0.01142
s	Streptococcus sp. UMB0029	0.0004132	0.0007189	0.0074
s	Desulfovibrio sp. PG-178-WT-4	0.0004093	0.0007832	0.01648
s	Aeromonas veronii	0.0003983	0.01344	0.004109
s	Thermoanaerobacterales bacterium SK-G1	0.000397	8.11E-05	0.02351
s	Peptacetobacter hominis	0.0003922	0.0001192	0.0438
s	Escherichia sp. R16	0.0003921	0.001836	0.01246
s	uncultured Citrobacter sp.	0.0003902	0.001644	0.0001153
s	Klebsiella grimontii	0.0003896	0.001239	0.0249
s	Acetobacterium paludosum	0.0003815	0.0002084	0.03379
s	Clostridium gasigenes	0.0003797	8.93E-05	0.0003264
s	Blautia sp. MSK.20.85	0.0003787	0.0001718	0.04042
s	unclassified f Peptostreptococcaceae	0.0003775	0.0001978	0.04668
s	Erysipelotrichaceae bacterium CAG:64	0.0003704	0.000295	0.02739
s	Escherichia sp. KCJ4928	0.0003656	0.001587	0.02014
s	Ferrimonas senticii	0.000365	0.001767	0.004404
s	Streptococcus sinensis	0.000363	0.0004602	0.0002624
s	mixed culture bacterium AM gF1DD01 12	0.0003611	0.00203	0.001535
s	Streptococcus sp. E24	0.0003499	0.0003443	0.0215
s	Escherichia sp. E2661	0.0003462	0.001768	0.002985
s	Pontibacillus litoralis	0.0003409	0.0002275	0.04146
s	Streptococcus sp. NM	0.0003385	0.0004042	0.004242
s	Escherichia marmotae	0.0003372	0.001968	0.001118
s	Burkholderia pseudomallei	0.0003364	0.0002057	0.04258
s	Clostridiaceae bacterium WCA-383-APC-5E	0.0003338	0.0001403	0.0204
s	unclassified g Plesiomonas	0.0003335	0.01278	0.0001556
s	Rhodococcus qingshengii	0.000326	0.001297	0.001663
s	Bacillus atrophaeus	0.0003179	0.0001077	0.007443
s	Streptococcus sp. oral taxon 071	0.0003131	0.0005015	0.01628
s	Prevotella sp. CAG:474	0.0003118	0.001589	0.01766
s	Streptococcus sp. HMSC063B03	0.0003109	0.0004855	0.002569
s	Clostridium sp. BL-8	0.0003107	0.0001513	0.002567
s	Streptococcus sp. C17	0.0003027	0.0002949	0.001154
s	Citrobacter braakii	0.0003009	0.0008446	0.0005085
s	unclassified g Morganella f Morganellace	0.0003005	0.003377	6.86E-05
s	unclassified g Drancourtella	0.0002994	0.000214	0.02437

s	Streptomyces sp. WAC01526	0.0002993	0.001697	0.00121
s	Hungateiclostridiaceae bacterium KB18	0.000299	0.0003347	0.01846
s	Coprobacillus sp. AF13-25	0.0002974	0.0001381	0.03743
s	Dysgonamonadaceae bacterium	0.0002838	1.54E-05	0.003584
s	Citrobacter amalonaticus	0.0002838	0.0005821	0.01568
s	Clostridium sp. DSM 8431	0.0002831	0.0002163	0.01972
s	Streptococcus sp. 400 SSPC	0.0002805	0.0001869	0.005582
s	Firmicutes bacterium OM04-13BH	0.0002779	0.0003673	0.01531
s	Escherichia sp. CR1	0.0002713	0.001669	0.001593
s	Richelia sp. SL 2 1	0.0002712	0	0.008819
s	Streptococcus sp. Marseille-P6264	0.0002656	0.000368	0.003174
s	Peptoniphilus lacrimalis	0.0002648	0.0002406	0.001232
s	Oleibacter marinus	0.0002631	0.0006661	0.003614
s	Coprobacillus sp. AF17-17AC	0.000263	0.0001105	0.02824
s	Clostridium septicum	0.0002621	0.0001325	0.004682
s	Streptococcus sp. HMSC066E07	0.0002599	8.81E-05	0.007844
s	Enterobacter soli	0.0002578	0.0007572	0.0002706
s	Sporomusa termitida	0.0002491	8.66E-05	0.01162
s	Streptococcus sp. HMSC034E03	0.0002483	0.0002321	0.00223
s	Cronobacter malonaticus	0.0002468	0.001898	7.11E-05
s	uncultured Desulfovibrio sp.	0.0002456	0.0005331	0.0336
s	Staphylococcus equorum	0.0002425	0.001084	0.004134
s	Clostridium cochlearium	0.0002361	0.0001355	0.02471
s	Salmonella bongori	0.0002348	0.0008333	0.0001829
s	Prevotella fusca	0.0002341	0.0002978	0.02264
s	Streptococcus sp. HMSC070B10	0.0002314	0.0002935	0.02051
s	Tumebacillus permanentifrigoris	0.0002294	0	0.03398
s	Streptococcus sp. HMSC062D07	0.0002276	0.0001662	0.003857
s	Streptococcus sp. CECT 9732	0.0002249	0.0005403	0.01581
s	Streptococcus sp. Marseille-P7376	0.0002229	0.0002675	0.002986
s	Aeromonas sp. 8C	0.0002223	0.001964	0.001572
s	Clostridium sp. C8	0.000222	0.0001014	0.02844
s	Butyrivibrio sp. ob235	0.0002212	0.0001195	0.01041
s	Prevotella sp. PTAC	0.0002201	0.0013	0.004894
s	Streptococcus sp. HMSC067H01	0.000215	0.0002007	0.005416
s	Lactobacillus sp. JM1	0.0002096	7.50E-06	0.0466
s	Roseobacter sp. MED193	0.0002076	0.000131	0.005611
s	Streptococcus sp. F0441	0.0002057	0.0003024	0.0006184
s	Streptococcus sp. HMSC34B10	0.0002056	0.0001302	0.01374
s	Escherichia sp. MR	0.0002054	0.001071	0.006626
s	Actinomyces sp. oral taxon 181	0.0002051	7.56E-05	0.02022
s	Enterobacteriaceae bacterium RIT714	0.0002026	0.0009875	0.002377
s	Citrobacter sp. DNRA3	0.0002018	0.0001703	0.03108
s	Plesiomonas shigelloides	0.0001999	0.02097	1.37E-05
s	Enterobacteriaceae bacterium RIT693	0.0001973	0.0007061	0.00227
s	Rothia dentocariosa	0.0001958	4.56E-05	0.001387
s	Leclercia adecarboxylata	0.0001946	0.000584	0.03434
s	Butyrivibrio sp. AE3009	0.0001919	0.0001009	0.0382
s	Klebsiella sp. OBRC7	0.0001909	0.000772	0.0004926
s	Clostridium acetobutylicum	0.0001904	3.29E-05	0.003799
s	Klebsiella sp. HMSC22F09	0.0001894	0.0007541	0.0006738
s	Gemella haemolysans	0.0001888	0.0001865	0.02699
s	Prevotella sp. P5-126	0.0001886	0.0009148	0.02689
s	Enterobacter sp. M4-VN	0.0001865	0.0002133	0.006376
s	Enterobacter sp. 638	0.0001863	0.0008986	0.003558
s	Bacteroides sp. PHL 2737	0.0001782	0.0002774	0.02215
s	Enterococcus sp.	0.0001766	0.0001799	0.02832
s	Corynebacterium diphtheriae	0.0001766	1.70E-05	0.03906
s	Enterobacter sp. NFIX59	0.0001761	0.0005127	0.01336

s	Lactococcus sp.	0.0001749	0.0002995	0.03858
s	Enterobacter huaxiensis	0.0001718	0.0004123	0.01925
s	Streptococcus sp. CCUG 49591	0.0001716	0.0001242	0.002084
s	Pseudodesulfovibrio sp. zrk46	0.0001706	3.02E-05	0.0358
s	Streptococcus sp. HMSC071H03	0.0001697	3.34E-05	0.002665
s	Rhizobium sp. IRBG74	0.0001697	0.0008579	0.004114
s	Streptococcus sp. M143	0.0001692	0.0001596	0.01488
s	Enterobacter mori	0.0001688	0.0004316	0.0009951
s	Shimwellia blattae	0.0001656	0.0006745	0.0001859
s	Streptococcus sp. KR	0.0001642	0.0001993	0.0005593
s	Streptococcus sp. HPH0090	0.0001588	0.0001221	0.006259
s	Enterobacter cloacae complex sp. CH23B	0.0001562	0.0005513	0.001532
s	Raoultella sp. 10-1	0.0001557	0.0006045	0.0001225
s	Labilibacter marinus	0.0001555	2.62E-05	0.02233
s	Corynebacterium sp. HMSC069E04	0.000155	5.98E-05	0.002947
s	Streptococcus massiliensis	0.0001543	9.45E-05	0.02744
s	Streptococcus peroris	0.0001527	0.0001136	0.003434
s	bacterium D16-54	0.0001505	3.94E-05	0.03689
s	Prevotella sp. oral taxon 820	0.0001485	0.0003986	0.03029
s	Izhakiella sp. KSNA2	0.0001459	0.0006002	0.0009692
s	Anaerolineaceae bacterium oral taxon 439	0.0001455	0.0002088	0.02193
s	Streptococcus sp. HMSC077D04	0.000144	0.0001292	0.002437
s	Olsenella profusa	0.0001438	9.02E-05	0.03205
s	Gottschalkia purinilytica	0.0001437	4.07E-05	0.003932
s	Buttiauxella noackiae	0.0001431	0.0006034	0.001164
s	Bacillus tropicus	0.0001414	6.48E-05	0.049
s	Streptococcus sp. DORA 10	0.00014	0.0001146	0.003814
s	Klebsiella sp. KTE92	0.0001383	0.000663	0.002869
s	Streptococcus sp. oral taxon 431	0.0001375	0.0001091	0.01698
s	Coprobacillus sp. AF13-4LB	0.0001375	6.43E-05	0.03259
s	Klebsiella sp. 4 1 44FAA	0.0001336	0.0003764	0.01295
s	Enterobacter sp. 50588862	0.0001288	0.0001922	0.01174
s	Streptococcus sp. DD04	0.0001282	0.0001963	0.02334
s	Raoultella electrica	0.0001277	0.0006058	0.001413
s	Streptococcus sp. Marseille-P7375	0.0001277	0.0001151	0.001591
s	Citrobacter sp. wls757	0.0001267	8.39E-05	0.04187
s	Natronincola ferrireducens	0.0001255	5.35E-05	0.01865
s	Streptococcus sp. Marseille-P644	0.0001217	0.0001056	0.01188
s	Schaalia hyovaginalis	0.000121	6.68E-05	0.03411
s	Pelolinea sp.	0.0001206	2.79E-05	0.04159
s	Raoultella sp. BIGb0138	0.0001194	0.0004871	0.001921
s	Enterobacter sp. CM29	0.0001188	0.0002546	0.03195
s	Clostridium peptidivorans	0.0001165	0.0001133	0.02957
s	Klebsiella sp. RIT-PI-d	0.0001156	0.0004182	0.0004898
s	Cytobacillus oceanisediminis	0.0001152	4.86E-05	0.008191
s	Clostridium sp. 2-1	0.0001148	3.45E-05	0.02812
s	Citrobacter rodentium	0.000114	0.0006581	0.0009319
s	Lysinibacillus sp. BW-2-10	0.000114	3.31E-05	0.04252
s	Desulfofarcimen acetoxidans	0.0001137	0.0001077	0.04719
s	Klebsiella variicola CAG:634	0.0001133	0.0006693	0.004052
s	Paenibacillus sp. OK003	0.0001123	0.0001323	0.01757
s	Moraxella bovoculi	0.0001122	6.46E-05	0.013
s	Paraclostridium sp. SKVG24	0.0001113	6.47E-05	0.03324
s	Klebsiella sp. X1-16S-Nf21	0.0001107	0.0003896	0.0005589
s	Clostridium sp. SYSU GA17076	0.0001086	5.93E-05	0.04111
s	Streptococcus sp. oral taxon 056	0.0001076	5.29E-05	0.007844
s	Prevotella sp. P5-108	0.0001069	0.0007505	0.02329
s	Lactococcus piscium	0.0001068	0.0001825	0.03494
s	Clostridium coskatii	0.0001048	0.0001631	0.01392

s	Streptococcus sp. CCUG 71758	0.0001044	7.52E-05	0.006919
s	unclassified g Terrisporobacter	0.0001035	4.75E-05	0.003422
s	Hypnocyclicus thermotrophus	0.0001019	0.0001539	0.03259
s	Azoarcus sp. CC-YHH838	0.0001015	0	0.03398
s	Candidatus Omnitrophica bacterium ADurb	0.0001005	6.56E-05	0.04781
s	Clostridium sp. Ade.TY	9.81E-05	5.42E-05	0.03103
s	Porphyromonas levii	9.46E-05	0.0001484	0.009462
s	TM7 phylum sp. oral taxon 348	9.42E-05	3.51E-05	0.01747
s	Streptococcus sp. SK643	9.41E-05	0.0001508	0.007055
s	Klebsiella sp. P1CD1	9.39E-05	0.0003662	0.002917
s	Lysinibacillus sp. AR18-8	9.34E-05	7.62E-05	0.04798
s	Prevotella sp. S7-1-8	9.13E-05	0.0002042	0.02643
s	Escherichia sp. MOD1-EC4550	9.02E-05	0.0004441	0.001684
s	Virgibacillus sp. 6R	9.00E-05	5.87E-05	0.04306
s	uncultured bacterium (gcode 4)	8.79E-05	7.10E-05	0.03327
s	Mixta intestinalis	8.65E-05	0	0.03398
s	Salmonella sp. NCTC 7297	8.63E-05	0.0003419	0.000473
s	Peptidiphaga gingivicola	8.62E-05	9.55E-05	0.01406
s	Streptococcus sp. BIOML-A1	8.61E-05	5.83E-05	0.04172
s	Streptococcus sp. WB01 FAA12	8.60E-05	0.0001416	0.005705
s	Streptococcus porci	8.51E-05	5.56E-05	0.02654
s	Yersinia pestis	8.50E-05	0.0006478	0.01376
s	Paenibacillus sp. LMG 31460	8.46E-05	7.24E-05	0.02683
s	Lactobacillus gastricus	8.36E-05	1.30E-05	0.01435
s	Raoultella sp. T31	8.34E-05	0.0004321	0.00143
s	Streptomyces sp. C	8.33E-05	2.83E-05	0.001239
s	Streptococcus sp. 116-D4	8.27E-05	6.50E-05	0.001484
s	Schaalia cardiffensis	8.23E-05	3.07E-05	0.0002478
s	Streptococcus sp. HMSC073F11	8.17E-05	9.55E-05	0.02377
s	Klebsiella sp.	8.17E-05	0.0003816	0.0006911
s	Streptococcus chosunense	8.10E-05	9.42E-05	0.001306
s	Klebsiella sp. AS10	8.06E-05	0.0005928	0.0001938
s	Streptococcus sp. HMSC056C01	7.96E-05	9.18E-05	0.01841
s	Escherichia sp. B1147	7.95E-05	0.0005468	0.0004168
s	Bacteroidetes bacterium RIFCSPLOWO2 1	7.90E-05	3.51E-05	0.04874
s	Serratia fonticola	7.82E-05	0.0002771	0.006589
s	Streptococcus sp. 1643	7.82E-05	8.50E-05	0.01126
s	uncultured bacterium fosmid pJB69A5	7.81E-05	0	0.03398
s	candidate division CPR1 bacterium ADurb.	7.78E-05	5.10E-06	0.02397
s	Klebsiella sp. NFIX53	7.75E-05	0.000326	0.006226
s	Breznakia blatticola	7.47E-05	2.61E-05	0.000402
s	Roseateles terrae	7.40E-05	0	0.03398
s	Desulfobaculum xiamenense	7.31E-05	1.41E-06	0.00427
s	Pantoea ananatis	7.22E-05	0.0003313	3.39E-05
s	Enterobacteriaceae bacterium	7.20E-05	0.0001638	0.01141
s	Candidatus Symbiobacter mobilis	7.10E-05	0	0.03398
s	Streptococcus sp. NLAE-zl-C503	6.90E-05	0.0001172	0.00789
s	uncultured bacterium Contig359	6.81E-05	0.0007549	0.013
s	Ramlibacter sp. B156	6.67E-05	0	0.03398
s	Streptococcus sp. LQJ-218	6.57E-05	7.10E-05	0.03653
s	Streptococcus ursoris	6.53E-05	1.79E-05	0.01995
s	Granulicatella sp. 572.rep1 STHE	6.47E-05	2.62E-05	0.03501
s	Faecalicatena sp. AGMB00832	6.45E-05	2.59E-05	0.01488
s	Escherichia sp. HH26CH	6.44E-05	0.0003494	0.01097
s	Streptococcus halitosis	6.42E-05	4.71E-05	0.002856
s	Cronobacter turicensis	6.41E-05	0.0003823	0.0006368
s	Alkalihalobacillus akibai	6.40E-05	1.32E-05	0.04296
s	Candidatus Cloacimonetes bacterium	6.39E-05	0.0001329	0.01868
s	Novosphingobium naphthalenivorans	6.31E-05	0.0003368	0.002304

s	Lysinibacillus antri	6.27E-05	1.12E-06	0.01397
s	TM7 phylum sp. oral taxon 346	6.21E-05	4.34E-05	0.000936
s	Aeromonas hydrophila	6.19E-05	0.00293	0.003358
s	Lactobacillus brevis	6.16E-05	4.22E-05	0.03608
s	Bacillus sp. CGMCC 1.16541	6.06E-05	4.34E-06	0.008334
s	Bacillus sp. 1NLA3E	6.04E-05	1.07E-05	0.03703
s	unclassified g Kosakonia	6.03E-05	0.0001254	0.04848
s	Streptococcus sp. bf 0095	6.01E-05	3.78E-05	0.02177
s	Corynebacterium propinquum	6.00E-05	2.93E-05	0.02886
s	bacterium P3	5.99E-05	0.0001995	0.0464
s	Arthrobacter sp. 4R501	5.96E-05	0.0002921	0.000338
s	Burkholderia dolosa	5.89E-05	0.0001067	0.04597
s	Psychrosphaera saromensis	5.89E-05	0.0002435	0.02123
s	Butyrivibrio sp. CB08	5.88E-05	0.0001422	0.04931
s	Slackia sp. CM382	5.82E-05	7.10E-06	0.003013
s	Streptococcus sp. SK140	5.77E-05	5.64E-05	0.002789
s	Streptococcus sp. HMSC067A03	5.75E-05	1.72E-05	0.01214
s	Ruminococcaceae bacterium AB4001	5.56E-05	0.0001382	0.03999
s	Klebsiella sp. D5A	5.46E-05	0.0003168	0.000633
s	Microbacterium liquefaciens	5.46E-05	1.41E-05	0.01288
s	Staphylococcus nepalensis	5.44E-05	2.26E-07	0.01397
s	Paenibacillus taichungensis	5.43E-05	2.02E-05	0.02748
s	Lactobacillus sp. 33-1	5.42E-05	0	0.008819
s	Enterobacter sp. kpr-6	5.30E-05	0.000119	0.03835
s	Fibrobacter sp. UWB4	5.18E-05	3.19E-05	0.02739
s	Bacteroidetes bacterium ADurb.Bin013	5.17E-05	0	0.008819
s	Aliivibrio sifiae	5.15E-05	6.54E-07	0.04766
s	Staphylococcus hominis	5.08E-05	1.02E-05	0.04619
s	Gordonibacter sp. Marseille-P4307	5.02E-05	1.77E-05	0.0268
s	Bifidobacterium cuniculi	4.99E-05	5.31E-05	0.03874
s	Pseudochelatococcus lubricantis	4.99E-05	0	0.03398
s	Enterobacter sp. NFIX45	4.95E-05	0.0001184	0.001886
s	Prevotella scopos	4.94E-05	0.0002681	0.01049
s	Streptococcus sp. oral taxon 064	4.93E-05	3.71E-05	0.01652
s	Rhodoferax koreense	4.92E-05	5.41E-07	0.04766
s	Bacteroidales bacterium 36-12	4.86E-05	0.001036	0.02521
s	Chromobacterium haemolyticum	4.85E-05	1.15E-06	0.04766
s	Bacillus sp. USDA818B3 A	4.83E-05	1.34E-05	0.0305
s	Agromyces sp. CF514	4.83E-05	0	0.03398
s	Gleimia hominis	4.82E-05	1.13E-05	0.004446
s	Acidovorax oryzae	4.75E-05	0	0.03398
s	Staphylococcus sp. 8AQ	4.73E-05	3.13E-05	0.003047
s	Escherichia sp. MOD1-EC7003	4.66E-05	0.0006004	0.03906
s	Gottschalkia acidurici	4.64E-05	2.34E-05	0.02907
s	Microbacterium sp.	4.62E-05	3.32E-06	0.00427
s	Citrobacter sp. wls830	4.61E-05	0.0001565	0.007538
s	Veillonella sp. 3310	4.59E-05	8.06E-06	0.01594
s	Ferruginibacter sp. BO-59	4.56E-05	0	0.03398
s	Staphylococcus capitis	4.55E-05	4.23E-05	0.03581
s	Enterobacter sp. BIDMC100	4.53E-05	0.0002089	0.04322
s	Caldiserica bacterium	4.53E-05	2.83E-05	0.03617
s	Tessaracoccus bendigoensis	4.42E-05	1.87E-05	0.008913
s	Capnocytophaga sp. oral taxon 324	4.33E-05	1.02E-06	0.01397
s	Alkalihalobacillus hwajinpoensis	4.31E-05	6.11E-06	0.01527
s	Citrobacter sp. wls714	4.30E-05	2.02E-06	0.0457
s	Peptoniphilus vaginalis	4.28E-05	4.05E-06	0.0429
s	Nitrosomonas ureae	4.24E-05	0.0001827	0.04058
s	Rothia nasimurium	4.22E-05	1.62E-06	0.007425
s	Thiotrichales bacterium SG8 50	4.22E-05	2.87E-06	0.0457

s	Pandoraea pnomenusa	4.18E-05	1.18E-07	0.04766
s	Actinomyces sp. Chiba101	4.12E-05	1.19E-05	0.004234
s	Actinomyces denticolens	4.03E-05	1.43E-05	0.03556
s	Cohaesibacter sp. ES.047	4.00E-05	2.05E-05	0.01324
s	Lactobacillus kefiranofaciens	3.99E-05	2.69E-05	0.03246
s	Geobacter sulfurreducens	3.96E-05	7.80E-05	0.03938
s	Bacillus salacetis	3.95E-05	1.02E-05	0.04449
s	Paenibacillus aquistagni	3.95E-05	1.03E-05	0.01691
s	Burkholderia sp. BDU5	3.91E-05	0	0.03398
s	Streptococcus sp. UMB1385	3.83E-05	2.06E-05	0.02199
s	Paenibacillus sp. 7523-1	3.80E-05	0	0.002321
s	Lactobacillus hokkaidonensis	3.73E-05	5.42E-07	0.04766
s	Rothia sp. HMSC067H10	3.73E-05	7.74E-06	0.0001581
s	Muricauda sp.	3.70E-05	4.06E-06	0.01487
s	uncultured murine large bowel bacterium E	3.69E-05	1.53E-05	0.0319
s	Psychrobacter sp. JB193	3.66E-05	0	0.03398
s	Rhodoplanes sp. Z2-YC6860	3.63E-05	3.77E-07	0.01397
s	unclassified o Micrococcales	3.63E-05	3.41E-05	0.04619
s	Bacillus sp. URHB0009	3.62E-05	1.30E-05	0.01082
s	Anaerosalibacter bizertensis	3.61E-05	4.20E-05	0.04525
s	uncultured Lactobacillus sp.	3.59E-05	2.92E-05	0.01088
s	unclassified g Paucibacter	3.58E-05	0	0.008819
s	Marininema halotolerans	3.58E-05	0	0.03398
s	Candidatus Scalindua rubra	3.58E-05	6.10E-07	0.0457
s	Plantibacter flavus	3.57E-05	7.73E-06	0.04215
s	Streptococcus sp. GMD6S	3.56E-05	3.81E-05	0.01676
s	Paenibacillus sp. EKM205P	3.53E-05	0	0.008819
s	unclassified g Shewanella	3.48E-05	6.77E-06	0.007377
s	Ancylomarina sp. 16SWW S1-10-2	3.46E-05	1.45E-05	0.04461
s	Desulfotignum phosphitoxidans	3.45E-05	0	0.03398
s	Enterococcus sp. JM4C	3.43E-05	1.38E-05	0.03308
s	Kurthia gibsonii	3.39E-05	6.18E-06	0.04035
s	Collinsella sp. AF18-8LB	3.38E-05	0.0001023	0.03665
s	Gracilibacillus kekensis	3.35E-05	7.43E-07	0.001015
s	Paenisporosarcina sp. TG-14	3.34E-05	1.92E-05	0.0268
s	Rothia sp. HMSC065G12	3.31E-05	1.19E-05	0.03951
s	Burkholderia sp. TJI49	3.23E-05	0	0.03398
s	Corynebacterium variabile	3.21E-05	9.21E-06	0.01051
s	Escherichia sp. E3659	3.19E-05	0.0001997	0.0008668
s	Paraburkholderia kirstenboschensis	3.18E-05	7.44E-06	0.03286
s	Flavobacterium sp. CLA17	3.13E-05	6.76E-06	0.0229
s	Chryseobacterium sp. RJ-7-14	3.12E-05	0	0.03398
s	Candidatus Blackburnbacteria bacterium	3.08E-05	0	0.03398
s	Chromohalobacter salexigens	3.04E-05	0	0.008819
s	Labedella gwakjiensis	3.01E-05	0	0.008819
s	Flavobacterium cupreum	3.01E-05	0	0.03398
s	Streptococcus sp. GMD4S	2.99E-05	2.64E-05	0.0249
s	Propionimicrobium sp. Marseille-P3275	2.99E-05	5.74E-05	0.04935
s	Stenotrophomonas sp. DAIF1	2.97E-05	0	0.03398
s	Enterobacter cloacae complex sp. ECNIH1	2.96E-05	1.64E-06	0.01397
s	Chryseobacterium culicis	2.94E-05	0	0.03398
s	Streptococcus sp. GMD1S	2.93E-05	2.51E-05	0.008605
s	Glaciibacter superstes	2.92E-05	5.38E-06	0.007435
s	Bordetella ansorpii	2.91E-05	1.83E-06	0.0457
s	Rothia sp. HMSC066H02	2.86E-05	9.83E-06	0.03343
s	Methylicorpusculum oleiharenae	2.83E-05	0	0.03398
s	Actinomyces ruminicola	2.83E-05	7.91E-06	0.02031
s	Burkholderia sp. AU17325	2.82E-05	0	0.03398
s	Pigmentiphaga sp. NML080357	2.81E-05	0	0.03398

s	Arachidicoccus sp. KIS59-12	2.76E-05	0	0.03398
s	Herbaspirillum sp. VT-16-41	2.74E-05	0	0.03398
s	Brachyspira intermedia	2.71E-05	4.97E-07	0.003504
s	Euryhalocaulis caribicus	2.67E-05	0	0.03398
s	Staphylococcus hyicus	2.66E-05	0	0.03398
s	Janthinobacterium sp. 1 2014MBL MicDiv	2.65E-05	0	0.008819
s	Paraburkholderia aromaticivorans	2.64E-05	0	0.03398
s	Pasteurellaceae bacterium	2.62E-05	0	0.03398
s	Streptococcus sp. SR1	2.61E-05	6.57E-05	0.02948
s	Klebsiella sp. JL973	2.61E-05	8.27E-05	0.01961
s	Thermorudis sp.	2.61E-05	0	0.03398
s	Brachyspira sp. G79	2.58E-05	0	0.03398
s	Bifidobacteriaceae bacterium NR015	2.56E-05	0	0.008819
s	Staphylococcus succinus	2.55E-05	0.000183	0.006443
s	Corynebacterium lowii	2.52E-05	1.47E-06	0.0435
s	Streptococcus macacae	2.49E-05	4.93E-06	0.002153
s	Carnobacterium sp. PL12RED10	2.48E-05	1.46E-06	0.03378
s	Actinomyces provencensis	2.48E-05	0	0.008819
s	Acinetobacter johnsonii	2.48E-05	5.16E-06	0.004504
s	Pseudocitrobacter sp. RIT 415	2.46E-05	5.38E-06	0.01683
s	Streptococcus sp. HMSC071D03	2.42E-05	2.00E-05	0.0005938
s	Anaerovibrio sp.	2.40E-05	8.00E-07	0.01287
s	Duganella sacchari	2.37E-05	1.41E-06	0.04766
s	Burkholderiales bacterium 12-64-5	2.31E-05	0	0.03398
s	Variovorax sp. RO1	2.31E-05	0	0.03398
s	Microbacterium sp. No. 7	2.28E-05	1.19E-06	0.0457
s	Coprobacillus sp. AF18-15LB	2.24E-05	9.94E-06	0.04402
s	Burkholderia sp. JKS000303	2.23E-05	0	0.03398
s	Candidatus Dorea massiliensis	2.23E-05	6.28E-06	0.009469
s	Granulicoccus phenolivorans	2.23E-05	8.20E-06	0.02293
s	Rothia sp. Olga	2.22E-05	7.96E-06	0.005597
s	Calothrix sp. HK-06	2.15E-05	3.73E-06	0.04035
s	Paenibacillus sp. VT-16-81	2.14E-05	4.03E-05	0.04894
s	Streptococcus sp. CCH8-G7	2.14E-05	0	9.97E-06
s	Methylobacterium bullatum	2.12E-05	0	0.03398
s	Lysinibacillus sp.	2.12E-05	1.67E-06	0.01594
s	Azospirillum sp. B21	2.11E-05	0	0.03398
s	Haliangium ochraceum	2.08E-05	2.38E-05	0.03703
s	unclassified g Acidovorax	2.07E-05	0	0.03398
s	Candidatus Saccharibacteria oral taxon TM	2.06E-05	1.04E-05	0.01579
s	Erysipelothrix sp. D14 1188-2-1-2	2.01E-05	5.08E-06	0.03214
s	Yersinia pseudotuberculosis	1.99E-05	0	0.03398
s	unclassified f Micrococcaceae	1.97E-05	0	0.008819
s	Cronobacter universalis	1.96E-05	7.90E-05	0.009204
s	Bradyrhizobium centrolobii	1.94E-05	8.50E-05	0.04219
s	Piscicoccus intestinalis	1.94E-05	1.15E-05	0.02607
s	Chlorobi bacterium	1.90E-05	0.0001235	0.0212
s	Streptococcus sp. FDAARGOS 146	1.90E-05	2.26E-06	0.000127
s	unclassified f Promicromonosporaceae	1.90E-05	4.41E-06	0.04581
s	Enterobacter sp. 10-1	1.88E-05	0.0001348	0.001937
s	Nostocales cyanobacterium HT-58-2	1.83E-05	3.18E-07	0.04766
s	Microgenomates group bacterium GW2011	1.82E-05	5.01E-06	0.02941
s	Candidatus Kentron sp. H	1.79E-05	0	0.03398
s	Ktedonobacter sp.	1.78E-05	3.29E-06	0.02609
s	Dermacoccus nishinomiyaensis	1.76E-05	0	0.008819
s	Parvibaculum indicum	1.76E-05	0	0.03398
s	Oceanobacillus profundus	1.75E-05	8.65E-07	0.01397
s	uncultured bacterium Contig1450	1.75E-05	0	0.03398
s	bacterium JKG1	1.75E-05	0	0.008819

s	Pedobacter sp. RP-3-21	1.72E-05	0	0.03398
s	Proteus sp. HMSC10D02	1.71E-05	3.45E-05	0.04493
s	Megasphaera sp. BIOML-A2	1.71E-05	0	0.008819
s	Brenneria goodwinii	1.69E-05	7.23E-08	0.01311
s	Shewanella pealeana	1.68E-05	0	0.03398
s	Streptococcus sp. HMSC034B04	1.67E-05	4.88E-06	0.00164
s	Treponema sp. CETP13	1.67E-05	1.51E-06	0.01397
s	Leuconostoc kimchii	1.67E-05	1.92E-05	0.04672
s	Ottowia sp. GY511	1.66E-05	0	0.03398
s	Cronobacter condimenti	1.61E-05	7.10E-05	0.0174
s	candidate division WOR-3 bacterium	1.59E-05	6.83E-05	0.03286
s	Zhouia amylolytica	1.59E-05	4.14E-07	0.01311
s	Citrobacter sp. CF971	1.58E-05	1.24E-05	0.04605
s	Clostridium sp. MF28	1.58E-05	1.79E-07	0.04766
s	Actinomyces slackii	1.56E-05	1.86E-06	0.004061
s	Candidatus Burkholderia verschuerenii	1.55E-05	1.58E-07	0.04766
s	Caldicellulosiruptor morganii	1.55E-05	1.59E-06	0.0435
s	Spirosoma sp. KCTC 42546	1.55E-05	2.75E-06	0.04799
s	Halieaceae bacterium	1.54E-05	4.76E-07	0.003744
s	Anoxybacillus vitaminiphilus	1.53E-05	1.26E-06	0.04799
s	Actinomyces sp. HMSC075B09	1.52E-05	4.68E-06	0.01758
s	Paracoccus luteus	1.49E-05	0	0.03398
s	Bacillus sp. E(2018)	1.49E-05	0	0.03398
s	Lactobacillus apodemi	1.47E-05	0	0.008819
s	Enterobacter cloacae complex sp. ECNIH14	1.47E-05	1.51E-05	0.04596
s	Candidatus Anoxychlamydiales bacterium	1.47E-05	4.72E-07	0.04766
s	uncultured bacterium Contig1480	1.47E-05	3.77E-05	0.00449
s	Shigella sp. MO17	1.45E-05	0	0.03398
s	Bacillus sp. 3-2-2	1.44E-05	7.71E-06	0.04975
s	Thiocapsa sp. WGA12-6	1.41E-05	0	0.03398
s	Giesbergeria anulus	1.40E-05	0	0.03398
s	Comamonas sp. AG1104	1.40E-05	0	0.03398
s	Achromobacter sp. 2789STDY5608628	1.40E-05	0	0.03398
s	Leucobacter sp. wl10	1.37E-05	0	0.008819
s	Kosakonia arachidis	1.34E-05	6.25E-05	0.001051
s	Paenibacillus sp. N4	1.33E-05	0	0.03398
s	Segetibacter sp. 3557 3	1.32E-05	2.68E-05	0.03459
s	Bacillus ndiopicus	1.31E-05	7.85E-06	0.02212
s	Lachnospiraceae bacterium AM25-39	1.31E-05	9.96E-06	0.01321
s	Citrobacter sp. wls708	1.30E-05	1.65E-06	0.03378
s	Timonella senegalensis	1.30E-05	0	0.03398
s	Bacteroidetes bacterium 37-13	1.29E-05	0	0.03398
s	Microgenomates group bacterium RBG 19F	1.28E-05	0	0.03398
s	Fulvivirga imtechensis	1.27E-05	0	0.03398
s	Escherichia sp. TW14182	1.26E-05	6.51E-05	0.02162
s	Leclercia sp. 29361	1.26E-05	0	0.008819
s	Propioniciclava sp. HDW11	1.25E-05	0	0.008819
s	Ignatzschineria indica	1.25E-05	5.17E-06	0.04199
s	Streptococcus sp. HMSC072D07	1.24E-05	2.17E-05	0.04256
s	candidate division CPR3 bacterium GW201	1.23E-05	0	0.03398
s	Hafnia paralvei	1.22E-05	5.70E-05	0.009259
s	Lysinibacillus mangiferihumi	1.21E-05	6.38E-06	0.03487
s	Bifidobacterium sp. UTCIF-39	1.21E-05	1.23E-05	0.03372
s	Acidovorax sp. T1	1.21E-05	0	0.03398
s	Formosa algae	1.20E-05	4.06E-07	0.04766
s	Acinetobacter parvus	1.20E-05	0	0.03398
s	Acidovorax anthurii	1.20E-05	0	0.03398
s	Jonesia denitrificans	1.19E-05	0	0.002321
s	Rothia terrae	1.18E-05	0	0.03398

s	Cupriavidus sp. SK-3	1.18E-05	0	0.03398
s	Pseudomonas linyingensis	1.17E-05	0	0.03398
s	Phormidesmis priestleyi	1.17E-05	0	0.03398
s	Vibrio mimicus	1.16E-05	9.83E-06	0.006479
s	Variovorax sp. 553	1.14E-05	0	0.03398
s	unclassified g Helicobacter	1.14E-05	4.08E-07	0.04766
s	Methylophilus medardicus	1.14E-05	0	0.03398
s	Planomicrobium sp. CPCC 101110	1.13E-05	8.59E-06	0.03247
s	Pseudoclavibacter sp. RFBG4	1.13E-05	0	0.008819
s	Pantoea eucrina	1.13E-05	0	0.03398
s	Paraburkholderia dilworthii	1.12E-05	0	0.03398
s	Pseudomonas sp. 91RF	1.12E-05	0	0.03398
s	Chitinophaga sp. Ak27	1.11E-05	0	0.03398
s	Microbacterium radiodurans	1.10E-05	0	0.03398
s	Olavius sp. associated proteobacterium Del	1.10E-05	0	0.03398
s	Acidovorax konjaci	1.09E-05	0	0.03398
s	Klebsiella sp. H-Nf2	1.09E-05	0.0001704	0.01956
s	Leclercia sp. LSNIH1	1.08E-05	0	0.03398
s	Apibacter sp. HY039	1.07E-05	1.13E-05	0.04035
s	Janthinobacterium lividum	1.06E-05	9.05E-07	0.01512
s	Microbacterium sp. Yaish 1	1.06E-05	0	0.008819
s	Vibrio vulnificus	1.05E-05	0.0001403	0.002181
s	Rhizobium sp. NFR03	1.05E-05	0	0.03398
s	Candidatus Brocadiaceae bacterium B188	1.04E-05	0	0.03398
s	Cytobacillus gottheilii	1.04E-05	6.45E-06	0.02019
s	Pseudomonas sp. Leaf127	1.04E-05	0	0.03398
s	Bacillus sp. J33	1.01E-05	1.21E-06	0.00427
s	Verrucosispora sp. CWR15	9.96E-06	0	0.008819
s	Mangrovibacterium sp. BM 7	9.88E-06	0.0001102	0.0124
s	Curtobacterium sp. Leaf261	9.66E-06	0	0.03398
s	Acidovorax delafieldii	9.62E-06	0	0.03398
s	Caldilinea aerophila	9.59E-06	0	0.03398
s	Microbacterium lindanitolerans	9.57E-06	0	0.03398
s	Mesocricetibacter intestinalis	9.54E-06	4.00E-06	0.01865
s	Pseudomonas sp. AP42	9.45E-06	0	0.03398
s	Celeribacter persicus	9.44E-06	0	0.008819
s	candidate division TA06 bacterium ADurb.	9.42E-06	1.41E-06	0.01582
s	Flavobacterium sp. xlx-214	9.40E-06	3.67E-06	0.0177
s	Amnibacterium sp. M8JJ-5	9.24E-06	5.02E-07	0.04766
s	Xenorhabdus bovienii	9.15E-06	8.46E-05	0.000274
s	Bacillus alkalitolerans	9.11E-06	0	0.03398
s	Candidatus Omnitrophica bacterium CG23	9.00E-06	0	0.03398
s	Mesorhizobium sp. B2-3-2	8.80E-06	0	0.03398
s	Halomonas sp. THAF5a	8.66E-06	1.42E-06	0.01582
s	endosymbiont of Euscepes postfasciatus	8.61E-06	0	0.03398
s	Alphaproteobacteria bacterium CG 4 9 14	8.57E-06	0	0.03398
s	Deltaproteobacteria bacterium HGW-Deltapı	8.54E-06	0	0.008819
s	Promicromonospora sp. AC04	8.51E-06	8.35E-07	0.01582
s	Paenibacillus popilliae	8.49E-06	3.38E-05	0.04824
s	Porphyromonas somerae	8.39E-06	4.93E-05	0.01704
s	Acidovorax sp. 62	8.19E-06	0	0.008819
s	Kurthia sp. 11kri321	8.16E-06	6.58E-07	0.04766
s	Lysinimicrobium gelatinilyticum	8.14E-06	0	0.03398
s	Leptospira levettii	7.96E-06	0	0.008819
s	Allochromatium palmeri	7.94E-06	0	0.03398
s	Streptomyces sp. Z38	7.86E-06	0	0.03398
s	Pseudoflavonifractor sp. BIOML-A3	7.81E-06	0	0.03398
s	Bacteroidetes bacterium CG23 combo of C	7.79E-06	0	0.03398
s	Opitutaceae bacterium	7.76E-06	6.97E-07	0.01487

s	Propionibacterium sp. KPL2005	7.72E-06	6.90E-07	0.04766
s	Scardovia inopinata	7.67E-06	0	0.03398
s	Akkermansia glycaniphila	7.67E-06	8.27E-05	0.04415
s	Brachyspira suanatina	7.44E-06	0.0001389	0.03788
s	Microbacterium gubbeenense	7.38E-06	3.10E-07	0.04766
s	Salinicola sp. MH3R3-1	7.25E-06	0	0.03398
s	Nocardioides alpinus	7.22E-06	0	0.008819
s	Bacillus sp. B1-WWTP-T-0.5-Post-4	7.13E-06	0	0.03398
s	Candidimonas bauzanensis	7.13E-06	5.67E-07	0.01397
s	Arthrobacter sp. AQ5-05	7.05E-06	0	0.008819
s	Paenibacillus spiritus	6.76E-06	7.47E-05	0.04185
s	Rothia sp. HMSC064D08	6.75E-06	0	0.03398
s	Idiomarina ramblicola	6.70E-06	0	0.03398
s	Desulfonauticus sp. 38 4375	6.48E-06	0	0.03398
s	Lactococcus sp. dk101	6.41E-06	0	0.03398
s	Streptomyces turgidiscabies	6.40E-06	0	0.03398
s	Blastococcus sp. Marseille-P5729	6.30E-06	0	0.03398
s	Rufibacter sp. R-22-1c-1	6.13E-06	0	0.03398
s	Paenibacillus sp. UNC217MF	6.03E-06	0	0.03398
s	Bacillaceae bacterium MTCC 10057	6.02E-06	0	0.03398
s	Microbacteriaceae bacterium AFLP121	5.96E-06	0	0.03398
s	uncultured bacterium Contig14	5.83E-06	0	0.03398
s	Salinarimonadaceae bacterium HL-109	5.81E-06	0	0.03398
s	Bifidobacterium sp. wkB338	5.71E-06	0	0.03398
s	Oxalobacteraceae bacterium AB 14	5.58E-06	0	0.03398
s	Dethiosulfovibrio salsuginis	5.48E-06	2.33E-05	0.0459
s	Microbacterium sp. AG1240	5.39E-06	0	0.03398
s	Escherichia sp. MOD1-EC5189	5.28E-06	0	0.03398
s	Sinomonas susongensis	5.25E-06	0	0.03398
s	Propionibacteriaceae bacterium P6A17	5.22E-06	0	0.03398
s	Bacillus freudenreichii	5.20E-06	0	0.03398
s	Corynebacterium minutissimum	5.19E-06	0	0.03398
s	Nocardiopsis chromatogenes	5.14E-06	0	0.03398
s	Corynebacterium suranareeae	5.05E-06	0	0.03398
s	Parcubacteria group bacterium Gr01-1014 3	4.86E-06	0	0.03398
s	Fusobacterium massiliense	4.81E-06	0.0001474	0.0291
s	Microbacterium sp. 292MF	4.80E-06	0	0.03398
s	Leptolyngbyaceae cyanobacterium SM2 5 2	4.71E-06	0	0.03398
s	Bifidobacterium choerinum	4.70E-06	0	0.03398
s	Nocardioides caeni	4.63E-06	0	0.03398
s	Arthrobacter sp. H14	4.59E-06	1.99E-07	0.04766
s	Arcobacter cloacae	4.56E-06	0	0.03398
s	Gilliamella intestini	4.51E-06	0	0.03398
s	Thiobacillus sp.	4.19E-06	0	0.03398
s	Agrococcus baldri	4.11E-06	0	0.03398
s	Klebsiella sp. J-Nf11	4.09E-06	4.17E-05	0.04073
s	Azotobacter chroococcum	3.96E-06	2.09E-05	0.006937
s	Chlorobium phaeovibrioides	3.88E-06	0	0.03398
s	Pleurocapsa sp. SU 196 0	3.72E-06	3.23E-07	0.003999
s	Bacillus mangrovi	3.64E-06	0	0.03398
s	Melghirimyces algeriensis	3.62E-06	1.92E-05	0.04073
s	Curtobacterium sp. MCBA15 013	3.56E-06	0	0.03398
s	Novosphingobium barchaimii	3.54E-06	0	0.03398
s	Halomonas sp. JB37	3.51E-06	0.0001146	0.03362
s	Erysipelotrichales bacterium	3.40E-06	0	0.03398
s	Planctomycetia bacterium TMED53	3.37E-06	0	0.03398
s	Sporolituus thermophilus	3.13E-06	4.18E-05	0.0291
s	Campylobacter showae	2.98E-06	4.33E-05	0.03953
s	Enterococcaceae bacterium	2.93E-06	3.64E-06	0.03348

s	Eggerthellaceae bacterium zg-997	2.80E-06	5.35E-05	0.04323
s	Burkholderia sp. USM B20	2.72E-06	0	0.03398
s	Lentimicrobium sp. S6	2.65E-06	0	0.03398
s	Pedobacter sp. RP-3-22	2.56E-06	0	0.03398
s	Thermosporothrix sp. COM3	2.55E-06	0	0.03398
s	Subtercola vilae	2.42E-06	0	0.03398
s	Acidovorax sp. SCN 68-22	2.26E-06	2.15E-05	0.04889
s	Nocardioides sp. ZJ1313	2.20E-06	0	0.03398
s	Bacteriovorax sp. Seq25 V	2.10E-06	0	0.03398
s	Planomicrobium sp. YIM 101495	2.08E-06	0	0.03398
s	Candidatus Gastranaerophilales bacterium H	2.07E-06	0.0002096	0.03801
s	Prevotella sp. oral taxon 299	2.06E-06	8.81E-05	0.04619
s	Runella zeae	1.99E-06	2.74E-05	0.03157
s	unclassified g Proteus f Morganellaceae	1.98E-06	0.00021	0.007554
s	Akkermansia sp. BIOML-A39	1.90E-06	0	0.03398
s	Halomonas huangheensis	1.84E-06	0	0.03398
s	Lactobacillus malefermentans	1.40E-06	1.60E-05	0.04889
s	Candidatus Dojkabacteria bacterium	1.25E-06	9.84E-05	0.04782
s	Hydrogenispora sp.	1.07E-06	1.71E-05	0.0179
s	Caldalkalibacillus thermarum	6.22E-07	1.47E-05	0.04619
s	Aeromonas taiwanensis	4.96E-07	3.58E-05	0.04461
s	Clostridiales bacterium Marseille-P2986	4.55E-07	5.61E-05	0.04461
s	Leptolyngbyaceae cyanobacterium CCMR0(	3.92E-07	0	0.03398
s	Desulfarculus sp.	3.88E-07	1.03E-05	0.03189
s	Plesiomonas sp. ZOR0011	2.63E-07	0.0004145	0.04619
s	Acinetobacter seifertii	0	3.01E-05	0.01431
s	Bacillus sp. FJAT-44876	0	8.48E-06	0.01431
s	Polaribacter gangjinensis	0	7.81E-06	0.01431
s	Labrenzia sp. VG12	0	7.08E-05	0.01977
s	Anaerococcus sp. Marseille-P3625	0	1.00E-05	0.01977
s	Niabella ginsenosidivorans	0	9.20E-05	0.02722
s	Brevibacillus sp. SCSIO 07484	0	3.79E-05	0.02722
s	Burkholderiales bacterium 68-10	0	2.26E-05	0.02722
s	Methylotenera sp. La3113	0	2.09E-05	0.02722
s	Malonomonas rubra	0	1.59E-05	0.02722
s	Pantoea allii	0	1.31E-05	0.02722
s	Methylobacter sp. BBA5.1	0	1.06E-05	0.02722
s	Arenibacter aquaticus	0	0.0001208	0.03735
s	Sphingobacterium paucimobilis	0	7.82E-05	0.03735
s	Saccharicrinis fermentans	0	5.09E-05	0.03735
s	Streptococcus downei	0	3.50E-05	0.03735
s	Desulfuromusa sp.	0	3.39E-05	0.03735
s	Desulfobacula toluolica	0	2.86E-05	0.03735
s	Atopobacter phocae	0	2.03E-05	0.03735
s	Okeania sp. SIO3I5	0	1.47E-05	0.03735
s	Chrysiogenes arsenatis	0	1.11E-05	0.03735
s	Pseudoclavibacter caeni	0	1.05E-05	0.03735
s	Curtobacterium sp. 'Ferrero'	0	9.49E-06	0.03735
s	Candidatus Daviesbacteria bacterium GW2(	0	8.57E-06	0.03735
s	Trueperella bialowiezensis	0	8.41E-06	0.03735
s	Devosia sp. 67-54	0	8.30E-06	0.03735
s	Leclercia sp. J807	0	7.53E-06	0.03735
s	Ideonella sp. TBM-1	0	5.16E-06	0.03735

Table S3. Different species identified between I-PJS and NI-PJS

species	I-PJS-Mean	NI-PJS-Mean	P value
s Faecalibacterium prausnitzii	3.79	6.125	0.01188
s unclassified g Faecalibacter	1.111	1.958	0.007447
s Megamonas funiformis	0.2457	0.3084	0.03769
s Faecalibacterium sp.	0.244	0.3878	0.0171
s Collinsella sp. AF08-23	0.102	0.0003588	0.00771
s Romboutsia timonensis	0.07434	0.2162	0.04493
s Faecalibacterium sp. CAG:7	0.06698	0.07799	0.01779
s Faecalibacterium sp. AF10-4	0.04168	0.075	0.03905
s Enterobacter hormaechei	0.0355	0.02303	0.02924
s Fusobacterium sp.	0.03465	0.007255	0.02166
s Collinsella intestinalis	0.03286	0.001214	0.03637
s Enterobacter cloacae	0.02803	0.01304	0.04814
s Acinetobacter baumannii	0.02695	0.1329	0.03905
s Ruminococcus sp. OM08-7	0.0252	0.009796	0.03033
s Aeromonas veronii	0.0226	0.001125	0.008105
s Faecalibacterium sp. CAG:8	0.01995	0.0304	0.0171
s Ruminococcus sp. AM28-41	0.01986	0.006683	0.03509
s Dialister invisus CAG:218	0.01978	0.0008972	0.01529
s Alistipes senegalensis	0.01942	0.06684	0.02518
s Blautia sp. OF03-15BH	0.01876	0.03651	0.002477
s unclassified g Aeromonas	0.01843	0.01344	0.0114
s Lachnospiraceae bacterium (	0.01843	0.01294	0.03905
s Coprococcus sp. HPP0074	0.01744	0.009547	0.002039
s Coprococcus sp. AM27-12L	0.01593	0.009655	0.01644
s Proteobacteria bacterium CA	0.01509	0.003499	0.04182
s Ruminococcus sp. AF42-9B	0.01492	0.006594	0.02518
s Prevotella stercorea CAG:62	0.01457	0.06961	0.04701
s Fusobacterium necrogenes	0.01391	5.18E-05	0.0333
s Coprobacter fastidiosus	0.01388	0.04722	0.04814
s Bacteroides sp. AF14-46	0.01313	0.01612	0.02175
s Erwinia sp. OLMDSP33	0.01301	0.005204	0.04057
s Coprococcus sp. AF27-8	0.01237	0.005418	0.00106
s Enterobacter ludwigii	0.01199	0.007258	0.02924
s Ruminococcus sp. TM10-9A	0.01153	0.003309	0.01093
s Blautia caecimuris	0.01086	0.004155	0.03264
s Ruminococcus sp. AM29-26	0.01075	0.003763	0.02924
s [Clostridium] cellulosi	0.01024	0.0156	0.01644
s Blautia sp. CAG:237	0.01023	0.01538	0.01517
s Ruminococcus sp. AF24-32I	0.009736	0.004102	0.005225
s Ruminococcus sp. AM30-15	0.009413	0.00288	0.007778
s Haemophilus parainfluenzae	0.009292	0.03224	0.008121
s Coprococcus sp. HPP0048	0.006925	0.003481	0.04191
s Aureimonas altamirensis	0.00657	0.01054	0.01515
s Victivallales bacterium CCU	0.006292	0.01153	0.02717
s unclassified g Haemophilus	0.00567	0.01844	0.01162
s Clostridium sp. OM05-5BH	0.004706	0.00277	0.04651
s Coprococcus sp. TF11-13	0.004657	0.008877	0.04814
s Megamonas funiformis CAC	0.004608	0.00534	0.03708
s Cetobacterium sp. ZOR0034	0.004219	5.60E-05	0.01297
s Tannerella sp. AF04-6	0.004122	0.01683	0.02211
s Alistipes megaguti	0.003533	0.006477	0.02817
s Fusobacterium nucleatum	0.003453	0.0007157	0.02511
s Fusobacterium sp. CM21	0.003345	0.005522	0.01289
s Kluyvera georgiana	0.003273	0.0005042	0.01005
s Ruminococcus sp. AF31-16I	0.002979	0.001317	0.04046
s Aeromonas sp. 8C	0.002521	0.001213	0.04261
s Prevotella bryantii	0.002509	0.003877	0.04546

s	Enterococcus saigonensis	0.002477	0.003089	0.02817
s	Eubacterium sp. AM46-8	0.002335	0.003712	0.04814
s	Absiella sp. AM27-20	0.002147	0.001181	0.014
s	Coprococcus sp. CAG:131 4	0.002077	0.006856	0.02334
s	Bacteroides sp. CAG:714	0.002026	0.005074	0.02421
s	unclassified g Cetobacteriu	0.001955	4.32E-05	0.02947
s	Butyricimonas sp. Marseille-	0.001801	0.005196	0.001301
s	Bacteroides togonis	0.001495	0.008147	0.03264
s	Bacteroides sp. AF16-7	0.001474	0.002444	0.007681
s	Coprobacillus sp. 8 2 54BF	0.001471	0.002084	0.01579
s	Lachnoclostridium sp. An18	0.001465	0.0009394	0.03384
s	uncultured bacterium EB1	0.001438	0.002602	0.007603
s	Clostridium sp. AF35-15	0.001397	0.0007769	0.02334
s	Aeromonas hydrophila	0.001241	0.005203	0.04477
s	Fusobacterium sp. OBRC1	0.001145	9.04E-06	0.0139
s	Bacteroides sp. OM05-10AA	0.001041	0.0002054	0.04932
s	Clostridium sp. CAG:632	0.001011	0.001785	0.02243
s	Clostridiales bacterium VE2	0.000997	0.002157	0.01517
s	Romboutsia sp. Marseille-P6	0.0009685	0.002277	0.03051
s	Candidatus Nitrosoglobus te	0.0008919	7.92E-05	0.0426
s	Turicibacter sp. H121	0.0008792	0.003363	0.01818
s	Prevotella multisaccharivorax	0.0007739	0.0009742	0.04946
s	Barnesiella sp. An22	0.0007568	0.001318	0.02424
s	bacterium ADurb.Bin431	0.0007541	0.0004043	0.02247
s	Bacteroides kribbi	0.0007474	0.001409	0.04479
s	Candidatus Gastranaerophila	0.0007129	3.13E-05	0.01709
s	Lachnospiraceae bacterium I	0.0007086	0.0001795	0.0267
s	uncultured Prevotella sp.	0.0006517	2.95E-06	0.01186
s	Aeromonas allosaccharophila	0.0006398	2.97E-05	0.04975
s	unclassified f Actinomyceta	0.0005573	0.0005644	0.02817
s	Flavobacterium sp.	0.0005308	8.52E-05	0.002004
s	Bifidobacterium thermophilu	0.0005291	0.001376	0.04903
s	Prevotella melaninogenica	0.0005285	0.00283	0.03345
s	Thiomonas sp. CB2	0.0005252	0.001059	0.03015
s	Clostridium sp. AF18-27	0.0005208	6.16E-05	0.005246
s	Haemophilus influenzae	0.0005057	0.001339	0.04945
s	Azospirillum sp. CAG:239	0.0004893	0.00379	0.02009
s	Dechloromonas sp. Dech201	0.000468	0.001009	0.01635
s	Succinatimonas hippei	0.0004339	3.82E-05	0.009725
s	Collinsella sp. An271	0.0004102	5.75E-05	0.02127
s	Bacteroides sp. AM10-21B	0.0003884	0	0.01709
s	Butyrivibrio sp. FCS014	0.0003778	0.0003894	0.01557
s	Pseudobutyrivibrio sp. 49	0.0003579	0.000201	0.02115
s	unclassified f Pasteurellace	0.0003483	0.0008931	0.01788
s	Prevotella sp. P5-50	0.0003461	0.0009196	0.01175
s	Enterobacter sichuanensis	0.0003359	0.0001754	0.02611
s	Lentisphaerae bacterium GW	0.0003229	2.62E-05	0.01709
s	unclassified g Thermoanae	0.0002936	6.18E-05	0.04731
s	Salmonella sp.	0.0002868	0.0001536	0.03397
s	Prevotella shahii	0.0002807	0.00131	0.01621
s	Enterobacter timonensis	0.0002798	0.0001135	0.0422
s	Heliophilum fasciatum	0.0002726	8.46E-06	0.0324
s	Enterobacter sp. 50588862	0.0002573	0.0001046	0.04617
s	Butyrivibrio sp. INlla14	0.0002529	0.0001194	0.04534
s	Acidobacteria bacterium RIF	0.0002523	0	0.04802
s	Nitrospirae bacterium GWF2	0.0002508	0	0.02873
s	Acetobacterium sp. KB-1	0.0002494	5.94E-05	0.02854
s	Bacteroides sp. AF39-11AC	0.0002492	2.27E-05	0.008039
s	Candidatus Magasanikbacter	0.000243	2.11E-05	0.04479

s	Clostridium chromiireducens	0.0002295	0.000634	0.01224
s	Paenibacillus thermoaerophil	0.0002295	6.79E-05	0.01685
s	Enterobacter sp. M4-VN	0.0002278	0.0001938	0.02502
s	Enterococcus columbae	0.0002259	0.0001796	0.03456
s	Vibrio vulnificus	0.0001986	6.19E-05	0.02048
s	Erysipelatoclostridium sp. A	0.0001941	0.0005157	0.04175
s	Niabella sp.	0.000193	9.79E-05	0.04026
s	Haemophilus sp. HMSC61B	0.0001888	0.0007068	0.01901
s	Haemophilus sp. HMSC068	0.0001798	0.000584	0.01979
s	Prevotella sp. S7 MS 2	0.0001679	0.0002472	0.04506
s	Enterobacteriaceae bacterium	0.000164	0.0002447	0.0277
s	Haemophilus sp. HMSC71H	0.0001632	0.00055	0.01343
s	uncultured bacterium Contig	0.0001616	0.0001913	0.0493
s	Butyrivibrio sp. YAB3001	0.0001605	0.0003067	0.03374
s	Anaerolineae bacterium	0.0001572	0.0002437	0.01517
s	Haemophilus sp. CCUG 60	0.0001537	0.0005119	0.01317
s	Bacteroides sp. AF16-49	0.0001523	9.03E-05	0.03465
s	Turicibacter sp. HGF1	0.0001478	0.000609	0.04214
s	Desulfosporosinus youngiae	0.000142	0.000175	0.03119
s	Enterobacter sp. N18-03635	0.0001407	6.52E-06	0.03717
s	Enterobacter sp. NFIX45	0.0001402	8.89E-05	0.01259
s	Clostridium putrefaciens	0.0001399	0.0001541	0.03554
s	Zymobacter palmae	0.0001386	0.0002908	0.006329
s	Tissierella creatinini	0.0001364	0.0001089	0.04381
s	Clostridium sp. Marseille-P4	0.0001355	0.0004353	0.04438
s	Paenibacillus wynnii	0.0001342	8.41E-05	0.04606
s	Prevotella sp. oral taxon 37	0.0001324	0.0005072	0.02226
s	Lactobacillus agilis	0.0001319	0.007542	0.0191
s	Rhizobium etli	0.0001317	8.43E-05	0.03385
s	Candidatus Gastranaerophila	0.0001292	0.0003255	0.01437
s	Capnocytophaga felis	0.0001282	0	0.04802
s	Bifidobacterium sp. N4G05	0.0001267	4.55E-05	0.03337
s	Corynebacterium durum	0.0001246	5.02E-05	0.00588
s	Enterobacter sp. ku-bf2	0.0001224	0	0.01709
s	Prevotella baroniae	0.0001217	0.0005067	0.04178
s	Clostridium sp. CAG:967	0.0001208	0.0002145	0.03328
s	Polaribacter sp. 20A6	0.0001204	2.77E-05	0.04361
s	Selenomonas sp. oral taxon	0.0001198	0.0004976	0.04802
s	Jonquetella anthropi	0.0001177	2.02E-05	0.04084
s	Aerococcus urinae	0.0001163	1.40E-05	0.04946
s	Bifidobacterium minimum	0.0001148	0.0003957	0.001039
s	Tepidibacillus fermentans	0.0001108	0.0001063	0.01286
s	Clostridium nigeriense	0.0001065	0.0002669	0.03547
s	Cohnella sp. CC-MHH1044	0.0001053	0.000181	0.04041
s	Alkaliphilus transvaalensis	0.000105	6.05E-06	0.0262
s	Atopobium sp. oral taxon 1	0.0001036	1.80E-06	0.008365
s	Bacillus toyonensis	9.87E-05	0.0007082	0.01088
s	Agrobacterium vitis	9.74E-05	0	0.04802
s	Selenomonas sp. oral taxon	9.67E-05	1.09E-05	0.00848
s	Cronobacter condimenti	9.42E-05	3.98E-05	0.02009
s	Clostridium tepidiprofundi	9.27E-05	2.24E-05	0.02537
s	Pseudomonas xanthomarina	8.99E-05	0.0001549	0.03873
s	Prevotella sp. oral taxon 31	8.96E-05	0.001114	0.02878
s	Legionella quinlivanii	8.93E-05	0.0003352	0.02377
s	Arcobacter lekithochrous	8.68E-05	0	0.02873
s	Chloroflexi bacterium ADur	7.91E-05	5.50E-05	0.02472
s	Bacillus sp. P16(2019)	7.91E-05	0	0.04802
s	Treponema socranskii	7.61E-05	0.0002666	0.00773
s	Acinetobacter sp. CAG:196	7.56E-05	0.0005041	0.04812

s	Butyrivibrio sp. Su6	7.45E-05	0.0001988	0.01965
s	Wenyingzhuangia heitensis	6.89E-05	0	0.04802
s	Haemophilus sputorum	6.83E-05	0.0002156	0.01955
s	Ochrobactrum anthropi	6.76E-05	2.62E-06	0.0116
s	Sphingobacteriales bacterium	6.75E-05	0	0.01709
s	Pelosinus sp. UFO1	6.36E-05	1.21E-05	0.02575
s	Paenibacillus sp. IHBB 103	6.31E-05	0	0.04802
s	Hapalosiphonaceae cyanobac	6.22E-05	0	0.04802
s	[Enterobacter] lignolyticus	6.08E-05	5.78E-06	0.02201
s	Bacillus amyloliquefaciens	5.94E-05	0	0.01709
s	Dysgonomonas mossii	5.79E-05	0.0003299	0.02906
s	Butyrivibrio sp. NC2007	5.71E-05	0.0001454	0.001682
s	Rheinheimera sp.	5.33E-05	0	0.04802
s	Gardnerella sp. KA00735	5.30E-05	0	0.02873
s	Acetobacterium sp. MES1	5.26E-05	0.0001471	0.04876
s	unclassified g Acutalibacte	5.25E-05	0	0.02873
s	Bacillus massiliogorillae	5.23E-05	1.93E-05	0.03077
s	Paenibacillus chibensis	5.19E-05	2.20E-05	0.03395
s	Parabacteroides sp. P14	5.11E-05	2.35E-06	0.03849
s	uncultured spirochete	5.03E-05	7.06E-05	0.01434
s	Bacteroidetes bacterium 46-	4.89E-05	0	0.04802
s	Moraxella pluranimalium	4.88E-05	2.71E-07	0.01922
s	Dysgonomonas sp. 37-18	4.69E-05	6.30E-05	0.03091
s	Candidatus Aminicenantes b	4.27E-05	3.18E-06	0.01482
s	Pseudodesulfovibrio sp. zrk4	4.02E-05	1.69E-05	0.03891
s	Mucilaginibacter yixingensis	3.98E-05	0.0001095	0.03249
s	Citrobacter sp. TBCS-15	3.86E-05	4.04E-06	0.0405
s	Micavibrio aeruginosavorus	3.74E-05	7.83E-05	0.03146
s	Denitrobacterium detoxifican	3.64E-05	0.000146	0.03426
s	Acinetobacter haemolyticus	3.64E-05	6.45E-06	0.04205
s	Granulicatella sp. zg-ZJ	3.64E-05	0	0.04802
s	Bacteroidetes bacterium HG	3.52E-05	0	0.04802
s	Aggregatibacter sp. oral tax	3.42E-05	9.02E-05	0.01114
s	Tychonema bourrellyi	3.37E-05	0	0.01008
s	Enterobacter sp. MGH 6	3.30E-05	1.34E-05	0.01705
s	Lactobacillus gorillae	3.30E-05	0	0.02873
s	Haemophilus pittmaniae	3.29E-05	0.0001706	0.04992
s	Enterobacter sp. BIDMC110	3.27E-05	1.00E-05	0.04478
s	Flavobacteria bacterium RIF	3.25E-05	0	0.04802
s	Enterobacter sp. MGH 3	3.15E-05	0	0.04802
s	Ewingella americana	3.03E-05	1.70E-06	0.01482
s	Enterobacteriaceae bacterium	2.97E-05	0	0.04802
s	Firmicutes bacterium HGW-	2.97E-05	7.33E-05	0.03214
s	Paenibacillus durus	2.95E-05	5.54E-05	0.04359
s	Campylobacter geochelonis	2.93E-05	7.90E-06	0.04708
s	Aggregatibacter segnis	2.87E-05	0.000148	0.02918
s	unclassified g Kluyvera	2.83E-05	5.10E-06	0.03168
s	Salipiger marinus	2.79E-05	0	0.04802
s	Moraxella sp.	2.78E-05	0.0001008	0.03322
s	Lagierella massiliensis	2.72E-05	8.58E-05	0.01755
s	Actinomyces sp. HMSC075	2.68E-05	3.88E-05	0.01724
s	Aeromonas sp. 9A	2.59E-05	0.00016	0.01709
s	Haemophilus sp. HMSC073	2.57E-05	6.17E-05	0.028
s	Clostridium autoethanogenum	2.51E-05	5.24E-05	0.03941
s	Paenibacillus sp. DCT19	2.47E-05	3.62E-06	0.04886
s	Parapedobacter sp.	2.46E-05	0	0.04802
s	Flavobacterium faecale	2.36E-05	4.87E-06	0.04706
s	Aminobacterium colombiens	2.31E-05	0	0.04802
s	Bacteroidetes bacterium GW	2.27E-05	0	0.04802

s	Tumebacillus avium	2.21E-05	0.0001544	0.0246
s	Bifidobacterium tissieri	2.20E-05	4.69E-05	0.04191
s	Clostridium tetanomorphum	2.19E-05	4.91E-05	0.01148
s	Macrococcus goetzii	2.15E-05	3.56E-06	0.0426
s	bacterium 1XD42-94	2.15E-05	0.0001924	0.02592
s	Franconibacter helveticus	2.14E-05	0	0.04802
s	unclassified g Calothrix	2.06E-05	2.76E-05	0.02472
s	Candidatus Thermofonsia Cl	2.06E-05	7.62E-06	0.0234
s	Fibrobacter sp. UWB7	2.02E-05	0.0003357	0.02112
s	Kibdelosporangium sp. MJ1	2.00E-05	0	0.04802
s	Bifidobacterium magnum	1.90E-05	0.0002206	0.04946
s	Dickeya zeae	1.83E-05	0	0.04802
s	Lactobacillus pasteurii	1.78E-05	0	0.02873
s	Facklamia hominis	1.76E-05	0.0001146	0.01175
s	Fibrobacter sp. UWCM	1.74E-05	0.0005818	0.04537
s	Streptomyces alboflavus	1.71E-05	1.57E-06	0.03972
s	Brevibacillus sp. CFH S050	1.64E-05	0	0.04802
s	Sphingobacteriales bacterium	1.64E-05	0.0001137	0.0447
s	unclassified g Thermoanae	1.63E-05	0	0.04802
s	Clostridium sp. CT7	1.63E-05	1.98E-06	0.03972
s	Paludibacter propionicigenes	1.58E-05	0.000273	0.04449
s	Firmicutes bacterium ZCTH	1.50E-05	0	0.02873
s	Sphingobacterium lactis	1.49E-05	0	0.02873
s	Parendozoicomonas haliclona	1.45E-05	0	0.01008
s	Trueperella bialowiezensis	1.42E-05	6.30E-07	0.03538
s	[Mannheimia] succiniciprodu	1.39E-05	5.97E-05	0.02439
s	alpha proteobacterium BAL	1.33E-05	2.41E-06	0.007075
s	Gelria sp.	1.31E-05	2.82E-06	0.04706
s	bacterium 1xD8-27	1.28E-05	0	0.04802
s	Nitrosomonas sp. Nm33	1.27E-05	0	0.01709
s	Bacillus paralicheniformis	1.26E-05	0	0.02873
s	Photobacterium galatheae	1.25E-05	0	0.04802
s	Hymenobacter nivis	1.23E-05	0	0.02873
s	Bacillus infantis	1.13E-05	0	0.003396
s	Acidovorax sp. MR-S7	1.09E-05	0	0.04802
s	Bacillus sp. V3-13	1.08E-05	1.88E-05	0.0246
s	unclassified g Massilimalia	1.08E-05	2.58E-06	0.03545
s	Chromobacterium sp. IIBBL	1.05E-05	0	0.04802
s	Pseudozobellia thermophila	1.04E-05	2.83E-05	0.04075
s	Chitinophaga skermanii	1.03E-05	0	0.04802
s	Actinobacillus equuli	1.01E-05	2.72E-05	0.02593
s	Thalassolituus sp. CG17 big	9.93E-06	0	0.04802
s	Paenibacillus sp. HB172198	9.68E-06	2.88E-05	0.02715
s	unclassified g Mediterranea	9.51E-06	4.57E-05	0.03806
s	Cohnella thermotolerans	9.48E-06	2.81E-05	0.0065
s	Corynebacterium sp. HMSC	9.40E-06	0.0001276	0.02508
s	Streptococcus sp. DD10	9.38E-06	4.70E-05	0.03465
s	Cyanothece sp. SIO2G6	9.30E-06	1.22E-06	0.03849
s	Staphylococcus caprae	9.26E-06	0	0.01709
s	Tardiphaga sp. YR296	9.22E-06	0	0.04802
s	Paenibacillus sp. RU4T	9.12E-06	0	0.02873
s	Lactobacillus intestinalis	9.10E-06	1.28E-05	0.03979
s	Clostridiales bacterium 38-1	9.00E-06	6.41E-05	0.007649
s	Varibaculum vaginae	8.99E-06	0.0007539	0.01709
s	Tenericutes bacterium HGW	8.98E-06	0	0.04802
s	Prevotella brunnea	8.89E-06	0.0001382	0.004499
s	Curvibacter sp. PD MW3	8.45E-06	5.27E-05	0.02184
s	Arcanobacterium phocae	8.29E-06	0	0.04802
s	Oceanobacillus iheyensis	8.25E-06	0.0001292	0.006121

s	Clostridium sp. FP1	8.14E-06	4.29E-05	0.01278
s	Jiangella alba	8.11E-06	8.63E-06	0.04823
s	Paenibacillus sp. LK1	8.03E-06	0	0.04802
s	Desulfosarcina cetonica	7.88E-06	0	0.02873
s	Anabaena sp. UHCC 0204	7.49E-06	0	0.04802
s	Peptococcaceae bacterium B	7.44E-06	7.11E-05	0.007531
s	Psychromonas ossibalaenae	7.29E-06	0	0.04802
s	Bacillus sp. AFS006103	6.90E-06	5.97E-05	0.01319
s	Actinomyces howellii	6.34E-06	3.34E-05	0.01523
s	Prevotella nanceiensis	6.28E-06	8.46E-05	0.02184
s	Melghirimyces algeriensis	6.26E-06	3.67E-05	0.01918
s	Candidatus Caldatribacteriun	6.18E-06	0	0.04802
s	Melioribacter sp.	5.85E-06	0	0.04802
s	Pseudovibrio sp. Ad37	5.67E-06	1.10E-05	0.03146
s	Kamptonema sp. PCC 6506	5.64E-06	1.18E-05	0.04361
s	Bacillus sp. 95MFCvi2.1	5.50E-06	5.37E-06	0.04361
s	Burkholderia gladioli	5.44E-06	5.46E-05	0.02324
s	Flavobacterium columnare	5.38E-06	0.0003078	0.007862
s	Prevotella sp. HJM029	5.15E-06	6.40E-05	0.006991
s	Bacillus shackletonii	4.88E-06	4.31E-05	0.03551
s	Microlunatus soli	4.64E-06	0	0.04802
s	unclassified c Betaproteoba	3.99E-06	2.72E-05	0.04537
s	Vibrio anguillarum	3.87E-06	3.34E-05	0.01093
s	Gloeocapsa sp. PCC 7428	3.72E-06	6.14E-06	0.03146
s	Haemophilus sp. CCUG 66	3.69E-06	3.50E-05	0.0405
s	Prevotella sp. Marseille-P43	3.42E-06	0.0002174	0.007384
s	Ignavibacteriae bacterium	3.31E-06	4.52E-05	0.006014
s	Paraburkholderia kirstenbosc	3.28E-06	1.30E-05	0.02933
s	Scytonema sp. HK-05	3.15E-06	1.32E-05	0.04946
s	Paenibacillus sp. YH-JAE5	3.08E-06	2.62E-05	0.04046
s	Bifidobacterium tibiigranuli	2.99E-06	0.0001202	0.04537
s	Paludifilum halophilum	2.71E-06	0.0001099	0.01479
s	Rothia sp. HMSC067H10	2.26E-06	1.51E-05	0.04027
s	Bacillus sp. EB01	2.24E-06	3.00E-05	0.04946
s	Poseidonocella sp. HB16139	2.16E-06	9.36E-06	0.04291
s	Pediococcus damnosus	1.94E-06	1.84E-05	0.04537
s	Lactobacillus equigenerosi	1.92E-06	4.50E-05	0.03311
s	Geobacillus sp. 12AMOR1	1.87E-06	6.85E-05	0.03311
s	Actinotignum urinale	1.87E-06	4.62E-05	0.01375
s	Microbacterium endophyticu	1.43E-06	1.19E-05	0.01709
s	Bordetella hinzii	1.10E-06	1.52E-05	0.03551
s	Aquisphaera giovannonii	1.07E-06	9.97E-06	0.009595
s	Streptomyces thermoautotrop	1.07E-06	9.11E-06	0.04276
s	Kurthia huakuii	9.14E-07	1.24E-05	0.006014
s	Porphyromonadaceae bacteri	8.70E-07	6.91E-05	0.008983
s	Melissococcus sp. OM08-11	8.25E-07	1.19E-05	0.01479
s	Pedobacter sp. BS3	7.90E-07	4.58E-05	0.005162
s	Chloroflexi bacterium ADur	5.08E-07	5.15E-06	0.03443
s	Actinomyces nasicola	3.56E-07	2.75E-05	0.01277
s	Fibrobacter sp. UWH5	2.43E-07	1.61E-05	0.006014
s	Desmospora activa	2.17E-07	1.87E-05	0.005574
s	Mycoplasma alvi	0	2.32E-05	0.001306
s	Cyanothece sp. SIO1E1	0	4.65E-05	0.003187
s	Hydrogenibacillus schlegelii	0	2.10E-05	0.003187
s	Lactobacillus crustorum	0	8.14E-05	0.007654
s	Euzebyella marina	0	2.30E-05	0.007654
s	Azovibrio restrictus	0	1.74E-05	0.007654
s	Actinobacteria bacterium CC	0	7.50E-06	0.007654
s	Lactobacillus sp. 143-6	0	0.0002028	0.01817

s	Algibacter sp. L3A6	0	0.0001648	0.01817
s	Listeria grayi	0	4.32E-05	0.01817
s	Mucilaginibacter sp. JXJ CY	0	3.91E-05	0.01817
s	Pantoea rwandensis	0	3.50E-05	0.01817
s	Uliginosibacterium gangwon	0	3.01E-05	0.01817
s	uncultured alpha proteobacte	0	2.86E-05	0.01817
s	Achromobacter pulmonis	0	2.82E-05	0.01817
s	Alphaproteobacteria bacteriu	0	2.73E-05	0.01817
s	Enterococcus sp. HMSC067	0	2.50E-05	0.01817
s	Sporomusaceae bacterium	0	2.09E-05	0.01817
s	Lactobacillus alimentarius	0	1.89E-05	0.01817
s	Nonlabens sp. YIK11	0	1.88E-05	0.01817
s	Rhodospirillaceae bacterium	0	1.86E-05	0.01817
s	Peribacillus kribbensis	0	1.80E-05	0.01817
s	Sphingobacterium sp. CZ-U	0	1.50E-05	0.01817
s	Bacillus sp. CH30 1T	0	1.48E-05	0.01817
s	Pseudorhodobacter psychrote	0	1.39E-05	0.01817
s	Petrotoga sp. 9PW.55.5.1	0	1.28E-05	0.01817
s	Psychroserpens sp. Hel I 66	0	1.09E-05	0.01817
s	Xanthomonas phaseoli	0	9.65E-06	0.01817
s	unclassified g Aeromicrobi	0	8.39E-06	0.01817
s	Leeuwenhoekiella sp. MAR	0	7.83E-06	0.01817
s	Marmoricola caldifontis	0	7.74E-06	0.01817
s	Segetibacter koreensis	0	7.08E-06	0.01817
s	Rhodospirillaceae bacterium	0	6.85E-06	0.01817
s	Flaviflexus massiliensis	0	3.58E-06	0.01817
s	Rummeliibacillus sp. TYF00	0	1.91E-06	0.01817
s	Myroides guanonis	0	0.0003967	0.043
s	Leclercia sp. 119287	0	0.0002256	0.043
s	Citrobacter sp. wls613	0	0.0002205	0.043
s	unclassified g Apibacter	0	0.0001791	0.043
s	Lysinibacillus sp. AR18-8	0	0.0001787	0.043
s	Flavobacterium sp. LB1R34	0	0.000139	0.043
s	Flavobacterium frigidimaris	0	0.0001039	0.043
s	Actinobacteria bacterium HO	0	8.24E-05	0.043
s	Parcubacteria group bacteriu	0	7.71E-05	0.043
s	Thermodesulfovibrio yellows	0	7.29E-05	0.043
s	Zetaproteobacteria bacterium	0	7.22E-05	0.043
s	uncultured bacterium 24i11	0	7.22E-05	0.043
s	Pseudomonas borbori	0	7.18E-05	0.043
s	Gemmataceae bacterium	0	7.04E-05	0.043
s	Flavobacteriaceae bacterium	0	6.61E-05	0.043
s	bacterium (Candidatus Black	0	5.71E-05	0.043
s	Candidatus Lloydbacteria ba	0	5.17E-05	0.043
s	Cytophagaceae bacterium 50	0	4.79E-05	0.043
s	Erwinia persicina	0	3.10E-05	0.043
s	Enterococcus sp. HMSC061	0	3.08E-05	0.043
s	Cellulophaga sp. L1A9	0	2.96E-05	0.043
s	Enterococcus aquimarinus	0	2.93E-05	0.043
s	Chlorobium phaeobacteroide	0	2.81E-05	0.043
s	Candidatus Halocyntiibacter	0	2.74E-05	0.043
s	Candidatus Aminicenantes b	0	2.72E-05	0.043
s	Bacillus sp. OV166	0	2.69E-05	0.043
s	Ferrovum sp. Z-31	0	2.69E-05	0.043
s	Lactobacillus sp. 54-5	0	2.61E-05	0.043
s	Bacillus sp. BB51/4	0	2.61E-05	0.043
s	Paenibacillus sp. 276b	0	2.60E-05	0.043
s	Flammeovirga yaeyamensis	0	2.26E-05	0.043
s	Streptococcus castoreus	0	2.20E-05	0.043

s	unclassified f Flavobacteria	0	2.10E-05	0.043
s	Nitrospinae bacterium RIFC	0	2.09E-05	0.043
s	Chryseobacterium sp. AG36	0	1.72E-05	0.043
s	Dehalobacter sp. CF	0	1.69E-05	0.043
s	Kurthia gibsonii	0	1.45E-05	0.043
s	Cyanobacteria bacterium UE	0	1.45E-05	0.043
s	Alkalihalobacillus hwajinpoe	0	1.43E-05	0.043
s	Flavobacteriaceae bacterium	0	1.34E-05	0.043
s	Enterococcus sp. T0168A.B	0	1.28E-05	0.043
s	Mycolicibacterium brumae	0	1.21E-05	0.043
s	Nitrosomonadales bacterium	0	1.19E-05	0.043
s	Siphonobacter curvatus	0	1.15E-05	0.043
s	Cytobacillus solani	0	1.10E-05	0.043
s	Bacillus sp. G3(2015)	0	1.09E-05	0.043
s	Ornithinicoccus hortensis	0	1.05E-05	0.043
s	Globicatella sp. HMSC072A	0	1.04E-05	0.043
s	Exiguobacterium sp. SL-9	0	9.93E-06	0.043
s	Corynebacterium ureicelerivo	0	9.82E-06	0.043
s	Sanguibacter sp. Leaf3	0	9.35E-06	0.043
s	Francisella noatunensis	0	8.92E-06	0.043
s	Candidatus Arthromitus sp.	0	8.38E-06	0.043
s	Kangiella koreensis	0	8.37E-06	0.043
s	Vibrio hepatarius	0	8.31E-06	0.043
s	Bacillus galactosidilyticus	0	8.30E-06	0.043
s	Cloacibacterium normanense	0	8.02E-06	0.043
s	Staphylococcus chromogenes	0	7.94E-06	0.043
s	Schaalia suimastitidis	0	7.82E-06	0.043
s	Sinomicrobium pectinilyticu	0	7.82E-06	0.043
s	Agrobacterium bohemicum	0	7.32E-06	0.043
s	Embleya scabrispora	0	7.13E-06	0.043
s	Candidatus Thiodictyon syn	0	7.06E-06	0.043
s	Peptoniphilus ivorii	0	6.60E-06	0.043
s	Streptomyces eurocidicus	0	6.58E-06	0.043
s	Microbacterium sp. 3J1	0	6.57E-06	0.043
s	Robertkochia solimangrovi	0	6.11E-06	0.043
s	Rhodohalobacter sp. SW132	0	6.08E-06	0.043
s	Armatimonadetes bacterium	0	6.02E-06	0.043
s	unclassified g Nocardioides	0	5.75E-06	0.043
s	Aliifodinibius roseus	0	4.91E-06	0.043
s	Bacillus sp. KH172YL63	0	3.78E-06	0.043
s	Chryseobacterium sp. JAH	0	3.76E-06	0.043
s	Aerococcus sp. HMSC061A	0	3.21E-06	0.043
s	Psychrobacillus sp. AK 181	0	3.08E-06	0.043
s	unclassified g Christensene	0	3.05E-06	0.043
s	Staphylococcus devriesei	0	2.96E-06	0.043
s	Ursidibacter arcticus	0	2.88E-06	0.043
s	Komagataeibacter sacchariv	0	2.20E-06	0.043
s	Alkalihalobacillus patagonie	0	1.90E-06	0.043

Table S4. Different KEGG pathways identified between PJS patients and healthy family controls

pathways (level 3)	Control-Mean	PJS-Mean	P value
Biosynthesis of secondary metabolites	8.746	8.536	3.84E-05
Microbial metabolism in diverse environ	4.427	4.614	0.005949
Biosynthesis of amino acids	4.069	3.832	7.69E-05
Two-component system	1.795	2	0.001237
Starch and sucrose metabolism	1.521	1.435	0.02543
Amino sugar and nucleotide sugar metab	1.478	1.414	0.02104
Glycolysis / Gluconeogenesis	1.17	1.138	0.003898
Aminoacyl-tRNA biosynthesis	1.074	1.013	0.04177
Alanine, aspartate and glutamate metabol	1.049	1.002	0.0001038
Peptidoglycan biosynthesis	1.018	0.9754	0.03502
Pyruvate metabolism	0.9654	1.008	0.004248
Pentose phosphate pathway	0.8284	0.7931	0.003473
2-Oxocarboxylic acid metabolism	0.8259	0.7815	0.003575
Phenylalanine, tyrosine and tryptophan b	0.7069	0.6692	0.001022
Glyoxylate and dicarboxylate metabolism	0.6715	0.7032	0.02426
Lysine biosynthesis	0.6227	0.5838	0.000512
RNA degradation	0.6207	0.5899	0.0009583
Butanoate metabolism	0.5754	0.6111	0.002511
Propanoate metabolism	0.55	0.5867	0.02426
Thiamine metabolism	0.5484	0.5291	0.03129
Fatty acid metabolism	0.5434	0.5177	0.005034
Cell cycle - Caulobacter	0.5298	0.5038	0.03129
Bacterial secretion system	0.527	0.5798	0.00309
Arginine biosynthesis	0.5266	0.5	0.001494
Fatty acid biosynthesis	0.5212	0.4745	0.0001344
Pantothenate and CoA biosynthesis	0.512	0.501	0.0237
Nucleotide excision repair	0.4881	0.4604	0.02484
Protein export	0.4815	0.4666	0.04653
Glycerophospholipid metabolism	0.4552	0.4862	0.005034
Cyanoamino acid metabolism	0.4197	0.3789	0.01733
Valine, leucine and isoleucine biosynthes	0.4126	0.3968	0.04554
Arginine and proline metabolism	0.3979	0.416	0.0182
Histidine metabolism	0.3925	0.3537	1.00E-04
Cationic antimicrobial peptide (CAMP) r	0.3557	0.4104	0.005177
Streptomycin biosynthesis	0.3546	0.3264	0.00368
Photosynthesis	0.3429	0.3063	0.002295
Vancomycin resistance	0.3423	0.3224	0.01421
Sulfur metabolism	0.3404	0.3676	0.04362
Biofilm formation - Escherichia coli	0.3075	0.3822	0.00159
Lipopolysaccharide biosynthesis	0.258	0.3295	0.002913
Biofilm formation - Vibrio cholerae	0.2403	0.2933	0.0006914
Glucagon signaling pathway	0.2335	0.2143	0.001277
Necroptosis	0.2139	0.1996	0.002511
HIF-1 signaling pathway	0.1895	0.1839	0.04554
Phenylalanine metabolism	0.1834	0.2251	0.004894
Fatty acid degradation	0.1786	0.1988	0.001237
Polyketide sugar unit biosynthesis	0.177	0.1632	0.01018
Valine, leucine and isoleucine degradatio	0.1755	0.1878	0.01045
Glutathione metabolism	0.1698	0.2209	0.0004036
RNA polymerase	0.1687	0.1581	0.04458
Tuberculosis	0.1514	0.1417	0.01911
PPAR signaling pathway	0.1489	0.1393	0.03581
Insulin resistance	0.1448	0.1336	0.006461
Tyrosine metabolism	0.1418	0.1604	0.007812
Prodigiosin biosynthesis	0.1379	0.129	0.01284
Lysine degradation	0.1366	0.1818	9.63E-05
Ascorbate and aldarate metabolism	0.1296	0.1583	0.005949

Ubiquinone and other terpenoid-quinone	0.1267	0.1765	0.0001249
Biofilm formation - Pseudomonas aerugi	0.1225	0.1482	0.01691
Novobiocin biosynthesis	0.1219	0.1162	0.01911
Benzoate degradation	0.1205	0.1542	0.0004321
Ferroptosis	0.1082	0.09781	0.04269
Insulin signaling pathway	0.1078	0.1008	0.032
Adipocytokine signaling pathway	0.09854	0.08776	0.02727
Thermogenesis	0.09688	0.08887	0.04856
Phosphonate and phosphinate metabolism	0.0889	0.1026	0.008024
Carbapenem biosynthesis	0.08806	0.08243	0.002746
Tryptophan metabolism	0.08744	0.1092	0.000231
RNA transport	0.08662	0.08215	0.03058
Degradation of aromatic compounds	0.08274	0.1343	5.89E-05
Glucosinolate biosynthesis	0.08177	0.07496	0.001404
beta-Alanine metabolism	0.07892	0.1059	0.0001077
Type II diabetes mellitus	0.05313	0.0475	0.0001798
Pertussis	0.05296	0.0845	1.80E-05
Penicillin and cephalosporin biosynthesis	0.05133	0.04107	0.00664
Human papillomavirus infection	0.05016	0.04421	0.0001077
Viral carcinogenesis	0.05008	0.04413	9.63E-05
Pathways in cancer	0.04982	0.0593	0.0001932
Isoquinoline alkaloid biosynthesis	0.04635	0.05072	0.02664
Secondary bile acid biosynthesis	0.0441	0.03753	0.00159
Primary bile acid biosynthesis	0.04168	0.03523	0.002227
Lipoic acid metabolism	0.03278	0.03711	0.04269
Aminobenzoate degradation	0.03181	0.04165	0.003575
Carbohydrate digestion and absorption	0.03032	0.03777	0.02315
Nitrotoluene degradation	0.02981	0.04252	0.01532
Pathogenic Escherichia coli infection	0.02977	0.03406	0.01101
Platinum drug resistance	0.02801	0.03675	0.003182
Biosynthesis of siderophore group nonrib	0.0278	0.05296	0.0003281
Pancreatic secretion	0.02727	0.0344	0.0279
FoxO signaling pathway	0.02068	0.02576	0.0226
Huntington disease	0.01994	0.02714	0.001055
Proximal tubule bicarbonate reclamation	0.0185	0.02261	0.0161
Synthesis and degradation of ketone bodi	0.01813	0.02423	0.00309
Longevity regulating pathway	0.01794	0.02326	0.008242
Autophagy - yeast	0.01787	0.01347	0.005177
Limonene and pinene degradation	0.01703	0.02473	9.28E-05
Chlorocyclohexane and chlorobenzene de	0.0161	0.0228	0.000495
Xylene degradation	0.01284	0.0242	0.0001932
Metabolism of xenobiotics by cytochrom	0.01275	0.02399	0.0001446
MAPK signaling pathway - fly	0.01249	0.01535	0.02855
Amyotrophic lateral sclerosis (ALS)	0.01238	0.01916	5.25E-05
Drug metabolism - cytochrome P450	0.01234	0.02392	0.0001446
Dioxin degradation	0.01157	0.02047	0.001022
Retinol metabolism	0.01118	0.01528	0.002913
Arabinogalactan biosynthesis - Mycobact	0.01096	0.01304	0.03348
Caprolactam degradation	0.009035	0.01758	0.0006471
Thyroid hormone synthesis	0.007266	0.0158	2.03E-05
Bacterial invasion of epithelial cells	0.006927	0.01743	0.02315
Geraniol degradation	0.006766	0.02118	3.69E-05
Arachidonic acid metabolism	0.006675	0.01188	9.28E-05
MAPK signaling pathway - yeast	0.005445	0.007853	0.006824
Cushing syndrome	0.004489	0.006621	0.002587
Renal cell carcinoma	0.004486	0.006583	0.002746
Biosynthesis of unsaturated fatty acids	0.004418	0.01035	0.0003397
Ether lipid metabolism	0.004387	0.006925	0.002746
African trypanosomiasis	0.004293	0.006428	0.0182

Chagas disease (American trypanosomias	0.00417	0.006014	0.02855
Hepatocellular carcinoma	0.004088	0.01279	1.87E-05
Chemical carcinogenesis	0.003698	0.01484	2.39E-05
alpha-Linolenic acid metabolism	0.003676	0.01146	2.69E-05
Fluorobenzoate degradation	0.003103	0.01299	1.95E-05
Toluene degradation	0.002891	0.01186	3.41E-05
Yersinia infection	0.002358	0.01185	0.005177
Ethylbenzene degradation	0.002148	0.007305	5.25E-05
Bladder cancer	0.001817	0.0049	0.0001118
Prion diseases	0.001795	0.003588	5.89E-05
Linoleic acid metabolism	0.001557	0.004489	5.25E-05
RIG-I-like receptor signaling pathway	0.001218	0.002794	0.01457
Betalain biosynthesis	0.0011	0.003919	3.15E-05
Polycyclic aromatic hydrocarbon degrada	0.000557	0.0009533	0.001487
Cardiac muscle contraction	0.0002422	0.0001776	0.0482
p53 signaling pathway	0.0001499	6.73E-05	0.01451
Calcium signaling pathway	1.69E-05	3.17E-06	0.03083
Glioma	1.48E-05	1.39E-06	0.02491

Table S5. Different KEGG pathways identified between I-PJS and NI-PJS

pathways (level 3)	I-PJS-Mean	NI-PJS-Mean	P value
Biosynthesis of secondary metaboli	8.459	8.64	0.01644
Pyruvate metabolism	1.024	0.9862	0.03384
2-Oxocarboxylic acid metabolism	0.7644	0.8045	0.0171
Phenylalanine, tyrosine and tryptop	0.6541	0.6896	0.03384
Butanoate metabolism	0.6255	0.5919	0.0434
Propanoate metabolism	0.6058	0.5609	0.02334
Valine, leucine and isoleucine degra	0.1955	0.1775	0.04493
Lysine degradation	0.1972	0.161	0.02334
Tyrosine metabolism	0.1688	0.1491	0.03509
Insulin resistance	0.1296	0.1389	0.009635
Degradation of aromatic compound	0.1491	0.1142	0.03509
Adipocytokine signaling pathway	0.0824	0.09496	0.04493
RNA transport	0.07996	0.0851	0.0434
Ribosome biogenesis in eukaryotes	0.04307	0.04666	0.03264
Proteoglycans in cancer	0.03724	0.04176	0.04191
Naphthalene degradation	0.04573	0.03797	0.02425
Aminobenzoate degradation	0.04582	0.03604	0.01344
Lipoic acid metabolism	0.03929	0.03419	0.04814
Synthesis and degradation of ketone	0.02635	0.02138	0.03637
Amoebiasis	0.02377	0.01981	0.04191
Lipoarabinomannan (LAM) biosynt	0.001032	0.002318	0.03446
Cardiac muscle contraction	0.0002056	0.00014	0.04185
Cholinergic synapse	2.53E-06	2.86E-05	0.03551
Staurosporine biosynthesis	1.44E-06	2.28E-05	0.01591
Vitamin digestion and absorption	2.98E-05	0	0.04802
